# Expansion of the prime editing modality with Cas9 from *Francisella novicida*

**DOI:** 10.1186/s13059-022-02644-8

**Published:** 2022-04-11

**Authors:** Yeounsun Oh, Wi-jae Lee, Junho K. Hur, Woo Jeung Song, Youngjeon Lee, Hanseop Kim, Lee Wha Gwon, Young-Hyun Kim, Young-Ho Park, Chan Hyoung Kim, Kyung-Seob Lim, Bong-Seok Song, Jae-Won Huh, Sun-Uk Kim, Bong-Hyun Jun, Cheulhee Jung, Seung Hwan Lee

**Affiliations:** 1 National Primate Research Center (NPRC), Korea Research Institute of Bioscience and Biotechnology (KRIBB), Cheongju, Korea; 2 Department of Biotechnology, College of Life Sciences and Biotechnology, Korea University, Seoul, Republic of Korea; 3 Department of Bioscience and Biotechnology, Konkuk University, Seoul, Korea; 4 Department of Genetics, College of Medicine, Hanyang University, Seoul, 04763 Republic of Korea; 5 Graduate School of Biomedical Science and Engineering, Hanyang University, Seoul, 04763 Republic of Korea; 6 Department of Life Science, Chung-Ang University, Seoul, 06974 Republic of Korea; 7 Department of Medicine, Major in Medical Genetics, Graduate School, Hanyang University, Seoul, 04763 Republic of Korea; 8 School of Life Sciences and Biotechnology, BK21 Plus KNU Creative BioResearch Group, Kyungpook National University, Daegu, Republic of Korea; 9 Department of Biomolecular Science, KRIBB School of Bioscience, Korea University of Science and Technology, Gajeong-dong, Yuseong-gu, Daejeon, Republic of Korea; 10 Futuristic Animal Resource & Research Center (FARRC), Korea Research Institute of Bioscience and Biotechnology (KRIBB), Cheongju, Korea; 11 Department of Biological Sciences, Chungnam National University, Daejeon, Korea; 12 Department of Functional Genomics, KRIBB School of Bioscience, Korea University of Science and Technology (UST), Daejeon, Korea

**Keywords:** Prime editing, Target expansion, CRISPR-Cas9, Ortholog, *Francisella novicida*

## Abstract

Prime editing can induce a desired base substitution, insertion, or deletion in a target gene using reverse transcriptase after nick formation by CRISPR nickase. In this study, we develop a technology that can be used to insert or replace external bases in the target DNA sequence by linking reverse transcriptase to the *Francisella novicida* Cas9, which is a CRISPR-Cas9 ortholog. Using FnCas9(H969A) nickase, the targeting limitation of existing *Streptococcus pyogenes* Cas9 nickase [SpCas9(H840A)]-based prime editing is dramatically extended, and accurate prime editing is induced specifically for the target genes in human cell lines.

**Supplementary Information:**

The online version contains supplementary material available at 10.1186/s13059-022-02644-8.

## Background

The recent development of reverse transcriptase (RT)-based target DNA editing technology (i.e., prime editing) is based on the SpCas9(H840A) module [1–3]. Since prime editing technology can induce various types of mutations [2, 4–11] compared to base editing [12, 13] that only induces deamination-mediated base substitutions (A to G or C to T), most of the pathogenic mutations reported to date will be corrected without double-stranded DNA cleavage [2]. However, since SpCas9 module-based prime editing technology can modify bases within the 3 bp upstream from the protospacer adjacent motif (PAM) sequence (NGG) on the target DNA, it shows a PAM limitation [14, 15], and still have an off-targeting problem [16]. In order to apply prime editing to various biological systems, it is important to improve the fundamental limitations generated by the CRISPR module. To this end, we tried to accurately induce the mutation in the target sequence using a different type of CRISPR-Cas module and enhanced the function of the original prime editor. Compared to the SpCas9 module [17], FnCas9 (*F. novicida*) [18–21] shows an overhang pattern of double-strand break for the protospacer sequence. Therefore, the FnCas9(H969A) nickase module, which shows a different nicking property on the non-target strand of protospacer, has the advantage of expanding the region recognized as the reverse transcription template (RTT) following the primer binding site (PBS) sequence for prime editing. In this study, we improved the effectiveness limitations of SpCas9(H840A) based prime editor with a new approach using *F. novicida* Cas9, a CRISPR-Cas9 ortholog [20], in human-derived cell lines. Using FnCas9(H969A) linked RT (i.e., FnCas9(H969A)-RT, FnCas9 prime editor) developed in this study, precise genome editing in a range that was not previously applied with SpCas9 is possible. As the number of regions that can be precisely edited using this technology increases, it is expected that FnCas9(H969A)-RT can be applied to various biological systems.

## Results and discussion

**Fig. 1:**
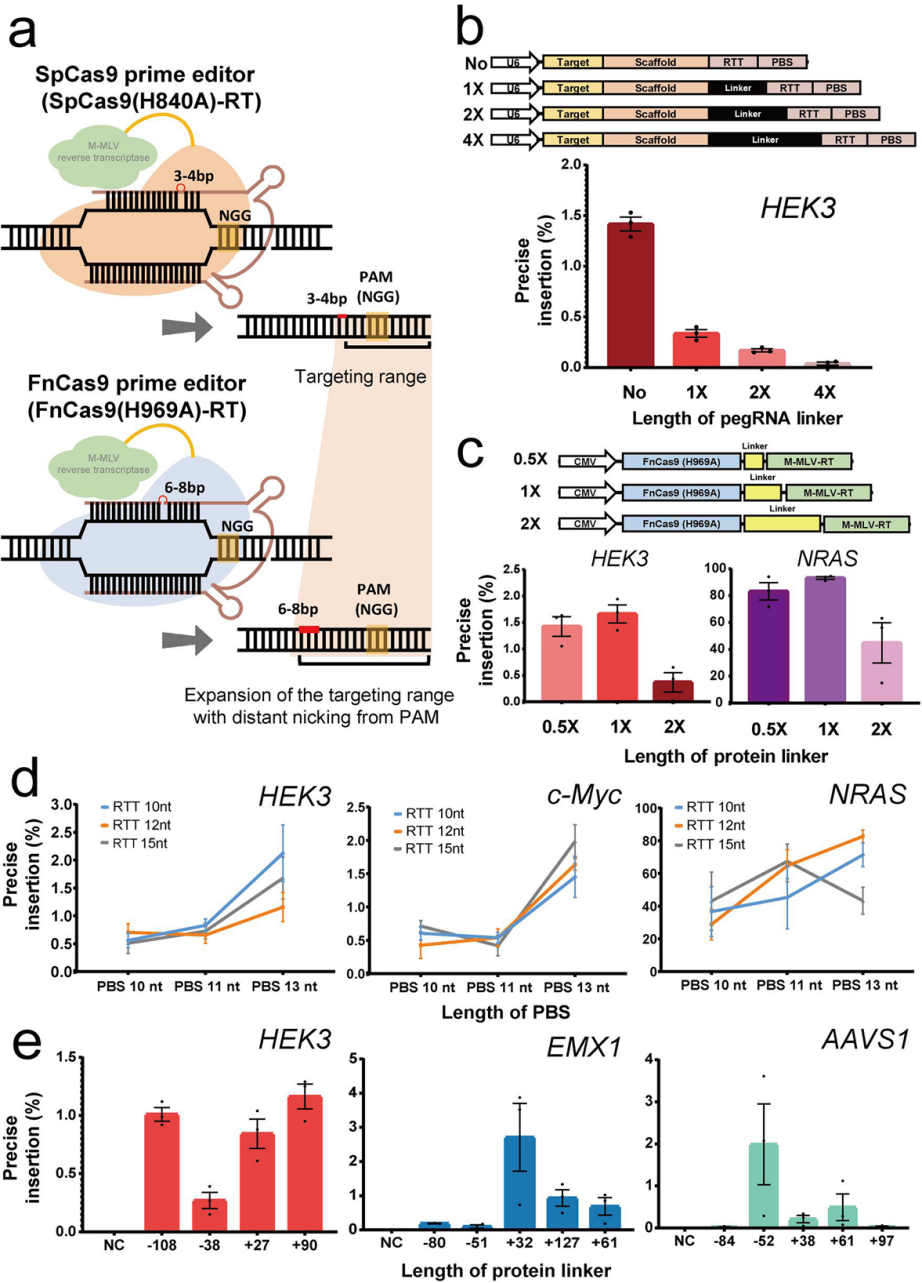

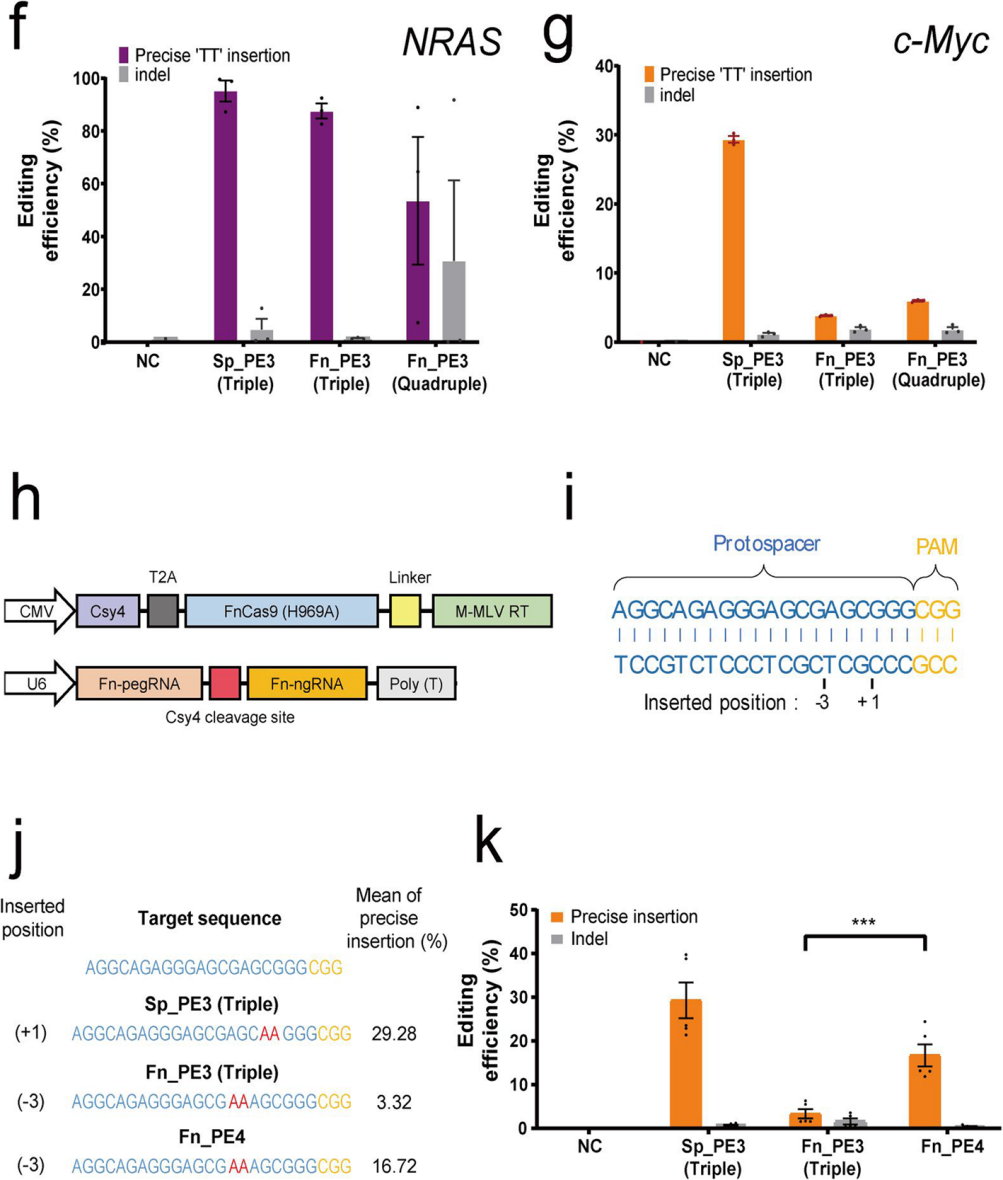
Targeted prime editing and optimization with FnCas9(H969A)-RT. **a** Comparison between SpCas9(H840A) and FnCas9(H969A) nickase-based prime editing. Nicked positions were indicated by red arrows. PAM (NGG) sequence and the targeted base insertion are shown in yellow and red, respectively. **b** pegRNAs for optimizing the prime editing efficiency and comparison of nucleotide insertion efficiency (%) according to linker length (no linker, 1×, 2×, 4× linkers) for *HEK3*. **c** FnCas9(H969A)-RT with various linkers (0.5×, 1×, 2×) and comparison of nucleotide insertion efficiency (%) for *HEK3* and *NRAS*. **d** Targeted prime editing efficiency (%) of FnCas9(H969A)-RT according to the PBS and RTT length in pegRNA on various genes (*HEK3*, *c-Myc*, *NRAS*). **e** Comparison of base insertion efficiency (%) according to ngRNA targeting at various positions (*HEK3*, *c-Myc*, *NRAS*) with the PE3 method of FnCas9 (H969A)-RT. PBS: primer binding site; RTT: reverse transcription template. **f**, **g** Comparison of target-specific nucleotide insertion into *NRAS* (**f**) and *c-Myc* (**g**) genes by SpCas9(H840A)-RT and FnCas9(H969A)-RT. PE3-Triple/Quadruple: Detailed information is in the (Additional file 1: Table S3). **h** Schematic of a PE4 strategy using Csy4 mediated pegRNA and ngRNA expression for FnCas9(H969A)-RT. Detailed information is in the (Additional file 1: Table S3). **i** Target sequence in the *c-Myc* gene commonly targeted by prime editors. Protospacers and PAM(NGG) sequences are shown in blue and yellow, respectively. Inserted positions are indicated at the bottom of the target sequence. **j** The result of site-specific base insertion induced by SpCas9(H840A)-RT and FnCas9(H969A)-RT using PE3 or PE4 form delivery. Inserted sequence AA are shown in red. Each position and efficiency(%) of AA insertions are indicated to the left and right of the target sequence, respectively. **k** Comparison of the efficiency (%) of base insertion according to (**j**). Each histogram was plotted by applying standard error of the mean values to repeated experimental values (*n* ≥ 3). *P* values are calculated using a two-way ANOVA, Dunnett test (ns: not significant, *P = 0.0332, **P = 0.0021, ***P = 0.0002, ****P < 0.0001)

For the application of prime editing with a new ortholog, the characteristics of FnCas9 were compared and analyzed with that of generally used SpCas9. First, wild-type FnCas9 and SpCas9 were purified in the form of recombinant protein, and cleavage experiments were performed on PCR amplicons including various target nucleotide sequences in vitro (Additional file 1: Fig. S1). At this time, FnCas9 and SpCas9 recognized the same PAM (NGG) for each target nucleotide sequence, and we directly compared the cleavage results using a protospacer of the same length. In both FnCas9 and SpCas9, it was confirmed that cleavage occurred at 3bp in front of PAM (NGG) in common for TS (target strand) (Additional file 1: Fig. S1). On the other hand, for NTS (non-target strand), FnCas9 cleavage occurred at 6–8 bp upstream of PAM (NGG) and SpCas9 cleavage occurred at 3–4 bp upstream PAM (NGG), respectively. Based on the cleavage property, we sought to test the target-specific prime editing with FnCas9(H969A)-RT for various target sites (Additional file 1: Fig. S2) in human-derived cell lines (Fig. 1a). First, an RT enzyme was connected to the FnCas9(H969A) nickase module to optimize the performance of the prime editor on the target gene. To optimize the efficiency of the newly applied FnCas9 prime editor, FnCas9(H969A)-RT or prime editing guide RNA (pegRNA) with various linker lengths were prepared (Fig. 1b, c top, Additional file 1: Table S1). The pegRNA was constructed to allow for insertion of a “TT” bi-nucleotide sequence at the expected cleavage point [6 and 3 nucleotides upstream from the PAM (NGG) sequence for Fn and Sp, respectively] for the endogenous locus by considering the nicking point on each target sequence based on SpCas9 or FnCas9 nickase modules. The HEK293FT cell line was then co-transfected with pegRNA/FnCas9(H969A)-RT and nicking-guide RNA (ngRNA) expression plasmids (Additional file 1: Fig. S3). We found that the bi-nucleotide TT was accurately inserted in the genomic DNA (*HEK3*, *NRAS*) at a site of 6 nucleotides upstream from the PAM in the target sequence when using FnCas9(H969A)-RT compared to SpCas9(H840A)-RT as expected (Fig. 1b, c, Additional file 1: Fig. S4). During the optimization, pegRNA without a linker showed the highest efficiency in *HEK3* locus (Fig. 1b), and optimized TT insertion was achieved when using the FnCas9 prime editor with a 1× linker length for *HEK3* and *NRAS* locus (Fig. 1c).

To confirm the effect of the length of the PBS and RTT in pegRNA on prime editing efficiency, several candidate pegRNAs with different lengths of PBS and RTT were prepared (Additional file 1: Table S1), and a TT insertion was tested at various genes (*HEK3*, *c-Myc*, *NRAS*) in human cell lines (Fig. 1d, Additional file 1: Fig. S4a, c, e). The external TT insertion efficiency at the *HEK3* site increased as the length of the PBS increased (Fig. 1d). FnCas9(H969A)-RT resulted in 1.65% (*HEK3*), 1.69% (*c-Myc*), and 65.77% (*NRAS*) of precise TT insertion [6 bp upstream from PAM (NGG)] compared to SpCas9(H840A)-RT on average (Fig. 1d, Additional file 1: Fig. S4b, d, f), and the efficiency slightly varied according to the length of RTT. Notably, the TT insertion efficiency was significantly different for each targeted gene (*HEK3*, *EMX1*, *AAVS1*) according to the location of the nicking guide RNA (Fig. 1e, Additional file 1: Table S1) on the target-strand side when applying the prime editing with PE3 manner (Additional file 1: Fig. S3). Next, we compared the optimized FnCas9(H969A)-RT with SpCas9(H840A)-RT for specific loci (*NRAS*, *c-Myc*) (Fig. 1f, g). Next-generation sequencing (NGS) analysis (Additional file 1: Table S2) showed that compared to SpCas9-RT (29.34–95.18%), FnCas9-RT exhibited greater variation in TT insertion efficiency (3.82–87.56%), which depends on the target sequences (Fig. 1f, g) and cell types (Additional file 1: Fig. S5). These results may reflect the weaker nicking property of FnCas9(H969A) compared to SpCas9(H840A) which is shown in double nicking experiment (Additional file 1: Fig. S6). To overcome the problem of low prime editing, the use of Csy4 RNA endonuclease was applied to peg RNA (Fig. 1h–k), [22]. First, a system capable of inducing independent expression of Csy4 RNA endonuclease and FnCas9(H969A)-RT was constructed (hereafter PE4 delivery method), then the pegRNA and ngRNA were linked through the Csy4 recognition site so that they could act simultaneously in the cell (Fig. 1h). When comparing the efficiency of bi-nucleotide AA insertion by PE3 and PE4 methods based on FnCas9(H969A)-RT by targeting *c-Myc* gene in HEK293FT cell line (Fig. 1i), it was confirmed that AA bases were correctly and efficiently inserted at the expected positions (Fig. 1j, k). When precise editing was induced in the target genes (*N*=48) by using FnCas9(H969A)-RT in PE4 manner, the averaged efficiency increased 2,94 fold compared to the conventional PE3 method (Additional file 1: Fig. S2d, S7).

Based on the sensitivity to mis-matched sequences of the FnCas9 [18], the editing efficiency (%) was analyzed for off-target candidates which predicted from in silico analysis (Additional file 1: Table S3). Indeed, when analyzing the off-target effect of the FnCas9-based prime editor for each target gene, we found that no significant off-target mutation was observed by FnCas9(H969A)-RT (Additional file 1: Fig. S8). We further examined whether the optimized FnCas9(H969A)-RT can be used in combination with SpCas9(H840A)-RT to induce prime editing simultaneously on the target gene (Additional file 1: Fig. S9). The TT insertion was respectively induced by FnCas9(H969A)-RT and SpCas9(H840A)-RT in two adjacent nucleotide sequences on the *NRAS*, *VEGFA* gene (Additional file 1: Fig. S9a, b). The overall rate of simultaneous TT insertion by the two prime editors was relatively lower (0.02–1.3%) than single TT insertion (Additional file 1: Fig. S9c, d).

**Fig. 2:**
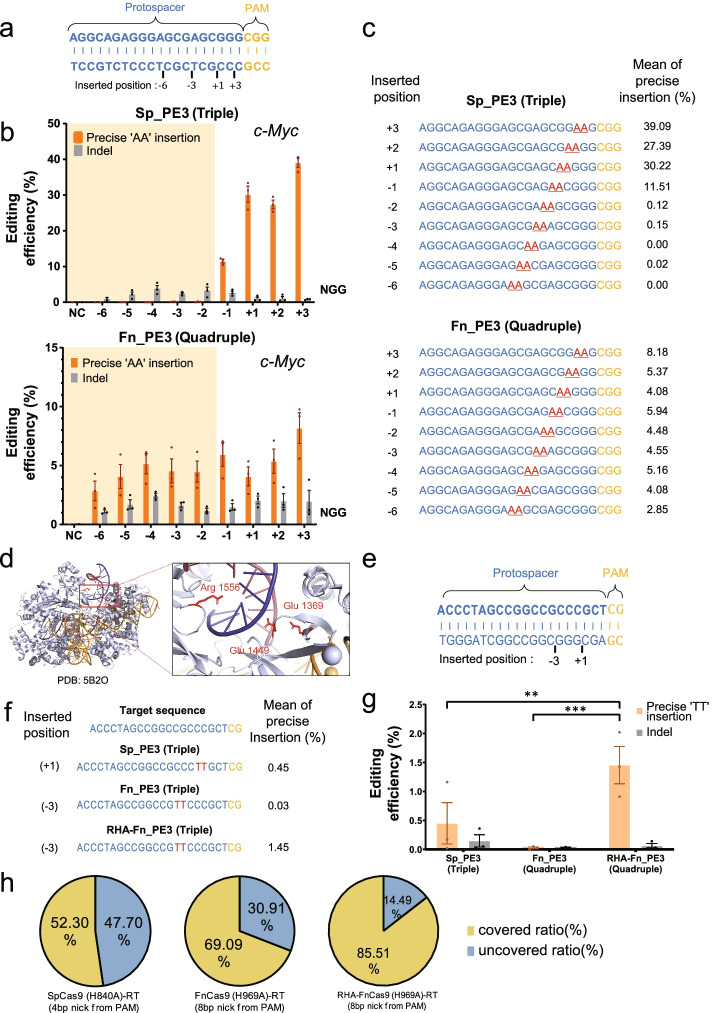
Expansion of the targetable range of prime editing by using FnCas9(H969A)-RT. **a** Schematics of the target gene (*c-Myc*) recognized by SpCas9(H840A)-RT and FnCas9(H969A)-RT. **b** Direct comparison of the efficiency (%) of base insertion by SpCas9(H840A)-RT (Top) and FnCas9(H969A)-RT (Bottom). **c** NGS result of site-specific base insertion induced by SpCas9(H840A)-RT and FnCas9(H969A)-RT. Each position and efficiency (%) in which the inserted AA bases are indicated to the left and right of the target sequence, respectively. **d** Engineered residues (Glu 1369, Glu 1449, Arg 1556) in FnCas9 for YG PAM recognition (PDB: 5B2O). **e** Target sequence for YG PAM recognition in the *c-Myc* gene commonly targeted using SpCas9(H840A)-RT, FnCas9(H969A)-RT and engineered FnCas9(H969A, E1369R, E1449H, R1556A)-RT. **f** The result of site-specific base insertion at YG PAM target induced by SpCas9(H840A)-RT and engineered FnCas9(H969A, E1369R, E1449H, R1556A)-RT using PE3 form delivery. **g** Comparison of the efficiency (%) of base insertion at YG(Y = C, T) PAM target by using SpCas9(H840A)-RT and engineered FnCas9(H969A, E1369R, E1449H, R1556A)-RT according to (**f**). *P* values are calculated using a two-way ANOVA, Dunnett test (ns: not significant, *P = 0.0332, **P = 0.0021, ***P = 0.0002, ****P < 0.0001). **h** Phi-chart demonstration for pathogenic SNP coverage of SpCas9(H840A)-RT or FnCas9(H969A)-RT prime editing. The nicking point counted as (N) bp upstream from the PAM (NGG or YG) is indicated at the bottom of the phi chart. Covered ratio (%) = targetable SNP number/total SNP number ×100, uncovered ratio (%) = 100 − covered ratio (%). Each histogram was plotted by applying standard error of the mean values to repeated experimental values (*n* = 3)

Next, according to the difference of nicking property, we directly compared the range that can induce prime editing based on FnCas9-RT and SpCas9-RT for common target sequence (Fig. 2). In HEK293FT cells, the efficiency of prime editing was compared for the same target sequence in the *c-Myc* (Fig. 2a–c) and *EMX1* (Additional file 1: Fig. S10) genes. The point at which the base is inserted (AA for *c-Myc*, TT for *EMX1*) was indicated according to the point at which nicks were formed (from − 6 to + 3) by both nickase modules (Fig. 2a, Additional file 1: Fig. S10a). SpCas9(H840A)-RT showed an editing efficiency of 27–39% for the AA insertion at *c-Myc* from the − 1 to +3 position in the sequence based on the nick position (Fig. 2b, c, top). By contrast, FnCas9(H969A)-RT showed a 2–8% insertion efficiency from position − 6 to +3 based on the nick position (Fig. 2b, c, bottom). Versatile editing showed that various base substitutions and insertions were possible from − 3 to +3 positions for each of *NRAS* (Additional file 1: Fig. S11a-b) and *c-Myc* (Additional file 1: Fig. S11c-d) genes by using FnCas9(H969A)-RT. Similar results were found for the TT insertion efficiency for the same target sequence in the *EMX1* gene (Additional file 1: Fig. S10), in which the range of genome editing by FnCas9(H969A)-RT was significantly expanded compared to that of SpCas9(H840A)-RT editing. In addition to this, prime editing range was further expanded by the engineering of the FnCas9 protein for relaxed PAM recognition (Fig. 2d–g). Based on the previously published RHA-FnCas9 (E1369R, E1449H, R1556A) effector (Fig. 2d) [21], we compared the prime editing efficiency of SpCas9(H840A)-RT, FnCas9(H969A)-RT, and RHA-FnCas9(H969A)-RT for the target sequence having YG PAM in the *c-Myc* gene (Fig. 2e). In the case of prime editing using RHA-FnCas9(H969A)-RT, it was possible to insert a TT base into a position that could not be inserted by SpCas9(H840A)-RT (Fig. 2f). As shown in data, the editing efficiency of the target-specific TT base insertion was significantly increased compared to when SpCas9(H840A)-RT or FnCas9(H969A)-RT was used (Fig. 2g). Based on the above results, in silico-based comparative analysis was performed to compare and analyze how much (%) the pathogenic mutations known in ClinVar (NCBI) database can be recovered through SpCas9(H840A)-RT, FnCas9(H969A)-RT, and RHA-FnCas9(H969A)-RT, respectively (Fig. 2h, Additional file 1: Fig. S12). These results indicate that the use of RHA-FnCas9(H969A)-RT by protein engineering can cover a significant portion (85.51%) of previously known pathogenic mutations.

## Conclusions

In this study, we confirmed that the editable region of prime editing could be extended by different nicking properties of CRISPR-Cas orthologs and engineering the PAM recognition domain within the Cas9 nickase. In summary, as a Cas9 ortholog-based prime editor, FnCas9(H969A)-RT shows a precise, expanded range of prime editing and versatile editing properties. These advantages of prime editor are expected to have a big ripple effect on the bio/medical field by enabling target-specific and various types of gene editing in the future. In particular, further engineering of Cas9 protein or AAV based delivery of the FnCas9(H969A)-RT is possibly applied to most of the existing human disease-causing mutations, and it is thought that FnCas9 based prime editing will increase the possibility of application as a therapeutic agent by compensating the role of SpCas9 based one.

## Methods

### Purification of SpCas9 and FnCas9 proteins

Subcloned pET28a-SpCas9 and pET28a-FnCas9 bacterial expression vectors were transformed into *Escherichia coli* BL21(DE3) cells, respectively, and colonies were grown at 37 °C. After growing to an optical density of 0.6 in a 500-ml culture flask, induction was performed at 18 °C for 48 h by isopropyl β-d-1-thiogalactopyranoside treatment. The cells were then precipitated by centrifugation and resuspended in lysis buffer [20 mM Tris-HCl (pH 8.0), 300 mM NaCl, 10 mM β-mercaptoethanol, 1% Triton X-100, and 1 mM phenylmethylsulfonyl fluoride (PMSF)]. The cells were disrupted by sonication on ice for 3 min, and the cell lysates were separated by centrifugation at 20,000×*g* for 10 min. The further purification process was carried out as previously described [23]. Each purified SpCas9 and FnCas9 protein was replaced with a Centricon filter (Amicon Ultra) as a storage buffer [200 mM NaCl, 50 mM HEPES (pH 7.5), 1 mM dithiothreitol (DTT), and 40% glycerol] for long-term storage at − 80°C. The purity of the purified proteins was confirmed by sodium dodecyl sulfate (SDS)-polyacrylamide gel electrophoresis (8–10%), and protein activity was tested using an in vitro polymerase chain reaction (PCR) amplicon cleavage assay.

### In vitro transcription for guide RNA synthesis

For in vitro transcription, DNA oligos containing an sgRNA sequence (Additional file 1: Table S4) corresponding to each target sequence were purchased from Cosmo Genetech. After extension PCR (denaturation at 98 °C for 30 s, primer annealing at 62 °C for 10 s, and elongation at 72 °C for 10 s, 35 cycles) using the DNA oligos, DNA was purified using GENECLEAN® Turbo Kit (MP Biomedicals). The purified template DNA was mixed with an in vitro transcription mixture [T7 RNA polymerase (NEB), 50 mM MgCl_2_, 100 mM rNTPs (rATP, rGTP, rUTP, rCTP), 10× RNA polymerase reaction buffer, murine RNase inhibitor (NEB),100 mM DTT, and DEPC) and incubated at 37 °C for 8 h. The DNA template was completely removed by incubation with DNase I (NEB) at 37 °C for 1 h, and the RNA was further purified using GENECLEAN® Turbo Kit (MP Biomedicals) for later use. The purified RNA was concentrated by lyophilization (20,000×*g* at − 55 °C for 1 h) and stored at – 80 °C.

### In vitro cleavage assay and DNA sequencing for nicking point analysis

Using the purified FnCas9 or SpCas9 recombinant protein, an in vitro cleavage assay was performed to determine the location of nicks in the non-target strand of the DNA sequence to be prime-edited. Each target site was cloned into a T-vector, and then T-vectors were purified and incubated in cleavage buffer (NEB3, 10 μl) with FnCas9 at 37°C for 1 h. The cleavage reaction was stopped by adding a stop buffer (100 mM Ethylenediaminetetraacetic (EDTA) acid, 1.2% SDS), and only the plasmid DNA was separated through the column (Qiagen, QIAquick® PCR Purification Kit). The cleavage point of FnCas9 or SpCas9 was confirmed by run-off sequencing analysis of the cut fragments. The last nucleotide sequence was confirmed by the A-tailing of polymerase and the cleavage pattern was analyzed by comparative analysis with reference sequences.

### Design and cloning of the prime editor and pegRNA expression vectors

The cytomegalovirus promoter-based SpCas9(H840A)-RT and FnCas9(H969A)-RT expression vectors were constructed to induce genome editing in human cell lines. To optimize the efficiency of the newly developed FnCas9 prime editor, FnCas9 prime editors with various linker lengths were prepared or the linker length of pegRNA was diversified (Additional file 1: Table S1). In addition, pegRNA containing the corresponding nucleotide sequence (TT or AA) was prepared so that the nucleotide could be inserted into the target nucleotide sequence (*HEK3*, *AAVS1*, *c-Myc*, *NRAS*, *EMX1*). The PBS and RTT regions in pegRNA were designed and produced according to the non-target strand nick site generated by the FnCas9 nickase module (Additional file 1: Fig. S1). As a positive control, the pegRNA of SpCas9-RT was constructed so that a TT base was inserted 3 bp in front of the PAM in consideration of the nicking point. By contrast, in FnCas9-RT, pegRNA was constructed so that the TT base was inserted at the expected cleavage point (6 bp upstream from the PAM). The pegRNA expression vector is driven by the *U6* promoter and was designed and manufactured so that only the protospacer, PBS, and RTT can be replaced with restriction enzymes according to the target nucleotide sequence.

### Cell culture and transfection

Human-derived cell lines (HEK293FT, K562, U2OS, and HeLa) were purchased from Invitrogen (R70007), and American Type Culture Collection (CCL-243, HTB-96, CCL-2), respectively. The HEK293FT, U2OS, and HeLa human cells were maintained in Dulbecco’s modified Eagle’s medium (DMEM) with 10% fetal bovine serum (both from Gibco) at 37 °C in the presence of 5% CO_2_. K562 cells were maintained in Iscove’s modified Dulbecco’s medium (IMDM) with 10% FBS (Gibco) at 37 °C in the presence of 5% CO_2_. Cells were subcultured every 48 h to maintain 70% confluency. For target sequence editing, 2 × 10^5^ HEK293FT or HeLa cells were transfected with plasmids expressing pegRNAs (240 pmol), the prime editor expression plasmid [SpCas9(H840A)-RT (Addgene no. 132775), FnCas9(H969A)-RT (developed in this study)], and nicking sgRNA (60 pmol) via electroporation using an Amaxa electroporation kit (V4XC-2032; program: CM-130 for HEK293FT cells, FF-120 for K562 cells, DN-100 for U2OS cells, and CN-114 for HeLa cells). In parallel with the SpCas9 prime editor, the FnCas9 prime editor was transfected targeting the same nucleotide sequence in HEK293FT cells. Transfected cells were transferred to a 24-well plate containing DMEM (500 μl/well), pre-incubated at 37 °C in the presence of 5% CO_2_ for 30 min, and incubated under the same conditions for subculture. All the cell lines used in this study have been authenticated.

### Purification of genomic DNA and construction of target site amplicons

Genomic DNA (gDNA) was extracted from the cultured cells 72 h after genome editing. The gDNA was isolated using DNeasy Blood and Tissue Kit (Qiagen). Target amplicons were obtained through PCR (denaturation at 98 °C for 30 s, primer annealing at 58°C for 30 s, elongation at 72 °C for 30 s, 35 cycles; Additional file 1: Table S2) from gDNA extracted from cells treated with each SpCas9 or FnCas9 prime editor. Targeted amplicon next-generation sequencing was performed using nested PCR (denaturation: 98 °C for 30 s, primer annealing: 58 °C for 30 s, elongation: 72 °C for 30 s, 35 cycles) to analyze the efficiency of base (TT or AA) insertion.

### Targeted amplicon sequencing and data analysis

To prepare the targeted amplicon library, gDNA was extracted from the cells and further amplified using DNA primers (Additional file 1: Table S2). Nested PCR (denaturation at 98°C for 30 s, primer annealing at 62°C for 15 s, and elongation at 72 °C for 15 s, 35 cycles) was performed to conjugate adapter and index sequences to the amplicons. All targeted amplicon sequencing and data analysis were performed as suggested in a previous study [23].

### In silico-based pathogenic SNP coverage analysis

For analyses of effective windows of FnCas9 and SpCas9, position information of pathogenic single point mutations was extracted from ClinVar (NCBI) data. Next, the position information was compared with the PAM sequences of FnCas9 and SpCas9 within human genome (GRCh37, GRCh38, http://ftp.ensembl.org/pub/release-100/fasta/homo_sapiens/dna/). Reverse complementary sequences of PAM such as CCN for NGG are also included for the analyses, and Y chromosome and mitochondrial sequences were excluded. The analyses process was conducted using R software (version 4.1.1).

## Supplementary Information

**Additional file 1: Figure S1.** Analysis of the cleavage point of FnCas9 and SpCas9. **Figure S2.** Shared target sequence and editing method by using SpCas9(H840A)-RT and FnCas9(H969A)-RT. **Figure S3.** Various methods of prime editor delivery by using SpCas9(H840A)-RT or FnCas9(H969A)-RT expression plasmids. **Figure S4.** The target sequence and NGS analysis results commonly edited by SpCas9(H840A)-RT or FnCas9(H969A)-RT. **Figure S5.** Cell diversity experiment of prime editing using FnCas9(H969A)-RT. **Figure S6.** Comparative analysis of nickase activity of SpCas9(H840A)-RT and FnCas9(H969A)-RT. **Figure S7.** Targeting of multiple loci for direct comparison between SpCas9(H840A)-RT and FnCas9(H969A)-RT based prime editing. **Figure S8.** Comparative analysis of off-target editing of SpCas9(H840A)-RT and FnCas9(H969A)-RT based on in-silico predicted sites. **Figure S9.** Multiplexed prime editing with SpCas9(H840A)-RT and FnCas9(H969A)-RT system. **Figure S10.** Expansion of the range of prime editing in *EMX1* locus by FnCas9(H969A)-RT. **Figure S11.** Result of the prime editing in target sequence induced by FnCas9(H969A)-RT. **Figure S12.** Expansion of the targetable range by prime editing with FnCas9(H969A)-RT or RHA-FnCas9(H969A)-RT.

**Additional file 2.** Review history.

## Data Availability

Targeted deep sequencing data are available at NCBI Sequence Read Archive (SRA) under accession number PRJNA736246 [24]. The source code used for SNP analysis is released under an open source license (GNU General Public License V2.0) and available at https://github.com/yeounsunoh/Prime-editing-ClinVar-coverage-data.git [25]. The source code is archived in zenodo under [26].
