# Supplementary material for: Expansion of the prime editing modality with Cas9 from Francisella novicida
Source: Genome Biol. 2022 Apr 11;23:92. doi: 10.1186/s13059-022-02644-8 (PMC8996390; doi:10.1186/s13059-022-02644-8)
Supplement: Supplementary file 1 — Additional file 1: Figure S1. Analysis of the cleavage point of FnCas9 and SpCas9. Figure S2. Shared target sequence and editing method by using SpCas9(H840A)-RT and FnCas9(H969A)-RT. Figure S3. Various methods of prime editor delivery by using SpCas9(H840A)-RT or FnCas9(H969A)-RT expression plasmids. Figure S4. The target sequence and NGS analysis results commonly edited by SpCas9(H840A)-RT or FnCas9(H969A)-RT. Figure S5. Cell diversity experiment of prime editing using FnCas9(H969A)-RT. Figure S6. Comparative analysis of nickase activity of SpCas9(H840A)-RT and FnCas9(H969A)-RT. Figure S7. Targeting of multiple loci for direct comparison between SpCas9(H840A)-RT and FnCas9(H969A)-RT based prime editing. Figure S8. Comparative analysis of off-target editing of SpCas9(H840A)-RT and FnCas9(H969A)-RT based on in-silico predicted sites. Figure S9. Multiplexed prime editing with SpCas9(H840A)-RT and FnCas9(H969A)-RT system. Figure S10. Expansion of the range of prime editing in EMX1 locus by FnCas9(H969A)-RT. Figure S11. Result of the prime editing in target sequence induced by FnCas9(H969A)-RT. Figure S12. Expansion of the targetable range by prime editing with FnCas9(H969A)-RT or RHA-FnCas9(H969A)-RT. [file 13059_2022_2644_MOESM1_ESM.pdf]

Expansion of the prime editing modality with Cas9 from Francisella novicida

Yeounsun Oh#, Wi-jae Lee#, Junho K. Hur#, WooJeung Song, Youngjeon Lee, Hanseop Kim, Lee Wha Gwon, Young-Hyun Kim, Young-Ho Park, Chan Hyoung Kim, Kyung-Seob Lim, Bong-Seok Song, Jae-Won Huh, Sun-Uk Kim, Bong-Hyun Jun, Cheulhee Jung*, Seung Hwan Lee*

Supplementary Tables

Table S1. Sequence information of pegRNAs and sgRNAs used in this study. Complement bases for inserted DNA sequences and different length of linker sequences in pegRNA are shown in red and green, respectively. PAM sequences (NGG) in the target DNA are shown in orange.

pegRNA	Target(protospacer) sequence (5' to 3')	3' extension (5' to 3')	PBS length (nt)	RTT length (nt)
HEK3_target1_no linker_Fn	GGCCCAGACTGAGCACGTGATGG	CATCACGTaaGCTCAGTCTGGGC	13	10
HEK3_target1_1X linker_Fn	GGCCCAGACTGAGCACGTGATGG	TTTCCATCACGTaaGCTCAGTCTGGGC	13	10
HEK3_target1_2X linker_Fn	GGCCCAGACTGAGCACGTGATGG	TTTCTTTCCATCACGTaaGCTCAGTCTGGGC	13	10
HEK3_target1_4X linker_Fn	GGCCCAGACTGAGCACGTGATGG	TTTCTTTCTTTCTTTCCATCACGTaaGCTCAGTCTGGGC	13	10
HEK3_target1_Fn	GGCCCAGACTGAGCACGTGATGG	CATCACGTaaGCTCAGTCTGGGC	13	10
NRAS_Fn	GGTAAGGGGGCAGGGAGGGAGGG	CCTCCCTCaaCCTGCCCCCTTAC	13	10
HEK3_target1_10-10_Fn	GGCCCAGACTGAGCACGTGATGG	CATCACGTaaGCTCAGTCTG	10	10
HEK3_target1_10-12_Fn	GGCCCAGACTGAGCACGTGATGG	GCCATCACGTaaGCTCAGTCTG	10	12
HEK3_target1_10-15_Fn	GGCCCAGACTGAGCACGTGATGG	TCTGCCATCACGTaaGCTCAGTCTG	10	15
HEK3_11-10_Fn	GGCCCAGACTGAGCACGTGATGG	CATCACGTaaGCTCAGTCTGG	11	10
HEK3_target1_11-12_Fn	GGCCCAGACTGAGCACGTGATGG	GCCATCACGTaaGCTCAGTCTGG	11	12
HEK3_target1_11-15_Fn	GGCCCAGACTGAGCACGTGATGG	TCTGCCATCACGTaaGCTCAGTCTGG	11	15
HEK3_target1_13-10_Fn	GGCCCAGACTGAGCACGTGATGG	CATCACGTaaGCTCAGTCTGGGC	13	10
HEK3_target1_13-12_Fn	GGCCCAGACTGAGCACGTGATGG	GCCATCACGTaaGCTCAGTCTGGGC	13	12
HEK3_target1_13-15_Fn	GGCCCAGACTGAGCACGTGATGG	TCTGCCATCACGTaaGCTCAGTCTGGGC	13	15
c-Myc_10-10_Fn	AGGCAGAGGGAGCGAGCGGGCGG	CGCCCGCTaaCGCTCCCTCT	10	10
c-Myc_10-12_Fn	AGGCAGAGGGAGCGAGCGGGCGG	GCCGCCCGCTaaCGCTCCCTCT	10	12
c-Myc _10-15_Fn	AGGCAGAGGGAGCGAGCGGGCGG	CCGGCCGCCCGCTaaCGCTCCCTCT	10	15
c-Myc_11-10_Fn	AGGCAGAGGGAGCGAGCGGGCGG	CGCCCGCTaaCGCTCCCTCTG	11	10
c-Myc_11-12_Fn	AGGCAGAGGGAGCGAGCGGGCGG	GCCGCCCGCTaaCGCTCCCTCTG	11	12
c-Myc_11-15_Fn	AGGCAGAGGGAGCGAGCGGGCGG	CCGGCCGCCCGCTaaCGCTCCCTCTG	11	15

c-Myc_13-10_Fn	AGGCAGAGGGAGCGAGCGGGCGG	CGCCCGCTaaCGCTCCCTCTGCC	13	10
c-Myc_13-12_Fn	AGGCAGAGGGAGCGAGCGGGCGG	GCCGCCCGCTaaCGCTCCCTCTGCC	13	12
c-Myc_13-15_Fn	AGGCAGAGGGAGCGAGCGGGCGG	CCGGCCGCCCGCTaaCGCTCCCTCTGCC	13	15
NRAS_10-10_Fn	GGTAAGGGGGCAGGGAGGGAGGG	CCTCCCTCaaCCTGCCCCCT	10	10
NRAS_10-12_Fn	GGTAAGGGGGCAGGGAGGGAGGG	TCCCTCCCTCaaCCTGCCCCCT	10	12
NRAS_10-15_Fn	GGTAAGGGGGCAGGGAGGGAGGG	ACTTCCCTCCCTCaaCCTGCCCCCT	10	15
NRAS_11-10_Fn	GGTAAGGGGGCAGGGAGGGAGGG	CCTCCCTCaaCCTGCCCCCTT	11	10
NRAS_11-12_Fn	GGTAAGGGGGCAGGGAGGGAGGG	TCCCTCCCTCaaCCTGCCCCCTT	11	12
NRAS_11-15_Fn	GGTAAGGGGGCAGGGAGGGAGGG	ACTTCCCTCCCTCaaCCTGCCCCCTT	11	15
NRAS_13-10_Fn	GGTAAGGGGGCAGGGAGGGAGGG	CCTCCCTCaaCCTGCCCCCTTAC	13	10
NRAS_13-12_Fn	GGTAAGGGGGCAGGGAGGGAGGG	TCCCTCCCTCaaCCTGCCCCCTTAC	13	12
NRAS_13-15_Fn	GGTAAGGGGGCAGGGAGGGAGGG	ACTTCCCTCCCTCaaCCTGCCCCCTTAC	13	15
c-Myc _Sp_+3	AGGCAGAGGGAGCGAGCGGGCGG	TAGCCGGCCGCttCCGCTCGCTCCCTCT	13	15
c-Myc _Sp_+2	AGGCAGAGGGAGCGAGCGGGCGG	TAGCCGGCCGCCttCGCTCGCTCCCTCT	13	15
c-Myc _Sp_+1	AGGCAGAGGGAGCGAGCGGGCGG	TAGCCGGCCGCCCttGCTCGCTCCCTCT	13	15
c-Myc _Sp_-1	AGGCAGAGGGAGCGAGCGGGCGG	TAGCCGGCCGCCCGttCTCGCTCCCTCT	13	15
c-Myc _Sp_-2	AGGCAGAGGGAGCGAGCGGGCGG	TAGCCGGCCGCCCGCttTCGCTCCCTCT	13	15
c-Myc _Sp_-3	AGGCAGAGGGAGCGAGCGGGCGG	TAGCCGGCCGCCCGCTttCGCTCCCTCT	13	15
c-Myc _Sp_-4	AGGCAGAGGGAGCGAGCGGGCGG	TAGCCGGCCGCCCGCTCttGCTCCCTCT	13	15
c-Myc _Sp_-5	AGGCAGAGGGAGCGAGCGGGCGG	TAGCCGGCCGCCCGCTCGttCTCCCTCT	13	15
c-Myc _Sp_-6	AGGCAGAGGGAGCGAGCGGGCGG	TAGCCGGCCGCCCGCTCGCttTCCCTCT	13	15
c-Myc _Fn_+3	AGGCAGAGGGAGCGAGCGGGCGG	CCGGCCGCttCCGCTCGCTCCCTCTGCC	13	15
c-Myc _Fn_+2	AGGCAGAGGGAGCGAGCGGGCGG	CCGGCCGCCttCGCTCGCTCCCTCTGCC	13	15
c-Myc _Fn_+1	AGGCAGAGGGAGCGAGCGGGCGG	CCGGCCGCCCttGCTCGCTCCCTCTGCC	13	15
c-Myc _Fn_-1	AGGCAGAGGGAGCGAGCGGGCGG	CCGGCCGCCCGttCTCGCTCCCTCTGCC	13	15
c-Myc _Fn_-2	AGGCAGAGGGAGCGAGCGGGCGG	CCGGCCGCCCGCttTCGCTCCCTCTGCC	13	15
c-Myc _Fn_-3	AGGCAGAGGGAGCGAGCGGGCGG	CCGGCCGCCCGCTttCGCTCCCTCTGCC	13	15
c-Myc _Fn_-4	AGGCAGAGGGAGCGAGCGGGCGG	CCGGCCGCCCGCTCttGCTCCCTCTGCC	13	15
c-Myc _Fn_-5	AGGCAGAGGGAGCGAGCGGGCGG	CCGGCCGCCCGCTCGttCTCCCTCTGCC	13	15
c-Myc _Fn_-6	AGGCAGAGGGAGCGAGCGGGCGG	CCGGCCGCCCGCTCGCttTCCCTCTGCC	13	15
c-Myc _G to A sub_Fn	AGGCAGAGGGAGCGAGCGGGCGG	GCCGtCCGCTCGCTCCCTCTGCC	13	10
c-Myc_+3 A to G sub_Fn	AGGCAGAGGGAGCGAGCGGGCGG	GCCGCCCGCcCGCTCCCTCTGCC	13	10
c-Myc_-3 A to C sub_Fn	AGGCAGAGGGAGCGAGCGGGCGG	GCCGCCCGCgCGCTCCCTCTGCC	13	10
c-Myc_-1 C to T sub_Fn	AGGCAGAGGGAGCGAGCGGGCGG	GCCGCCCaCTCGCTCCCTCTGCC	13	10
c-Myc_-3 AA ins_Fn	AGGCAGAGGGAGCGAGCGGGCGG	CGCCCGCTttCGCTCCCTCTGCC	13	10
c-Myc_-3 TT ins_Fn	AGGCAGAGGGAGCGAGCGGGCGG	CGCCCGCTaaCGCTCCCTCTGCC	13	10
c-Myc_-3 CC ins_Fn	AGGCAGAGGGAGCGAGCGGGCGG	CGCCCGCTggCGCTCCCTCTGCC	13	10
c-Myc_-3 GG ins_Fn	AGGCAGAGGGAGCGAGCGGGCGG	CGCCCGCTccCGCTCCCTCTGCC	13	10
NRAS_Sp	ATCCAGTATGTCCAACAAACAGG	AACCTGTTaaTGTTGGACATACT	13	10
NRAS_Fn	GGTAAGGGGGCAGGGAGGGAGGG	CCTCCCTCaaCCTGCCCCCTTAC	13	10
HEK3_Fn	GGCCCAGACTGAGCACGTGATGG	CATCACGTaaGCTCAGTCTGGGC	13	10
EMX1_Fn	TGGTTGCCCACCCTAGTCATTGG	CAATGACTaaAGGGTGGGCAACC	13	10
AAVS1_Fn	TCTAACCCCCACCTCCTGTTAGG	CTAACAGGaaAGGTGGGGGTTAG	13	10
NRAS_Sp	GGTAAGGGGGCAGGGAGGGAGGG	TTCCCTCCaaCTCCCTGCCCCCT	13	10
NRAS_Fn	GGTAAGGGGGCAGGGAGGGAGGG	CCTCCCTCaaCCTGCCCCCTTAC	13	10
c-Myc_Sp	AGGCAGAGGGAGCGAGCGGGCGG	GGCCGCCCaaGCTCGCTCCCTCT	13	10
c-Myc_Fn	AGGCAGAGGGAGCGAGCGGGCGG	CGCCCGCTaaCGCTCCCTCTGCC	13	10
EMX1_Sp_+3	ATTGCCACGAAGCAGGCCAATGG	GTCCTCCCCATaaTGGCCTGCTTCGTGG	13	15
EMX1_Sp_+2	ATTGCCACGAAGCAGGCCAATGG	GTCCTCCCCATTaaGGCCTGCTTCGTGG	13	15
EMX1_Sp_+1	ATTGCCACGAAGCAGGCCAATGG	GTCCTCCCCATTGaaGCCTGCTTCGTGG	13	15
EMX1_Sp_-1	ATTGCCACGAAGCAGGCCAATGG	GTCCTCCCCATTGGaaCCTGCTTCGTGG	13	15

EMX1_Sp_-2	ATTGCCACGAAGCAGGCCAATGG	GTCCTCCCCATTGGCaaCTGCTTCGTGG	13	15
EMX1_Sp_-3	ATTGCCACGAAGCAGGCCAATGG	GTCCTCCCCATTGGCCaaTGCTTCGTGG	13	15
EMX1_Sp_-4	ATTGCCACGAAGCAGGCCAATGG	GTCCTCCCCATTGGCCTaaGCTTCGTGG	13	15
EMX1_Sp_-5	ATTGCCACGAAGCAGGCCAATGG	GTCCTCCCCATTGGCCTGaaCTTCGTGG	13	15
EMX1_Sp_-6	ATTGCCACGAAGCAGGCCAATGG	GTCCTCCCCATTGGCCTGCaaTTCGTGG	13	15
EMX1_Fn_+3	ATTGCCACGAAGCAGGCCAATGG	CTCCCCATaaTGGCCTGCTTCGTGGCAA	13	15
EMX1_Fn_+2	ATTGCCACGAAGCAGGCCAATGG	CTCCCCATTaaGGCCTGCTTCGTGGCAA	13	15
EMX1_Fn_+1	ATTGCCACGAAGCAGGCCAATGG	CTCCCCATTGaaGCCTGCTTCGTGGCAA	13	15
EMX1_Fn_-1	ATTGCCACGAAGCAGGCCAATGG	CTCCCCATTGGaaCCTGCTTCGTGGCAA	13	15
EMX1_Fn_-2	ATTGCCACGAAGCAGGCCAATGG	CTCCCCATTGGCaaCTGCTTCGTGGCAA	13	15
EMX1_Fn_-3	ATTGCCACGAAGCAGGCCAATGG	CTCCCCATTGGCCaaTGCTTCGTGGCAA	13	15
EMX1_Fn_-4	ATTGCCACGAAGCAGGCCAATGG	CTCCCCATTGGCCTaaGCTTCGTGGCAA	13	15
EMX1_Fn_-5	ATTGCCACGAAGCAGGCCAATGG	CTCCCCATTGGCCTGaaCTTCGTGGCAA	13	15
EMX1_Fn_-6	ATTGCCACGAAGCAGGCCAATGG	CTCCCCATTGGCCTGCaaTTCGTGGCAA	13	15
NRAS_-3 G to A sub_Fn_	GGTAAGGGGGCAGGGAGGGAGGG	TCCCTCCCT**T**CCTGCCCCCTTAC	13	10
NRAS_-3 G to T sub_Fn_	GGTAAGGGGGCAGGGAGGGAGGG	TCCCTCCCT**A**CCTGCCCCCTTAC	13	10
NRAS_-2~-1 GG to AT sub_Fn_	GGTAAGGGGGCAGGGAGGGAGGG	TCCCTC**AT**TCCCTGCCCCCTTAC	13	10
NRAS_-3 AA ins_Fn_	GGTAAGGGGGCAGGGAGGGAGGG	CCTCCCTC**TT**CCTGCCCCCTTAC	13	10
NRAS_-3 TT ins_Fn_	GGTAAGGGGGCAGGGAGGGAGGG	CCTCCCTC**AA**CCTGCCCCCTTAC	13	10
NRAS_-3 CC ins_Fn_	GGTAAGGGGGCAGGGAGGGAGGG	CCTCCCTC**GG**CCTGCCCCCTTAC	13	10
NRAS_-3 GG ins_Fn_	GGTAAGGGGGCAGGGAGGGAGGG	CCTCCCTC**CC**CCTGCCCCCTTAC	13	10
Fn_CAR_target 1	CATGCCCTTGACTTGATCCCTGG	AACGCCAGGGATCAaaAGTCAAGGGCAT	13	15
Fn_CAR_target 2	GGACAGAAAAGGGTCAAGGGTGG	TGGGCCACCCTTGaaACCCTTTTCTGTC	13	15
Fn_CAR_target 3	AGATGAGCTGAGGAACTGTGTGG	CATACCACACAGTaaTCCTCAGCTCATC	13	15
Fn_CAR_target 4	GTATGTGGGGACCAAGCCACAGG	GTAGCCTGTGGCTaaTGGTCCCCACATA	13	10
Fn_CAR_target 5	GGTGAGAGTCTCCTCCCCAATGG	ATTTCCATTGGGGAaaGGAGACTCTCAC	13	15
Fn_CAR_target 6	GACAGGCTTGGGCACCAGCGAGG	ACACCCTCGCTGGaaTGCCCAAGCCTGT	13	15
Fn_CAR_target 7	CTTAGAGTCTAACTACCCTGTGG	CCAGCCACAGGGTaaAGTTAGACTCTAA	13	15
Fn_POR_target 1	TACCTGGGACCTCACCCCAAAGG	ATGGCCTTTGGGGaaTGAGGTCCCAGGT	13	15
Fn_POR_target 2	GGGTGAGGCCGGGAAGCGGCAGG	GACCCCTGCCGCTaaTCCCGGCCTCACC	13	15
Fn_POR_target 3	**CATCTCCCCCATGTACACCT**TGG	GCGGCCAAGGTGTaaACATGGGGGAGAT	13	10
Fn_POR_target 4	AAGGTGTACATGGGGGAGATGGG	CCGGCCCATCTCCaaCCCATGTACACCT	13	15
Fn_POR_target 5	TGTACATGGGGGAGATGGGCCGG	TCAGCCGGCCCATaaCTCCCCCATGTAC	13	15
Fn_POR_target 6	AGCCACGCTCCCTCCACTCACGG	GAAGCCGTGAGTGaaGAGGGAGCGTGG C	13	15
Fn_POR_target 7	GTGTATCCCCATATCCCCACAGG	GGGCCCTGTGGGGaaATATGGGGATACA	13	15
Fn_EMX1_target1	TGAAGGTGTGGTTCCAGAACCGG	TCCTCCGGTTCTGaaGAACCACACCTTC	13	15
Fn_EMX1_target2	AGGTGTGGTTCCAGAACCGGAGG	TTGTCCTCCGGTTaaCTGGAACCACACC	13	15
Fn_EMX1_target3	AAAGTACAAACGGCAGAAGCTGG	TCCTCCAGCTTCTaaGCCGTTTGTACTT	13	15
Fn_EMX1_target4	GTACAAACGGCAGAAGCTGGAGG	TCCTCCTCCAGCTaaTCTGCCGTTTGTA	13	15
Fn_EMX1_target5	CAAACGGCAGAAGCTGGAGGAGG	CCTTCCTCCTCCAaaGCTTCTGCCGTTT	13	15
Fn_EMX1_target6	CGGCAGAAGCTGGAGGAGGAAGG	AGGCCCTTCCTCCaaTCCAGCTTCTGCC	13	15
Fn_EMX1_target7	AGGGCTCCCATCACATCAACCGG	GCCACCGGTTGATaaGTGATGGGAGCCC	13	15
Fn_EMX1_target8	GACATCGATGTCCTCCCCATTGG	CAGGCCAATGGGGaaAGGACATCGATGT	13	15

Fn_EMX1_target9	TGGTTGCCCACCCTAGTCATTGG	ACCTCCAATGACTaaAGGGTGGGCAACC	13	10
Fn_EMX1_target10	**ATTGCCACGAAGCAGGCCAA**TGG	CTCCCCATTGGCCaaTGCTTCGTGGCAA	13	15
Fn_Oct-4_target1	GTTTGGCTGAATACCTTCCCTGG	TCCCCCAGGGAAGaaGTATTCAGCCAAA	13	15
Fn_Oct-4_target2	TCTTTTGACTGGCCTCCCCCAGG	CTTCCCTGGGGGAaaGGCCAGTCAAAAG	13	15
Fn_Oct-4_target3	GCCCTTGCTGCAGAAGTGGGTGG	TCCTCCACCCACTaaTCTGCAGCAAGGG	13	15
Fn_VEGFA_target	GGTGAGTGAGTGTGTGCGTGTGG	AACCCCACACGCAaaCACACTCACTCAC	13	15
Fn_AAVS1_target1	TAGCTGAGCTCTCGGACCCCTGG	TCTTCCAGGGGTCaaCGAGAGCTCAGCT	13	15
Fn_AAVS1_target2	AAGATGCCATGACAGGGGGCTGG	TCTTCCAGCCCCCaaTGTCATGGCATCT	13	15
Fn_AAVS1_target3	GGAATCTGCCTAACAGGAGGTGG	ACCCCCACCTCCTaaGTTAGGCAGATTC	13	15
Fn_AAVS1_target4	GGAAGGAGGAGGCCTAAGGATGG	AGCCCCATCCTTAaaGGCCTCCTCCTTC	13	15
Fn_AAVS1_target5	CCCCTGGAAGATGCCATGACAGG	GCCCCCTGTCATGaaGCATCTTCCAGGG	13	15
Fn_AAVS1_target6	CGGGCCCCTATGTCCACTTCAGG	CTGTCCTGAAGTGaaGACATAGGGGCCC	13	15
Fn_AAVS1_target7	AGACCCAATATCAGGAGACTAGG	CCTTCCTAGTCTCaaCTGATATTGGGTC	13	15
Fn_AAVS1_target8	GACAGAAAAGCCCCATCCTTAGG	GAGGCCTAAGGATaaGGGGCTTTTCTGT	13	15
Fn_AAVS1_target9	TCTAACCCCCACCTCCTGTTAGG	TCTGCCTAACAGGaaAGGTGGGGGTTAG	13	15
Fn_AAVS1_target10	AGAACCAGAGCCACATTAACCGG	AGGGCCGGTTAATaaGTGGCTCTGGTTC	13	15
Fn_AAVS1_target11	AAGAAGACTAGCTGAGCTCTCGG	GGGTCCGAGAGCTaaCAGCTAGTCTTCT	13	15
Fn_AAVS1_target12	CTAGTCTTCTTCCTCCAACCCGG	GGGCCCGGGTTGGaaAGGAAGAAGACTA	13	15
Fn_AAVS1_target13	GCTGTCCTGAAGTGGACATAGGG	GGGCCCCTATGTCaaCACTTCAGGACAG	13	15
Fn_HEK3_target2	GGATTGACCCAGGCCAGGGCTGG	TTCTCCAGCCCTGaaGCCTGGGTCAATC	13	10
Fn_HBB_target1	GTAACGGCAGACTTCTCCTCAGG	GACTCCTGAGGAGaaAAGTCTGCCGTTA	13	15
Fn_FANCF_+6 G to C	GGAATCCCTTCTGCAGCACCTGG	AAAGCGATgCAGGTGCTGCAGAAGGGATTC	13	17
Fn_HBB_target2_+4 A to T	CATGGTGCACCTGACTCCTGAGG	CTTCTCCaCAGGAGTCAGGTGCACCAT	13	14
Fn_HEK4_+2 G to T	GGCACTGCGGCTGGAGGTGGGGG	ACCCCaACCTCCAGCCGCA	9	10
Sp_CAR_target1	CATGCCCTTGACTTGATCCCTGG	GATAACGCCAGGGAaaTCAAGTCAAGGG	13	15
Sp_CAR_target2	GGACAGAAAAGGGTCAAGGGTGG	TTTTGGGCCACCCaaTTGACCCTTTTCT	13	15
Sp_CAR_target3	AGATGAGCTGAGGAACTGTGTGG	CCACATACCACACAaaGTTCCTCAGCTC	13	15
Sp_CAR_target4	GTATGTGGGGACCAAGCCACAGG	GTGGTAGCCTGTGaaGCTTGGTCCCCAC	13	10
Sp_CAR_target5	GGTGAGAGTCTCCTCCCCAATGG	GCTATTTCCATTGaaGGGAGGAGACTCT	13	15
Sp_CAR_target6	GACAGGCTTGGGCACCAGCGAGG	TTTACACCCTCGCaaTGGTGCCCAAGCC	13	15
Sp_CAR_target7	CTTAGAGTCTAACTACCCTGTGG	ATGCCAGCCACAGaaGGTAGTTAGACTC	13	15
Sp_POR_target1	TACCTGGGACCTCACCCCAAAGG	TGCATGGCCTTTGaaGGGTGAGGTCCCA	13	15
Sp_POR_target2	GGGTGAGGCCGGGAAGCGGCAGG	ACTGACCCCTGCCaaGCTTCCCGGCCTC	13	15
Sp_POR_target3	CATCTCCCCCATGTACACCTTGG	GATGCGGCCAAGGaaTGTACATGGGGGA	13	10
Sp_POR_target4	AAGGTGTACATGGGGGAGATGGG	CAGCCGGCCCATCaaTCCCCCATGTACA	13	15
Sp_POR_target5	TGTACATGGGGGAGATGGGCCGG	TCTTCAGCCGGCCaaCATCTCCCCCATG	13	15
Sp_POR_target	AGCCACGCTCCCTCCACTCACGG	CCAGAAGCCGTGAaaGTGGAGGGAGCGT	13	15

6				
Sp_POR_target 7	GTGTATCCCCATATCCCCACAGG	GAAGGGCCCTGTGaaGGGATATGGGGAT	13	15
Sp_EMX1_target1	TGAAGGTGTGGTTCCAGAACCGG	TTGTCCTCCGGTTaaCTGGAACCACACC	13	15
Sp_EMX1_target2	AGGTGTGGTTCCAGAACCGGAGG	ACTTTGTCCTCCGaaGTTCTGGAACCAC	13	15
Sp_EMX1_target3	AAAGTACAAACGGCAGAAGCTGG	TCCTCCTCCAGCTaaTCTGCCGTTTGTA	13	15
Sp_EMX1_target4	GTACAAACGGCAGAAGCTGGAGG	CCTTCCTCCTCCAaaGCTTCTGCCGTTT	13	15
Sp_EMX1_target5	CAAACGGCAGAAGCTGGAGGAGG	GGCCCTTCCTCCTaaCCAGCTTCTGCCG	13	15
Sp_EMX1_target6	CGGCAGAAGCTGGAGGAGGAAGG	CTCAGGCCCTTCCaaTCCTCCAGCTTCT	13	15
Sp_EMX1_target7	AGGGCTCCCATCACATCAACCGG	TGCGCCACCGGTTaaGATGTGATGGGAG	13	15
Sp_EMX1_target8	GACATCGATGTCCTCCCCATTGG	AAGCAGGCCAATGaaGGGAGGACATCGA	13	15
Sp_EMX1_target9	TGGTTGCCCACCCTAGTCATTGG	GTCACCTCCAATGaaACTAGGGTGGGCA	13	10
Sp_EMX1_target10	ATTGCCACGAAGCAGGCCAATGG	GTCCTCCCCATTGaaGCCTGCTTCGTGG	13	15
Sp_Oct-4_target1	GTTTGGCTGAATACCTTCCCTGG	GCCTCCCCCAGGGaaAAGGTATTCAGCC	13	15
Sp_Oct-4_target2	TCTTTTGACTGGCCTCCCCCAGG	TACCTTCCCTGGGaaGGAGGCCAGTCAA	13	15
Sp_Oct-4_target3	GCCCTTGCTGCAGAAGTGGGTGG	GCTTCCTCCACCCaaACTTCTGCAGCAA	13	15
Sp_VEGFA_target	GGTGAGTGAGTGTGTGCGTGTGG	CTCAACCCCACACaaGCACACACTCACT	13	15
Sp_AAVS1_target1	TAGCTGAGCTCTCGGACCCCTGG	GCATCTTCCAGGGaaGTCCGAGAGCTCA	13	15
Sp_AAVS1_target2	AAGATGCCATGACAGGGGGCTGG	AGCTCTTCCAGCCaaCCCTGTCATGGCA	13	15
Sp_AAVS1_target3	GGAATCTGCCTAACAGGAGGTGG	CTAACCCCCACCTaaCCTGTTAGGCAGA	13	15
Sp_AAVS1_target4	GGAAGGAGGAGGCCTAAGGATGG	AAAAGCCCCATCCaaTTAGGCCTCCTCC	13	15
Sp_AAVS1_target5	CCCCTGGAAGATGCCATGACAGG	CCAGCCCCCTGTCaaATGGCATCTTCCA	13	15
Sp_AAVS1_target6	CGGGCCCCTATGTCCACTTCAGG	ATGCTGTCCTGAAaaGTGGACATAGGGG	13	15
Sp_AAVS1_target7	AGACCCAATATCAGGAGACTAGG	CCTCCTTCCTAGTaaCTCCTGATATTGG	13	15
Sp_AAVS1_target8	GACAGAAAAGCCCCATCCTTAGG	GAGGAGGCCTAAGaaGATGGGGCTTTTC	13	15
Sp_AAVS1_target9	TCTAACCCCCACCTCCTGTTAGG	GAATCTGCCTAACaaAGGAGGTGGGGGT	13	15
Sp_AAVS1_target10	AGAACCAGAGCCACATTAACCGG	CCCAGGGCCGGTTaaAATGTGGCTCTGG	13	15
Sp_AAVS1_target11	AAGAAGACTAGCTGAGCTCTCGG	CAGGGGTCCGAGAaaGCTCAGCTAGTCT	13	15
Sp_AAVS1_target12	CTAGTCTTCTTCCTCCAACCCGG	TAGGGGCCCGGGTaaTGGAGGAAGAAGA	13	15
Sp_AAVS1_target13	GCTGTCCTGAAGTGGACATAGGG	CCCGGGCCCCTATaaGTCCACTTCAGGA	13	15
Sp_HEK3_target2	GGATTGACCCAGGCCAGGGCTGG	TGCTTCTCCAGCCaaCTGGCCTGGGTCA	13	10
Sp_HBB_target 1	GTAACGGCAGACTTCTCCTCAGG	TCTGACTCCTGAGaaGAGAAGTCTGCCG	13	15
Sp_FANCF_+6 G to C	GGAATCCCTTCTGCAGCACCTGG	GGAAAAGCGATgCAGGTGCTGCAGAAGGGA	13	17
Sp_HBB_target 2_+4 A to T	CATGGTGCACCTGACTCCTGAGG	AGACTTCTCCaCAGGAGTCAGGTGCAC	13	14
Sp_HEK4_+2 G to T	GGCACTGCGGCTGGAGGTGGGGG	TTAACCCCaACCTCCAGCC	9	10
Fn_VEGFA_MP_target1	GGTGAGTGAGTGTGTGCGTGTGG	AACCCCACACGCAaaCACACTCACTCAC	13	15
Sp_VEGFA_MP_target1	CTTCAATATTCCTAGCAAAGAGG	GCCGTTCCCTCTTaaTGCTAGGAATATT	13	15

†The scaffold region of each pegRNA is omitted for simplicity.

Scaffold sequence in pegRNA for SpCas9: GUUUUAGAGCUAGAAAUAGCAAGUUAAAAUAAGGCUAGU CCGUUAUCAACUUGAAAAAGUGGCACCGAGUCGGUGC,

Scaffold sequence in pegRNA for FnCas9: GUUUCAGUUGCUGAAUUAUUUGGUAAACAGUACCAAAUAAUUAA

UGCUCUGUAAUCAUUUAAAAGUAUUUUGAACGGACCUCUGUUUGACACGUCUGAAUAACUAAAAA

Nicking sgRNA	Target sequence (5' to 3')	sgRNA sequence (5' to 3')
HEK3_ngRNA 1 (-108)_Sp	GCAGAAATAGACTAATTGCATGG	GCAGAAAUAGACUAAUUGCA GUUUUAGAGCUAGAAAUAGCAAGUUAAAAUA AGGCUAGUCCGUUAUCAACUUGAAAAAGUG GCACCGAGUCGGUGC
HEK3_ngRNA 2 (-38) _Sp	GGATTGACCCAGGCCAGGGCTGG	GGAUUGACCCAGGCCAGGGC GUUUUAGAGCUAGAAAUAGCAAGUUAAAAUA AGGCUAGUCCGUUAUCAACUUGAAAAAGUG GCACCGAGUCGGUGC
HEK3_ngRNA 3 (+27) _Sp	CGACGCCCTCTGGAGGAAGCAGG	CGACGCCCUCUGGAGGAAGC GUUUUAGAGCUAGAAAUAGCAAGUUAAAAUA AGGCUAGUCCGUUAUCAACUUGAAAAAGUG GCACCGAGUCGGUGC
HEK3_ngRNA 4 (+90) _Sp	GTCAACCAGTATCCCGGTGCAGG	GUCAACCAGUAUCCCGGUGC GUUUUAGAGCUAGAAAUAGCAAGUUAAAAUA AGGCUAGUCCGUUAUCAACUUGAAAAAGUG GCACCGAGUCGGUGC
HEK3_ngRNA 1 (-108)_Fn	GCAGAAATAGACTAATTGCATGG	GCAGAAAUAGACUAAUUGCA GUUUCAGUUGCUGAAUUAUUUGGUAAACAG UACCAAAUAAUUAAUGCUCUGUAAUCAUUUA AAAGUAUUUUGAACGGACCUCUGUUUGACA CGUCUGAAUAACUAAAAA
HEK3_ngRNA 2 (-38) _Fn	GGATTGACCCAGGCCAGGGCTGG	GGAUUGACCCAGGCCAGGGC GUUUCAGUUGCUGAAUUAUUUGGUAAACAG UACCAAAUAAUUAAUGCUCUGUAAUCAUUUA AAAGUAUUUUGAACGGACCUCUGUUUGACA CGUCUGAAUAACUAAAAA
HEK3_ngRNA 3 (+27) _Fn	CGACGCCCTCTGGAGGAAGCAGG	CGACGCCCUCUGGAGGAAGC GUUUCAGUUGCUGAAUUAUUUGGUAAACAG UACCAAAUAAUUAAUGCUCUGUAAUCAUUUA AAAGUAUUUUGAACGGACCUCUGUUUGACA CGUCUGAAUAACUAAAAA

HEK3_ngRNA 4 (+90) _Fn	GTCAACCAGTATCCCGGTGCAGG	GUCAACCAGUAUCCCGGUGC GUUUCAGUUGCUGAAUUAUUUGGUAAACAG UACCAAAUAAUUAAUGCUCUGUAAUCAUUUA AAAGUAUUUUGAACGGACCUCUGUUUGACA CGUCUGAAUAACUAAAAA
EMX1_ngRNA 5 (-80) _Sp	CGTGGGCCCAAGCTGGACTCTGG	CGUGGGCCCAAGCUGGACUC GUUUUAGAGCUAGAAAUAGCAAGUUAAAAUA AGGCUAGUCCGUUAUCAACUUGAAAAAGUG GCACCGAGUCGGUGC
EMX1_ngRNA 6 (-52) _Sp	AGTGCTGCTTGCTGCTGGCCAGG	AGUGCUGCUUGCUGCUGGCC GUUUUAGAGCUAGAAAUAGCAAGUUAAAAUA AGGCUAGUCCGUUAUCAACUUGAAAAAGUG GCACCGAGUCGGUGC
EMX1_ngRNA 7 (+61) _Sp	AGGGCTCCCATCACATCAACCGG	AGGGCUCCCAUCACAUCAAC GUUUUAGAGCUAGAAAUAGCAAGUUAAAAUA AGGCUAGUCCGUUAUCAACUUGAAAAAGUG GCACCGAGUCGGUGC
EMX1_ngRNA 8 (+127) _Sp	ACCGGAGGACAAAGTACAAACGG	ACCGGAGGACAAAGUACAAA GUUUUAGAGCUAGAAAUAGCAAGUUAAAAUA AGGCUAGUCCGUUAUCAACUUGAAAAAGUG GCACCGAGUCGGUGC
EMX1_ngRNA 5 (-80) _Fn	CGTGGGCCCAAGCTGGACTCTGG	CGUGGGCCCAAGCUGGACUC GUUUCAGUUGCUGAAUUAUUUGGUAAACAG UACCAAAUAAUUAAUGCUCUGUAAUCAUUUA AAAGUAUUUUGAACGGACCUCUGUUUGACA CGUCUGAAUAACUAAAAA
EMX1_ngRNA 6 (-52) _Fn	AGTGCTGCTTGCTGCTGGCCAGG	AGUGCUGCUUGCUGCUGGCC GUUUCAGUUGCUGAAUUAUUUGGUAAACAG UACCAAAUAAUUAAUGCUCUGUAAUCAUUUA AAAGUAUUUUGAACGGACCUCUGUUUGACA CGUCUGAAUAACUAAAAA
EMX1_ngRNA 7 (+61) _Fn	AGGGCTCCCATCACATCAACCGG	AGGGCUCCCAUCACAUCAAC GUUUCAGUUGCUGAAUUAUUUGGUAAACAG UACCAAAUAAUUAAUGCUCUGUAAUCAUUUA AAAGUAUUUUGAACGGACCUCUGUUUGACA CGUCUGAAUAACUAAAAA
EMX1_ngRNA 8 (+127) _Fn	ACCGGAGGACAAAGTACAAACGG	ACCGGAGGACAAAGUACAAA GUUUCAGUUGCUGAAUUAUUUGGUAAACAG UACCAAAUAAUUAAUGCUCUGUAAUCAUUUA AAAGUAUUUUGAACGGACCUCUGUUUGACA CGUCUGAAUAACUAAAAA
AAVS1_ngRNA 9 (-84) _Sp	GTCACCAATCCTGTCCCTAGTGG	GUCACCAAUCCUGUCCCUAG GUUUUAGAGCUAGAAAUAGCAAGUUAAAAUA

		AGGCUAGUCCGUUAUCAACUUGAAAAAGUG GCACCGAGUCGGUGC
AAVS1_ngRNA 10 (-52) _Sp	GGAAGGAGGAGGCCTAAGGATGG	GGAAGGAGGAGGCCUAAGGA GUUUUAGAGCUAGAAAUAGCAAGUUAAAAUA AGGCUAGUCCGUUAUCAACUUGAAAAAGUG GCACCGAGUCGGUGC
AAVS1_ngRNA 11 (+38) _Sp	AGAGAGATGGCTCCAGGAAATGG	AGAGAGAUGGCUCCAGGAAA GUUUUAGAGCUAGAAAUAGCAAGUUAAAAUA AGGCUAGUCCGUUAUCAACUUGAAAAAGUG GCACCGAGUCGGUGC
AAVS1_ngRNA 12 (+61) _Sp	AAACCTTAGAGGTTCTGGCAAGG	AAACCUUAGAGGUUCUGGCA GUUUUAGAGCUAGAAAUAGCAAGUUAAAAUA AGGCUAGUCCGUUAUCAACUUGAAAAAGUG GCACCGAGUCGGUGC
AAVS1_ngRNA 13 (+91) _Sp	CTCCCTCCCAGGATCCTCTCTGG	CUCCCUCCCAGGAUCCUCUC GUUUUAGAGCUAGAAAUAGCAAGUUAAAAUA AGGCUAGUCCGUUAUCAACUUGAAAAAGUG GCACCGAGUCGGUGC
AAVS1_ngRNA 9 (-84) _Fn	GTCACCAATCCTGTCCCTAGTGG	GUCACCAAUCCUGUCCCUAG GUUUCAGUUGCUGAAUUAUUUGGUAAACAG UACCAAAUAAUUAAUGCUCUGUAAUCAUUUA AAAGUAUUUUGAACGGACCUCUGUUUGACA CGUCUGAAUAACUAAAAA
AAVS1_ngRNA 10 (-52) _Fn	GGAAGGAGGAGGCCTAAGGATGG	GGAAGGAGGAGGCCUAAGGA GUUUCAGUUGCUGAAUUAUUUGGUAAACAG UACCAAAUAAUUAAUGCUCUGUAAUCAUUUA AAAGUAUUUUGAACGGACCUCUGUUUGACA CGUCUGAAUAACUAAAAA
AAVS1_ngRNA 11 (+38) _Fn	AGAGAGATGGCTCCAGGAAATGG	AGAGAGAUGGCUCCAGGAAA GUUUCAGUUGCUGAAUUAUUUGGUAAACAG UACCAAAUAAUUAAUGCUCUGUAAUCAUUUA AAAGUAUUUUGAACGGACCUCUGUUUGACA CGUCUGAAUAACUAAAAA
AAVS1_ngRNA 12 (+61) _Fn	AAACCTTAGAGGTTCTGGCAAGG	AAACCUUAGAGGUUCUGGCA GUUUCAGUUGCUGAAUUAUUUGGUAAACAG UACCAAAUAAUUAAUGCUCUGUAAUCAUUUA AAAGUAUUUUGAACGGACCUCUGUUUGACA CGUCUGAAUAACUAAAAA
AAVS1_ngRNA 13 (+91) _Fn	CTCCCTCCCAGGATCCTCTCTGG	CUCCCUCCCAGGAUCCUCUC GUUUCAGUUGCUGAAUUAUUUGGUAAACAG UACCAAAUAAUUAAUGCUCUGUAAUCAUUUA AAAGUAUUUUGAACGGACCUCUGUUUGACA CGUCUGAAUAACUAAAAA

NRAS_ngRNA 14 (-144) _Sp	CAATACATGAGGACAGGCGA**AGG**	CAAUACAUGAGGACAGGCGA GUUUUAGAGCUAGAAAUAGCAAGUUAAAAUA AGGCUAGUCCGUUAUCAACUUGAAAAAGUG GCACCGAGUCGGUGC
NRAS_ngRNA 15 (-47) _Sp	GGATTCTTACAGAAAACAAG**TGG**	GGAUUCUUACAGAAAACAAG GUUUUAGAGCUAGAAAUAGCAAGUUAAAAUA AGGCUAGUCCGUUAUCAACUUGAAAAAGUG GCACCGAGUCGGUGC
NRAS_ngRNA 16 (+56) _Sp	GTTAGATGCTTATTTAACCT**TGG**	AGGUUAAAUAAGCAUCUAAC GUUUUAGAGCUAGAAAUAGCAAGUUAAAAUA AGGCUAGUCCGUUAUCAACUUGAAAAAGUG GCACCGAGUCGGUGC
NRAS_ngRNA 14 (-144) _Fn	CAATACATGAGGACAGGCGA**AGG**	CAAUACAUGAGGACAGGCGA GUUUCAGUUGCUGAAUUAUUUGGUAAACAG UACCAAAUAAUUAAUGCUCUGUAAUCAUUUA AAAGUAUUUUGAACGGACCUCUGUUUGACA CGUCUGAAUAACUAAAAA
NRAS_ngRNA 15 (-47) _Fn	GGATTCTTACAGAAAACAAG**TGG**	GGAUUCUUACAGAAAACAAG GUUUCAGUUGCUGAAUUAUUUGGUAAACAG UACCAAAUAAUUAAUGCUCUGUAAUCAUUUA AAAGUAUUUUGAACGGACCUCUGUUUGACA CGUCUGAAUAACUAAAAA
NRAS_ngRNA 16 (+56) _Fn	GTTAGATGCTTATTTAACCT**TGG**	AGGUUAAAUAAGCAUCUAAC GUUUCAGUUGCUGAAUUAUUUGGUAAACAG UACCAAAUAAUUAAUGCUCUGUAAUCAUUUA AAAGUAUUUUGAACGGACCUCUGUUUGACA CGUCUGAAUAACUAAAAA
*c-Myc*_ngRNA 17 (+59) _Sp	CGCTCCGGATCTCCCTTCCC**AGG**	CGCUCCGGAUCUCCCUUCCC GUUUUAGAGCUAGAAAUAGCAAGUUAAAAUA AGGCUAGUCCGUUAUCAACUUGAAAAAGUG GCACCGAGUCGGUGC
*c-Myc*_ngRNA 17 (+59) _Fn	CGCTCCGGATCTCCCTTCCC**AGG**	CGCUCCGGAUCUCCCUUCCC GUUUCAGUUGCUGAAUUAUUUGGUAAACAG UACCAAAUAAUUAAUGCUCUGUAAUCAUUUA AAAGUAUUUUGAACGGACCUCUGUUUGACA CGUCUGAAUAACUAAAAA
*VEGFA*_ngRNA (+55) _Sp	ACACAGATCTATTGGAATCCTGG	CGCUCCGGAUCUCCCUUCCC GUUUUAGAGCUAGAAAUAGCAAGUUAAAAUA AGGCUAGUCCGUUAUCAACUUGAAAAAGUG GCACCGAGUCGGUGC
AAVS1_S_Sp	GGGATCCTGTGTCCCCGAGC**TGG**	GGGATCCTGTGTCCCCGAGCGUUUUAGAGC UAGAAAUAGCAAGUUAAAAUAAGGCUAGUCC

		GUUAUCAACUUGAAAAAGUGGCACCGAGUCGGUGC
AAVS1_AS_Sp	GCTCGGGGACACAGGATCCCTGG	GCUCGGGGACACAGGAUCCCGUUUUAGAGCUAGAAAUAGCAAGUUAAAAUAAGGCUAGUCCGUUAUCAACUUGAAAAAGUGGCACCGAGUCGGUGC
AAVS1_S_Fn	GGGATCCTGTGTCCCCGAGCTGG	GGGAUCCUGUGUCCCCGAGCGUUUCAGUUGCUGAAUUAUUUGGUAAACAGUACCAAAUAAUUAAUGCUCUGUAAUCAUUUAAAAGUAUUUUGAACGGACCUCUGUUUGACACGUCUGAAUAACUAAAAA
AAVS1_AS_Fn	GCTCGGGGACACAGGATCCCTGG	GCUCGGGGACACAGGAUCCCGUUUCAGUUGCUGAAUUAUUUGGUAAACAGUACCAAAUAAUUAAUGCUCUGUAAUCAUUUAAAAGUAUUUUGAACGGACCUCUGUUUGACACGUCUGAAUAACUAAAAA
CAR_S_Sp	TGCTGACCCCTCCTACATTCAGG	TGCTGACCCCTCCTACATTCGUUUUAGAGCUAGAAAUAGCAAGUUAAAAUAAGGCUAGUCCGUUAUCAACUUGAAAAAGUGGCACCGAGUCGGUGC
CAR_AS_Sp	TGTCACAGACTCCTGAATGTAGG	TGTCACAGACTCCTGAATGTGUUUUAGAGCUAGAAAUAGCAAGUUAAAAUAAGGCUAGUCCGUUAUCAACUUGAAAAAGUGGCACCGAGUCGGUGC
CAR_S_Fn	TGCTGACCCCTCCTACATTCAGG	TGCTGACCCCTCCTACATTCGUUUCAGUUGCUGAAUUAUUUGGUAAACAGUACCAAAUAAUUAAUGCUCUGUAAUCAUUUAAAAGUAUUUUGAACGGACCUCUGUUUGACACGUCUGAAUAACUAAAAA
CAR_AS_Fn	TGTCACAGACTCCTGAATGTAGG	TGTCACAGACTCCTGAATGTGUUUCAGUUGCUGAAUUAUUUGGUAAACAGUACCAAAUAAUUAAUGCUCUGUAAUCAUUUAAAAGUAUUUUGAACGGACCUCUGUUUGACACGUCUGAAUAACUAAAAA
EMX1_S_Sp	AAGCAGCACTCTGCCCTCGTGGG	AAGCAGCACTCTGCCCTCGTGUUUUAGAGCUAGAAAUAGCAAGUUAAAAUAAGGCUAGUCCGUUAUCAACUUGAAAAAGUGGCACCGAGUCGGUGC
EMX1_AS_Sp	CATCTGTGCCCCTCCCTCCCTGG	CATCTGTGCCCCTCCCTCCCGUUUUAGAGCUAGAAAUAGCAAGUUAAAAUAAGGCUAGUCCGUUAUCAACUUGAAAAAGUGGCACCGAGUCGGUGC

EMX1_S_Fn	AAGCAGCACTCTGCCCTCGTGGG	AAGCAGCACTCTGCCCTCGTGUUUCAGUUGCUGAAUUAUUUGGUAAACAGUACCAAAUAAUUAAUGCUCUGUAAUCAUUUAAAAGUAUUUUGAACGGACCUCUGUUUGACACGUCUGAAUAACUAAAAA
EMX1_AS_Fn	CATCTGTGCCCCTCCCTCCCTGG	CATCTGTGCCCCTCCCTCCCGUUUCAGUUGCUGAAUUAUUUGGUAAACAGUACCAAAUAAUUAAUGCUCUGUAAUCAUUUAAAAGUAUUUUGAACGGACCUCUGUUUGACACGUCUGAAUAACUAAAAA
FANCF_Sp	GGGGTCCCAGGTGCTGACGTAGG	GGGGTCCCAGGTGCTGACGTGUUUUAGAGCUAGAAAUAGCAAGUUAAAAUAAGGCUAGUCCGUUAUCAACUUGAAAAAGUGGCACCGAGUCGGUGC
FANCF_Fn	GGGGTCCCAGGTGCTGACGTAGG	GGGGTCCCAGGTGCTGACGTGUUUCAGUUGCUGAAUUAUUUGGUAAACAGUACCAAAUAAUUAAUGCUCUGUAAUCAUUUAAAAGUAUUUUGAACGGACCUCUGUUUGACACGUCUGAAUAACUAAAAA
HBB_Sp	AGGGCTGGGCATAAAAGTCAGGG	AGGGCTGGGCATAAAAGTCAGUUUUAGAGCUAGAAAUAGCAAGUUAAAAUAAGGCUAGUCCGUUAUCAACUUGAAAAAGUGGCACCGAGUCGGUGC
HBB_Fn	AGGGCTGGGCATAAAAGTCAGGG	AGGGCTGGGCATAAAAGTCAGUUUCAGUUGCUGAAUUAUUUGGUAAACAGUACCAAAUAAUUAAUGCUCUGUAAUCAUUUAAAAGUAUUUUGAACGGACCUCUGUUUGACACGUCUGAAUAACUAAAAA
HEK4_Sp	TCCCTTCCTTCCACCCAGCCCGG	TCCCTTCCTTCCACCCAGCCGUUUUAGAGCUAGAAAUAGCAAGUUAAAAUAAGGCUAGUCCGUUAUCAACUUGAAAAAGUGGCACCGAGUCGGUGC
HEK4_Fn	TCCCTTCCTTCCACCCAGCCCGG	TCCCTTCCTTCCACCCAGCCGUUUCAGUUGCUGAAUUAUUUGGUAAACAGUACCAAAUAAUUAAUGCUCUGUAAUCAUUUAAAAGUAUUUUGAACGGACCUCUGUUUGACACGUCUGAAUAACUAAAAA
HEK3-4_Sp	AGCCTGGAGACAGGGATCCCAGG	AGCCTGGAGACAGGGATCCCGUUUUAGAGCUAGAAAUAGCAAGUUAAAAUAAGGCUAGUCCGUUAUCAACUUGAAAAAGUGGCACCGAGUCGGUGC
HEK3-4_Fn	AGCCTGGAGACAGGGATCCCAGG	GUUUCAGUUGCUGAAUUAUUUGGUAAACAGUACCAAAUAAUUAAUGCUCUGUAAUCAUUUA

		AAAGUAUUUUGAACGGACCUCUGUUUGACACGUCUGAAUAACUAAAAA
Oct4_S_Sp	CCAAGAAAGGGAAGGTCCCCGGG	CCAAGAAAGGGAAGGUCCCCGUUUUAGAGCUAGAAAUAGCAAGUUAAAAUAAGGCUAGUCCGUUAUCAACUUGAAAAAGUGGCACCGAGUCGGUGC
Oct4_S_Fn	CCAAGAAAGGGAAGGTCCCCGGG	CCAAGAAAGGGAAGGUCCCCGUUUCAGUUGCUGAAUUAUUUGGUAAACAGUACCAAAUAAUUAAUGCUCUGUAAUCAUUUAAAAGUAUUUUGAACGGACCUCUGUUUGACACGUCUGAAUAACUAAAAA
Oct4_AS_Sp	CTTGGATCTCAGGGTCACAAGGG	CUUGGAUCUCAGGGUCACAAGUUUUAGAGCUAGAAAUAGCAAGUUAAAAUAAGGCUAGUCCGUUAUCAACUUGAAAAAGUGGCACCGAGUCGGUGC
Oct4_AS_Fn	CTTGGATCTCAGGGTCACAAGGG	CUUGGAUCUCAGGGUCACAAGUUUCAGUUGCUGAAUUAUUUGGUAAACAGUACCAAAUAAUUAAUGCUCUGUAAUCAUUUAAAAGUAUUUUGAACGGACCUCUGUUUGACACGUCUGAAUAACUAAAAA
POR_S_Sp	AGCCACGCTCCCTCCACTCACGG	AGCCACGCUCCCUCCACUCAGUUUUAGAGCUAGAAAUAGCAAGUUAAAAUAAGGCUAGUCCGUUAUCAACUUGAAAAAGUGGCACCGAGUCGGUGC
POR_S_Fn	AGCCACGCTCCCTCCACTCACGG	AGCCACGCUCCCUCCACUCAGUUUCAGUUGCUGAAUUAUUUGGUAAACAGUACCAAAUAAUUAAUGCUCUGUAAUCAUUUAAAAGUAUUUUGAACGGACCUCUGUUUGACACGUCUGAAUAACUAAAAA
POR_AS_Sp	CATTCGCCAGTACGAGCTTGTGG	CAUUCGCCAGUACGAGCUUGGUUUUAGAGCUAGAAAUAGCAAGUUAAAAUAAGGCUAGUCCGUUAUCAACUUGAAAAAGUGGCACCGAGUCGGUGC
POR_AS_Fn	CATTCGCCAGTACGAGCTTGTGG	CAUUCGCCAGUACGAGCUUGGUUUCAGUUGCUGAAUUAUUUGGUAAACAGUACCAAAUAAUUAAUGCUCUGUAAUCAUUUAAAAGUAUUUUGAACGGACCUCUGUUUGACACGUCUGAAUAACUAAAAA
HBB-1_Sp	CCTTGATACCAACCTGCCCAGGG	CCUUGAUACCAACCUGCCCAGUUUUAGAGCUAGAAAUAGCAAGUUAAAAUAAGGCUAGUCCGUUAUCAACUUGAAAAAGUGGCACCGAGUCGGUGC

HBB-1_Fn	CCTTGATACCAACCTGCCCAGGG	CCTTGATACCAACCTGCCCAGUUUCAGUUGCUGAAUUAUUUGGUAAACAGUACCAAAUAAUUAAUGCUCUGUAAUCAUUUAAAAGUAUUUUGAACGGACCUCUGUUUGACACGUCUGAAUAACUAAAAA
*VEGFA*_ngRNA (+52)_Sp	ACACAGATCTATTGGAATCCTGG	ACACAGATCTATTGGAATCCGUUUUAGAGCUAGAAAUAGCAAGUUAAAAUAAGGCUAGUCCGUUAUCAACUUGAAAAAGUGGCACCGAGUCGGUGC
*VEGFA*_ngRNA (+52)_Fn	ACACAGATCTATTGGAATCCTGG	ACACAGATCTATTGGAATCCGUUUCAGUUGCUGAAUUAUUUGGUAAACAGUACCAAAUAAUUAAUGCUCUGUAAUCAUUUAAAAGUAUUUUGAACGGACCUCUGUUUGACACGUCUGAAUAACUAAAAA

sgRNA	Target sequence (5' to 3')	sgRNA sequence (5' to 3')
EMX1_target11_Sp	TGCGCCACCGGTTGATGTGATGG	CACGAAGCAGGCCAAUGGGG GUUUUAGAGCUAGAAAUAGCAAGUUAAAAUAAGGCUAGUCCGUUAUCAACUUGAAAAAGUGGCACCGAGUCGGUGC
EMX1_target12_Sp	CACGAAGCAGGCCAATGGGGAGG	UGCGCCACCGGUUGAUGUGA GUUUUAGAGCUAGAAAUAGCAAGUUAAAAUAAGGCUAGUCCGUUAUCAACUUGAAAAAGUGGCACCGAGUCGGUGC
EMX1_target11_Fn	TGCGCCACCGGTTGATGTGATGG	CACGAAGCAGGCCAAUGGGG GUUUCAGUUGCUGAAUUAUUUGGUAAACAGUACCAAAUAAUUAAUGCUCUGUAAUCAUUUAAAAGUAUUUUGAACGGACCUCUGUUUGACACGUCUGAAUAACUAAAAA
EMX1_target12_Fn	CACGAAGCAGGCCAATGGGGAGG	UGCGCCACCGGUUGAUGUGA GUUUCAGUUGCUGAAUUAUUUGGUAAACAGUACCAAAUAAUUAAUGCUCUGUAAUCAUUUAAAAGUAUUUUGAACGGACCUCUGUUUGACACGUCUGAAUAACUAAAAA

Table S2. Sequence information for DNA primers used in this study. Primer sequence information to amplify the target gene was indicated. The sequence of the forward and reverse adapter primers used in NGS process is marked in green and blue, respectively.

Target gene (primer direction)	DNA sequence (5′ to 3′)

AAVS1_F1	CCCCTATGTCCACTTCAGGA
AAVS1_R1	ACACCTAGGACGCACCATTC
AAVS1_F2	ATGTGGCTCTGGTTCTGGGT
AAVS1_R2	ACTGACGCACGGAGGAACA
*c-Myc*_F1	AACAGTACTGCTACGGAGGAG
*c-Myc*_R1	AGGAGAGCCTTTCAGAGAAGC
*c-Myc*_F2	TACATCCTAGAGCTAGAGTGCTC
*c-Myc*_R2	GGGTGTTGTAAGTTCCAGTGCA
EMX1_F1	GGGACACTGGGGATCACTAA
EMX1_R1	GAGGAGCTAGGATGCACAG
EMX1_F2	TGCTTGTCCCTCTGTCAATG
EMX1_R2	CCATCCCCTTCTGTGAATGT
HEK3_F1	GAATCAGTGCTGGAGAATGGG
HEK3_R1	AAGCTCCATCACTTTCTGGC
HEK3_F2	CTGCCTAGAAAGGCATGGATG
HEK3_R2	TCTGTTGAGCTCGACCCTGA
NRAS_F1	CTAGTGTGGTAACCTCATTTCCCC
NRAS_R1	ACTTAATCTGTAGCCTCCTGGC
NRAS_F2	CATGTATTGGTCTCTCATGGCA
NRAS_R2	TCCAGGAACACAGTGACCATA
AAVS1_R_F1	CTCCTGTGGATTCGGGTCAC
AAVS1_R_R1	CTGGCTACTGGCCTTATCTCA
Oct4_F1	TGTGTCAGCAGAGCCAGGTG
Oct4_R1	CAGACTATTCCTTGGGGCCAC
HEK4_F1	GTTTACTGCGTGGAGACAGAC
HEK4_R1	GAAATCACCCTGGGGGATCA
HBB-1_F1	CATAGACTCACCCTGAAGTTC
HBB-1_R1	TTAGTGCATCAACTTCTTATTTGTG
HBB-1_F2	TGAGCCAGGCCATCACTAAAG
HBB-1_R2	GACCTCACCCTGTGGAGC
HBB_F	ACCTCACCCTGTGGAGCC
HBB_R	AGAGAGAGTCAGTGCCTATCAG
FANCF_F	AGGTGGTAACGAGCTGCATC
FANCF_R	CGTCACAGTATGTCTCTGGC
AAVS1_R_Adaptor_F1	ACACTCTTTCCCTACACGACGCTCTTCCGATCT<br>TTACCTCTCTAGTCTGTGCTAGCTC

AAVS1_R_ Adaptor_R1	GTGACTGGAGTTCAGACGTGTGCTCTTCCGATCT GGAGGGGACAGATAAAAGTACCC
AAVS1_Fn_ Adaptor_F2	ACACTCTTTCCCTACACGACGCTCTTCCGATCT GGTTCTGGGTACTTTTATCTGTCCC
AAVS1_Fn_ Adaptor_R2	GTGACTGGAGTTCAGACGTGTGCTCTTCCGATCT GTTCTGGCAAGGAGAGAGATGGC
CAR_ Adaptor_F1_R	ACACTCTTTCCCTACACGACGCTCTTCCGATCT TAGCCTAGGTGACAGAGCAAGAAC
CAR_ Adaptor_R1_R	GTGACTGGAGTTCAGACGTGTGCTCTTCCGATCT GCTGTCACAGACTCCTGAATGTAG
CAR_ Adaptor_F2_R	ACACTCTTTCCCTACACGACGCTCTTCCGATCT TGCAAGGGTTTCTTCAGGTGAGAG
CAR_ Adaptor_R2_R	GTGACTGGAGTTCAGACGTGTGCTCTTCCGATCT TATGCCACCAGTTTATACAGTGATGTG
POR_ Adaptor_F2	ACACTCTTTCCCTACACGACGCTCTTCCGATCT ACTCGGGGCACGGGGTCTGG
POR_ Adaptor_R2	GTGACTGGAGTTCAGACGTGTGCTCTTCCGATCT TCCACACCGACATAGATGCGGCC
POR_ Adaptor_F3_R	ACACTCTTTCCCTACACGACGCTCTTCCGATCT GCTGGAAAGGGGAGACCAAGGG
POR_ Adaptor_R3_R	GTGACTGGAGTTCAGACGTGTGCTCTTCCGATCT TCCACGCCGCCTCCCTCC
EMX1_new_ Adaptor_F1	ACACTCTTTCCCTACACGACGCTCTTCCGATCT GGTTGCCCACCCTAGTCATTGGAG
EMX1_new_ Adaptor_R1	GTGACTGGAGTTCAGACGTGTGCTCTTCCGATCT GCCTCCTGAGTTTCTCATCTGTGC
EMX1_new_ Adaptor_F2	ACACTCTTTCCCTACACGACGCTCTTCCGATCT CTGGCCAGCAGCAAGCAGCAC
EMX1_new_ Adaptor_R2	GTGACTGGAGTTCAGACGTGTGCTCTTCCGATCT GAACCGGAGGACAAAGTACAAACGG
Oct4_ Adaptor_F	ACACTCTTTCCCTACACGACGCTCTTCCGATCT CGGGTATCCCCCTCCCACC
Oct4_ Adaptor_R	GTGACTGGAGTTCAGACGTGTGCTCTTCCGATCT GTCACAAGGGCCCTGCGTGC
HEK4_ Adaptor_F	ACACTCTTTCCCTACACGACGCTCTTCCGATCT CTGGGTGGAAGGAAGGGAGGAAG
HEK4_ Adaptor_R	GTGACTGGAGTTCAGACGTGTGCTCTTCCGATCT GAACGGAGACACACACACACAGGCCT
HBB-1_Adaptor_F	ACACTCTTTCCCTACACGACGCTCTTCCGATCT

	CACATGCCCAGTTTCTATTGGTCTCC
HBB-1_Adaptor_R	GTGACTGGAGTTCAGACGTGTGCTCTTCCGATCT TCTATTGCTTACATTTGCTTCTGACACAAC
HBB_Adaptor_F	ACACTCTTTCCCTACACGACGCTCTTCCGATCT AGTCAGGGCAGAGCCATCTATTG
HBB_Adaptor_R	GTGACTGGAGTTCAGACGTGTGCTCTTCCGATCT GTCTCCTTAAACCTGTCTTGTAACC
FANCF_Adaptor_F	ACACTCTTTCCCTACACGACGCTCTTCCGATCT GATGTGGCGCAGGTAGCGCG
FANCF_Adaptor_R	GTGACTGGAGTTCAGACGTGTGCTCTTCCGATCT CCAATCAGTACGCAGAGAGTCGC
*c-Myc*_OT1_F1	TCTGAAGACTGAGAACGGCT
*c-Myc*_OT1_R1	TGCAAATCAACCAATTCAGAGTC
*c-Myc*_OT1_F2	GATCTCTGTGAGGCTAGCAG
*c-Myc*_OT1_R2	AGACTTGCAAGCCAATAGTGTG
*c-Myc*_OT2_F1	TAGGAGGGGTGCTCTGATG
*c-Myc*_OT2_R1	GAAAGCCTACTGCGTGCTAG
*c-Myc*_OT2_F2	CCTGAAGTTACTTGGACAATAG
*c-Myc*_OT2_R2	TAACCGGCGACGACTGAAG
*c-Myc*_OT3_F1	AGTGGAGGTTGCAGTGAGC
*c-Myc*_OT3_R1	AGCTGCCTAGCGATGCATC
*c-Myc*_OT3_F2	GATGACAGAGCAAGAACCTG
*c-Myc*_OT3_R2	ATCCTGGCAAATGCAATGGG
*c-Myc*_OT4_F1	GGAGAAAGCATGCACTGGTG
*c-Myc*_OT4_R1	CAGCTGTTGGAGGGACTTG
*c-Myc*_OT4_F2	CTCTATCTTGAATGGCGCTAC
*c-Myc*_OT4_R2	GTGGGTGTGGGGTAGTAC
*c-Myc*_OT5_F1	TCTATGAAACTGACGAACCATC
*c-Myc*_OT5_R1	CTGAATCAGGGTTACTGTCAC
*c-Myc*_OT5_F2	TACACCCCCAGAGCCAAG
*c-Myc*_OT5_R2	CATGCTCTGCGCGTATTGG
*c-Myc*_OT6_F1	GCACACCCAATACTCCTATCAC
*c-Myc*_OT6_R1	CATCTTGGGAGCTCTGGGAG
*c-Myc*_OT7_F1	TCCACCCTGCTTTCCGTGTG
*c-Myc*_OT7_R1	TCACAAGCCACACTCCCCTC
*c-Myc*_OT8_F1	AATTGGCAGGGTGAGAGCTG
*c-Myc*_OT8_R1	CAACATAGAAGGTCCCCCAC

*c-Myc*_OT9_F1	TTTTAGTAGAGACAGGGTTTCACC
*c-Myc*_OT9_R1	TCTTTATAGCAGTGTTAGAACGGAC
*c-Myc*_OT10_F1	CCTTGTTGTGTGCCTGGATAC
*c-Myc*_OT10_R1	GTGAGGCAGTACGGGGAG
*c-Myc*_OT11_F1	GAATGGCCCTAGACTGGTTAG
*c-Myc*_OT11_R1	CCAGGTGATGCCTATGATCC
*c-Myc*_OT12_F1	ACGCTCATTCACATTTACCGTG
*c-Myc*_OT12_R1	GTTACCTGGTGCAGGGCTG
*c-Myc*_OT13_F1	AAAGTGTGAGAGGAAGAGTTGG
*c-Myc*_OT13_R1	TCAGGATGAGGGTGTCGATG
*c-Myc*_OT14_F1	AGCCGCTCAGCACCTGCG
*c-Myc*_OT14_R1	TTTATCCACTTACACAACTTCAGACG
*c-Myc*_OT15_F1	GTAATTTGATGTGGTTCTGTTTCATG
*c-Myc*_OT15_R1	GAGACTTCTGAGAATACTTACCTG
*c-Myc*_OT16_F1	CTCTTAGGGCAACAGCGATG
*c-Myc*_OT16_R1	AGTCAAGGCCACATCTCACC
NRAS_OT1_F1	ATCTTGGCTCACTGCAACTTC
NRAS_OT1_R1	TGTATCTTCTGCCCACTCAAAG
NRAS_OT1_F2	TTACAGGCCCCTGCTACC
NRAS_OT1_R2	ACAGCCACTGCTCCCTAC
NRAS_OT2_F1	TGTGGGAGATAACTGAATCATG
NRAS_OT2_R1	TGTTCTACCTCATGGCCAAG
NRAS_OT2_F2	GCGAGTAAGTCTCATGACATC
NRAS_OT2_R2	AGCCTTATATGTCGTAGGAAATG
NRAS_OT3_F1	CTGGATGACAGAGTGAGATTC
NRAS_OT3_R1	CCACACCACCTGGTCGTAG
NRAS_OT3_F2	AGCTACTCGGGAGGTTGAG
NRAS_OT3_R2	GATCATGGCGCTTAGCAGAG
NRAS_OT3_F1	ACCCAGACTTGAGTGCAGTG
NRAS_OT4_R1	ATCCTTGCCTACCTCTCCTG
NRAS_OT4_F2	TTCAAGCAATCCTCCCACTTC
NRAS_OT4_R2	TAGGCTAGTTCCCTGATAGATG
NRAS_OT5_F1	TGGAAAGATCCCTGTGGTGG
NRAS_OT5_R1	ACTGAGGCACACAACACACG
NRAS_OT5_F2	AGGTGGTGTTCTGAAGGGAG
NRAS_OT5_R2	CTCATGCACTCCCAGCAATG

NRAS_OT6_F1	GCCATCAGCACCTTGGGAG
NRAS_OT6_R1	GCTCCAACAGGAACCCAATAC
NRAS_OT6_F2	TTGCTGCAGAAGATGGCGGA
NRAS_OT6_R2	CCAGCCTTGGAGTGCAACG
NRAS_OT7_F	AGGAACACAGGAAGGAAGG
NRAS_OT7_R	AAGTCCTAGCATGGCTCTTGG
NRAS_OT8_F	GACTGAGCCAACTCTTCACG
NRAS_OT8_R	GTCGCAGAATGAGGAGTGG
NRAS_OT9_F	TCACTCCTTAGCATGCACCC
NRAS_OT9_R	CATCCCTCTGTCCCTCTATC
NRAS_OT10_F	TCCGAAGTGTACAGTGGCAC
NRAS_OT10_R	ATTACACACGCACACGTTCATTGC
NRAS_OT11_F	TTTGACTTATTTGCTGCCGCC
NRAS_OT11_R	TGAATCTTGGGGTGAGATTACCTATC
NRAS_OT12_F	TGTAATCCCTGCACTTTGGGAG
NRAS_OT12_R	CTCTAGTGTTGGGTTGCAAGTACG
NRAS_OT13_F	CACAATCTCCCACCTTTCTGGATAG
NRAS_OT13_R	CAGGAGAATGGCGTGAACC
NRAS_OT14_F	TGGGAATGAATGGCAGAGAAATGC
NRAS_OT14_R	GCGATTCTCCTGCCTCAGTC
NRAS_OT15_F	ATGTGCCATGGTGGTTTGC
NRAS_OT15_R	GATCGCGAGGTCAGGAAATC
NRAS_OT16_F	GGTTAACTCACTGACTTAGCAACTCC
NRAS_OT16_R	CTGAACTGCTTCCAGAGCC
*HEK3-4*_OT1_F	ACGGACAAGACACCTATGTAATG
*HEK3-4*_OT1_R	CCTCTGAAGAGGCGCTTGG
*HEK3-4*_OT2_F	TACGAAGGAGGAAACCCAGG
*HEK3-4*_OT2_R	CACTTAGCTCCCGTGGCAC
*HEK3-4*_OT3_F	TCTCTGTGCCTGGACTGCAC
*HEK3-4*_OT3_R	CTATGCGACACTACTTCTTAAACAG
*HEK3-4*_OT4_F	TGAACAACCATGCTTTAGGCTC
*HEK3-4*_OT4_R	AGCTCCTGGGTTATAGCCTC
*HEK3-4*_OT5_F	TGAGCGTCCAGAATTCCTGAG
*HEK3-4*_OT5_R	GGCTGAGGCAGGAGAATCAC
*HEK3-4*_OT6_F	CTTTCTCCAGTAGCCTGCTG
*HEK3-4*_OT6_R	TGGAGCTGGAAGCGTCCTG
*HEK3-4*_OT7_F	CATGAGGTCAGGAGTTTGAGATC
*HEK3-4*_OT7_R	GTTGATCTCTCACAGGCCTAG
*HEK3-4*_OT8_F	GTCCCACAGACACTGCTGC
*HEK3-4*_OT8_R	AGGGTGTGTGGTAAGGACGG

*HEK3-4*_OT9_F	GGTGGGGAGAGGAAGTCTTC
*HEK3-4*_OT9_R	CTAATTGAGAATTTCAGGAAGTGTAG
*HEK3-4*_OT10_F	GCGTCGGCAGTCTTTGCTG
*HEK3-4*_OT10_R	CCAGGAGCAGCTCTGCATG
*HEK3-4*_OT11_F	AGGAACTGCATCACTCGGACG
*HEK3-4*_OT11_R	GCAGGAACAGCCCTCTGAAG
*HEK3-4*_OT12_F	AAGACACCTATGTAATGACCATAG
*HEK3-4*_OT12_R	CGCAGGAACAGCCCTCTG
*HEK3-4*_OT13_F	GGAATGAGGAGTGTAGTGGAG
*HEK3-4*_OT13_R	ATAACTGGGATATCTTACATCTGC
*HEK3-4*_OT14_F	TGAAGGCACCACAGTCACAAG
*HEK3-4*_OT14_R	CCTGAGAGAAGCTGAGGCC
*HEK3-4*_OT15_F	TTTCCAAGCACCTCCAAGGAG
*HEK3-4*_OT15_R	GAACTCCTGGGCTCAAGCAG
*HEK3-4*_OT16_F	TCATAAAAGCTGCTGTGTGATAAC
*HEK3-4*_OT16_R	TGGTGACTGGGGACATGCC
*FANCF*_OT1_F	CTTAGCCTACCAAGTAGCTGG
*FANCF*_OT1_R	TACCACCTCTCCGGGACC
*FANCF*_OT2_F	GTCGTAGGAGTTGCCCAGG
*FANCF*_OT2_R	CCAGCTTTCATCTGCCCTTTCC
*FANCF*_OT3_F	TACAAAGAGGCAATTAGAACACAG
*FANCF*_OT3_R	ACGGACCAGCCACGATGG
*FANCF*_OT4_F	GTGGTCATTCCAGTAAATCTACAC
*FANCF*_OT4_R	TTGATGAAGGTGGCTAGATTAAAC
*FANCF*_OT5_F	CGCCATTCTCCTGCCTCAG
*FANCF*_OT5_R	GTGAATAATGCTGCTATGAAGATC
*FANCF*_OT6_F	ACTGTTACGTAGTGTTTGTGCAC
*FANCF*_OT6_R	ATCCCAGGTCTTCTACTGAAATTG
*FANCF*_OT7_F	ATCTCTCACCAACGGACTACC
*FANCF*_OT7_R	ACAAGGATGCCCACTACCTC
*FANCF*_OT8_F	CCACTACAGAGCTTCTTCAGG
*FANCF*_OT8_R	CTAAGGTGTATGAGCATTGTGAC
*FANCF*_OT9_F	TCTGTCTCTTGCCTGTTGTCG
*FANCF*_OT9_R	TCTGGTGTCTGAGTTTGCAGC
*FANCF*_OT10_F	TCCTTGCCTGACAGAAGTTCC
*FANCF*_OT10_R	CAAGGCCTGTCTTTAGTCCAC

*FANCF*_OT11_F	GAAGTTGGTAAGCCCTCCAG
*FANCF*_OT11_R	GGGGAAATGAGTAGAACGTGAG
*FANCF*_OT12_F	ACTTCTAACCTGGATAATTTGCTG
*FANCF*_OT12_R	TCAAGTGTGACTGTACCAGAAG
*FANCF*_OT13_F	CAGGGCCTTGAGGACTCTC
*FANCF*_OT13_R	AGGAGTTCGAGACCAGCCTG
*FANCF*_OT14_F	TTCCGCTCCCAGGACAGTG
*FANCF*_OT14_R	TGGCTTCCTCTCGATGTCACAAAC
*FANCF*_OT15_F	TGTGCTTCCCTGATCATGGC
*FANCF*_OT15_R	GATTTATGTACTTTTCTGTCTGCAG
*FANCF*_OT16_F	CACACTTCTCTGCCAGCTCC
*FANCF*_OT16_R	CTCTCCATTGATCAACCAACAAG
*HBB*_OT1_F	ACCTAGCACCTTCTTGCCATG
*HBB*_OT1_R	TGCTGAAAGAGATGCGGTGG
*HBB*_OT2_F	AGATCGTCATGGAGACGCTG
*HBB*_OT2_R	GCTTCTAGGGTGTGAGACATC
*HBB*_OT3_F	CCCATGGAAGCCTCCTGG
*HBB*_OT3_R	GTCACCAGGGAGTTCTGTTATC
*HBB*_OT4_F	CAGCTGGATTCTCTTCCCTG
*HBB*_OT4_R	GGTGATGTGAGCCTGTAGTC
*HBB*_OT5_F	TGTCTCCTCCCTTGTCACTG
*HBB*_OT5_R	CTTGCTGGGTTACATGGCAG
*HBB*_OT6_F	TGGAGATCCTGCCAGAGTTG
*HBB*_OT6_R	GCAGGGAGACCAATACAGTAG
*HBB*_OT7_F	CGTCTGACCACAGGCCTG
*HBB*_OT7_R	CTTGAACTCAGGAGGTGGAG
*HBB*_OT8_F	ATAGAGAGCAGCCAAGTGGC
*HBB*_OT8_R	AGAGCAAGAGACGTAATGTGAG
*HBB*_OT9_F	CTTAGGTCAGGAGTTCGAGAC
*HBB*_OT9_R	TGGTTGTGAGGACCAAGTGC
*HBB*_OT10_F	CTGGAAAGCGACGGTGGTG
*HBB*_OT10_R	AGTCTTGCTCTGTCACCCAG
*HBB*_OT11_F	GCTTTCCTCTCAGGGCTACG
*HBB*_OT11_R	GGAGGACTGAGGGGTAACG
*HBB*_OT12_F	CATTGTCATCTCACTGAAACCTC
*HBB*_OT12_R	GAGAGAACTGGCAACTGGTG

*HBB*_OT13_F	TAGTGTACTGCGGTAGGAGTG
*HBB*_OT13_R	TTGGCGTAGCTTCCCTCCG
*HBB*_OT14_F	TCAGGTTGACTTTAGAGAGTTCC
*HBB*_OT14_R	TTGAGTGGGTGTTGGGAGTTC
*HBB*_OT15_F	TGGAGGCAGGGATGCTGAG
*HBB*_OT15_R	GGGAAGGCATGGTCTTGTAG
*HBB*_OT16_F	ACTGAATCCCAGAAAATGGTTGAG
*HBB*_OT16_R	CTCTCCCCTCCCATCACC
AAVS1_Adaptor_F	ACACTCTTTCCCTACACGACGCTCTTCCGATCT<br>CTAGGGACAGGATTGGTGACAG
AAVS1_Adaptor_R	GTGACTGGAGTTCAGACGTGTGCTCTTCCGATCT<br>TGCCAAGCTCTCCCTCCCAG
*c-Myc*_Adaptor_F	ACACTCTTTCCCTACACGACGCTCTTCCGATCT<br>GAGGGAGGGATCGCGCTGAGTA
*c-Myc*_Adaptor_R	GTGACTGGAGTTCAGACGTGTGCTCTTCCGATCT<br>GAGGGCTGGGCCAGAGGCGAA
EMX1_Adaptor_F1	ACACTCTTTCCCTACACGACGCTCTTCCGATCT<br>GAGTGGCCAGAGTCCAGCTTG
EMX1_Adaptor_R1	GTGACTGGAGTTCAGACGTGTGCTCTTCCGATCT<br>AGGAGGAAGGGCCTGAGTCC
EMX1_Adaptor_F2	ACACTCTTTCCCTACACGACGCTCTTCCGATCT<br>CGGAGGACAAAGTACAAACGGCCGA
EMX1_Adaptor_R2	GTGACTGGAGTTCAGACGTGTGCTCTTCCGATCT<br>TGGCCAGCAGCAAGCAGCACTCT
HEK3_Adaptor_F1	ACACTCTTTCCCTACACGACGCTCTTCCGATCT<br>GCATTTGTAGGCTTGATGCTTT
HEK3_Adaptor_R	GTGACTGGAGTTCAGACGTGTGCTCTTCCGATCT<br>CCCAGCCAAACTTGTCAACC
NRAS__Adaptor_F1	ACACTCTTTCCCTACACGACGCTCTTCCGATCT<br>GTCCAGCTGTATCCAGTATGTCCAA
NRAS__Adaptor_R1	GTGACTGGAGTTCAGACGTGTGCTCTTCCGATCT<br>CAGATAGGCAGAAATGGGCTTGAATA
NRAS__Adaptor_F2	ACACTCTTTCCCTACACGACGCTCTTCCGATCT<br>TCTCATGGCACTGTACTCTTCTTGTC
NRAS__Adaptor_R2	GTGACTGGAGTTCAGACGTGTGCTCTTCCGATCT<br>GATGCTTATTTAACCTTGGCAATAGC
*c-Myc*_OT1__Adaptor_F	ACACTCTTTCCCTACACGACGCTCTTCCGATCT<br>GAGACACAGACACAGACACAACTGC

*c-Myc*_OT1_Adaptor_R	GTGACTGGAGTTCAGACGTGTGCTCTTCCGATCT AGCATGTCCACCCCACCTGGC
*c-Myc*_OT2__Adaptor_F	ACACTCTTTCCCTACACGACGCTCTTCCGATCT CGAAGGTTGCTGCCTCCCTCG
*c-Myc*_OT2_Adaptor_R	GTGACTGGAGTTCAGACGTGTGCTCTTCCGATCT GTCCTTATTGGGCAAAGCCGAGATG
*c-Myc*_OT3__Adaptor_F	ACACTCTTTCCCTACACGACGCTCTTCCGATCT CAAATGAAGTGGCAGTAGCAGGAC
*c-Myc*_OT3_Adaptor_R	GTGACTGGAGTTCAGACGTGTGCTCTTCCGATCT TAAATGTCTTCAAGCCCTCCCAGG
*c-Myc*_OT4__Adaptor_F	ACACTCTTTCCCTACACGACGCTCTTCCGATCT TTCATTCAGGCGGGTCCGGCC
*c-Myc*_OT4_Adaptor_R	GTGACTGGAGTTCAGACGTGTGCTCTTCCGATCT TGGAGGAGACATCAAAGCCCGAATG
*c-Myc*_OT5__Adaptor_F	ACACTCTTTCCCTACACGACGCTCTTCCGATCT CAGACCCGCGCCCCAGAAAC
*c-Myc*_OT5_Adaptor_R	GTGACTGGAGTTCAGACGTGTGCTCTTCCGATCT CCGCTCTGCTCTGTCCGGTC
*c-Myc*_OT6_Adaptor_F	ACACTCTTTCCCTACACGACGCTCTTCCGATCT GGTGCTGTGGTGGTTTATGGATGAAG
*c-Myc*_OT6_Adaptor_R	GTGACTGGAGTTCAGACGTGTGCTCTTCCGATCT AAATTGTGGATATGCCCAAAGCCCTG
*c-Myc*_OT7_Adaptor_F	ACACTCTTTCCCTACACGACGCTCTTCCGATCT CTTCCTCGGGGTCGCGGGAG
*c-Myc*_OT7_Adaptor_R	GTGACTGGAGTTCAGACGTGTGCTCTTCCGATCT GCAGCTTCCAGCAGTCGCCTC
*c-Myc*_OT8_Adaptor_F	ACACTCTTTCCCTACACGACGCTCTTCCGATCT GCAGTTGCTGTTCGACCCCCAGC
*c-Myc*_OT8_Adaptor_R	GTGACTGGAGTTCAGACGTGTGCTCTTCCGATCT CTTTTATTGGAGAAAAGGACAGCATCC
*c-Myc*_OT9_Adaptor_F	ACACTCTTTCCCTACACGACGCTCTTCCGATCT CACCTGAACACTTAGAGGCTATTGTAAG
*c-Myc*_OT9_Adaptor_R	GTGACTGGAGTTCAGACGTGTGCTCTTCCGATCT GATCTGTGATGAGCAATGTTTGATGGTAC
*c-Myc*_OT10_Adaptor_F	ACACTCTTTCCCTACACGACGCTCTTCCGATCT TGTGCTTGTGTGTGTGCGCCGG
*c-Myc*_OT10_Adaptor_R	GTGACTGGAGTTCAGACGTGTGCTCTTCCGATCT CGCGCGTCTACTCCATGCCG
*c-Myc*_OT11_Adaptor_F	ACACTCTTTCCCTACACGACGCTCTTCCGATCT

	AAGGGTTGTAGCAGCCCAGATTCAC
*c-Myc*_OT11_Adaptor_R	GTGACTGGAGTTCAGACGTGTGCTCTTCCGATCT<br>CTTCAGCAATTGGCCTTCACCGCC
*c-Myc*_OT12_Adaptor_F	ACACTCTTTCCCTACACGACGCTCTTCCGATCT<br>TACGGCCACCAAGTGCACTTAGG
*c-Myc*_OT12_Adaptor_R	GTGACTGGAGTTCAGACGTGTGCTCTTCCGATCT<br>CTCGAGTTGCCGCTGCCGC
*c-Myc*_OT13_Adaptor_F	ACACTCTTTCCCTACACGACGCTCTTCCGATCT<br>CGAGATCAGGCCAGGGGAGAG
*c-Myc*_OT13_Adaptor_R	GTGACTGGAGTTCAGACGTGTGCTCTTCCGATCT<br>GAGCGCCCGCGGCTCCATG
*c-Myc*_OT14_Adaptor_F	ACACTCTTTCCCTACACGACGCTCTTCCGATCT<br>CGACATCTAGAAGAGTGGCCCTC
*c-Myc*_OT14_Adaptor_R	GTGACTGGAGTTCAGACGTGTGCTCTTCCGATCT<br>CTCTGGACCCTCAGCTGTCACC
*c-Myc*_OT15_Adaptor_F	ACACTCTTTCCCTACACGACGCTCTTCCGATCT<br>TGATTAATTTCCTATTCACACACTCAGGC
*c-Myc*_OT15_Adaptor_R	GTGACTGGAGTTCAGACGTGTGCTCTTCCGATCT<br>AGGGTGAGATTATATACCCTGTTTAAGG
*c-Myc*_OT16_Adaptor_F	ACACTCTTTCCCTACACGACGCTCTTCCGATCT<br>GCTAACACTGGGGACAGCCAGG
*c-Myc*_OT16_Adaptor_R	GTGACTGGAGTTCAGACGTGTGCTCTTCCGATCT<br>TGGACGGCCCCACCCCAGAG
NRAS_OT1__Adaptor_F	ACACTCTTTCCCTACACGACGCTCTTCCGATCT<br>GGACATTAAGGAAGTAGCCAAGTGAC
NRAS_OT1_Adaptor_R	GTGACTGGAGTTCAGACGTGTGCTCTTCCGATCT<br>CTCCCTTCCTCCCTTTTCTTTCTTTC
NRAS_OT2__Adaptor_F	ACACTCTTTCCCTACACGACGCTCTTCCGATCT<br>ACTTCTCTCTCTCCTTCCCCCCTG
NRAS_OT2_Adaptor_R	GTGACTGGAGTTCAGACGTGTGCTCTTCCGATCT<br>GTGAGGAGGGAGGGAGGGGAG
NRAS_OT3__Adaptor_F	ACACTCTTTCCCTACACGACGCTCTTCCGATCT<br>CTGGGTGACAGAATGAGTGTCTG
NRAS_OT3_Adaptor_R	GTGACTGGAGTTCAGACGTGTGCTCTTCCGATCT<br>AGCTCTATGGACTTCAGCTTCCTC
NRAS_OT4__Adaptor_F	ACACTCTTTCCCTACACGACGCTCTTCCGATCT<br>GTAAATCTGAGAATACTAATCCACAAACTAATTAC
NRAS_OT4_Adaptor_R	GTGACTGGAGTTCAGACGTGTGCTCTTCCGATCT<br>ATTGTGGGAGGGAGAGAGGAAGAG

NRAS_OT5__Adaptor_F	ACACTCTTTCCCTACACGACGCTCTTCCGATCT TTTGAGAGTCCTTACCACATAAGATGG
NRAS_OT5_Adaptor_R	GTGACTGGAGTTCAGACGTGTGCTCTTCCGATCT CTGGCCTCATCTTCACCTCTCAG
NRAS_OT6__Adaptor_F	ACACTCTTTCCCTACACGACGCTCTTCCGATCT GTCTAGTATGTGGCTGCCTGTTGTC
NRAS_OT6_Adaptor_R	GTGACTGGAGTTCAGACGTGTGCTCTTCCGATCT GCCCCTTCTTCACTCAGAACCTC
NRAS_OT7_Adaptor_F	ACACTCTTTCCCTACACGACGCTCTTCCGATCT CAAGCAAGCAAGAAAGAACAAAGAAAGAAAGAGAGAAAG
NRAS_OT7_Adaptor_R	GTGACTGGAGTTCAGACGTGTGCTCTTCCGATCT TCATTCATTCCTTCCTTCCCTCTCTC
NRAS_OT8_Adaptor_F	ACACTCTTTCCCTACACGACGCTCTTCCGATCT GGACACACCCATGAAACCATCACCAC
NRAS_OT8_Adaptor_R	GTGACTGGAGTTCAGACGTGTGCTCTTCCGATCT CATACTCCATTTTCTAAGGTGGGGAGGTGTG
NRAS_OT9_Adaptor_F	ACACTCTTTCCCTACACGACGCTCTTCCGATCT TGTCTGACTCCCTTCCAAGTGCTGAG
NRAS_OT9_Adaptor_R	GTGACTGGAGTTCAGACGTGTGCTCTTCCGATCT CATCCACCCTTCATCCATTCATCCCTCAATTC
NRAS_OT10_Adaptor_F	ACACTCTTTCCCTACACGACGCTCTTCCGATCT CTGCCCGCTGCTGACACACAC
NRAS_OT10_Adaptor_R	GTGACTGGAGTTCAGACGTGTGCTCTTCCGATCT TGGAAGAGAAGGGCTATGAAGTGGGG
NRAS_OT11_Adaptor_F	ACACTCTTTCCCTACACGACGCTCTTCCGATCT TCTCTTTGAGGCGCGGTGCTTTG
NRAS_OT11_Adaptor_R	GTGACTGGAGTTCAGACGTGTGCTCTTCCGATCT TGCCTCTAGCAGAATTTGACATAGCTTCAGCC
NRAS_OT12_Adaptor_F	ACACTCTTTCCCTACACGACGCTCTTCCGATCT CCTGGGTGACAAGAGTGAAACTCC
NRAS_OT12_Adaptor_R	GTGACTGGAGTTCAGACGTGTGCTCTTCCGATCT CTTCCTATACACAAATTGCCTTTACCCATGTGTGTC
NRAS_OT13_Adaptor_F	ACACTCTTTCCCTACACGACGCTCTTCCGATCT CCTTCCTGCATCCTCTCGG
NRAS_OT13_Adaptor_R	GTGACTGGAGTTCAGACGTGTGCTCTTCCGATCT ACATGAATGAAGATTTGCGGGGCATGGATAATAC
NRAS_OT14_Adaptor_F	ACACTCTTTCCCTACACGACGCTCTTCCGATCT GAAGGTAGAGCCCAATGAGGATGATTAGG
NRAS_OT14_Adaptor_R	GTGACTGGAGTTCAGACGTGTGCTCTTCCGATCT GGCCTTCTTTCCAAGTCATCCCATG
NRAS_OT15_Adaptor_F	ACACTCTTTCCCTACACGACGCTCTTCCGATCT CACCATTGCACTCCAGCCTGG
NRAS_OT15_Adaptor_R	GTGACTGGAGTTCAGACGTGTGCTCTTCCGATCT CCGTTGTCCTCAGCAAACTAACACAG

NRAS_OT16_Adaptor_F	ACACTCTTTCCCTACACGACGCTCTTCCGATCT GGATATCTTTCTGGCTTCTACCCAAGACAACTTC
NRAS_OT16_Adaptor_R	GTGACTGGAGTTCAGACGTGTGCTCTTCCGATCT AGGAAATTTTCTTCCTTCTTTGCTTCCTTCCTTCC
*HEK3-4*_OT1_Adaptor_F	ACACTCTTTCCCTACACGACGCTCTTCCGATCT GAGTGCCCACAACAAGCCAATTTC
*HEK3-4*_OT1_Adaptor_R	GTGACTGGAGTTCAGACGTGTGCTCTTCCGATCT CCGCTGTCCTCTTGTAGTGCAG
*HEK3-4*_OT2_Adaptor_F	ACACTCTTTCCCTACACGACGCTCTTCCGATCT TTATCTCCCAGGATTCTAGGCTGG
*HEK3-4*_OT2_Adaptor_R	GTGACTGGAGTTCAGACGTGTGCTCTTCCGATCT TCTGAGCAGATGGCTGGGTGTG
*HEK3-4*_OT3_Adaptor_F	ACACTCTTTCCCTACACGACGCTCTTCCGATCT AGCAGTGGTGGCTGGCCTTATC
*HEK3-4*_OT3_Adaptor_R	GTGACTGGAGTTCAGACGTGTGCTCTTCCGATCT GCTCCCGTGGCCACTGCAG
*HEK3-4*_OT4_Adaptor_F	ACACTCTTTCCCTACACGACGCTCTTCCGATCT CAGAGCAAGTTCAGAGGTAGAGAATC
*HEK3-4*_OT4_Adaptor_R	GTGACTGGAGTTCAGACGTGTGCTCTTCCGATCT CCTTCCTCCAGGGGCAGCTTC
*HEK3-4*_OT5_Adaptor_F	ACACTCTTTCCCTACACGACGCTCTTCCGATCT TGTTTAGCCGGTGGCTGACTGAC
*HEK3-4*_OT5_Adaptor_R	GTGACTGGAGTTCAGACGTGTGCTCTTCCGATCT ACCTCAGACAGAATCAGGGAAGAC
*HEK3-4*_OT6_Adaptor_F	ACACTCTTTCCCTACACGACGCTCTTCCGATCT GATTCAAGGCAAGAGCAGGGGAC
*HEK3-4*_OT6_Adaptor_R	GTGACTGGAGTTCAGACGTGTGCTCTTCCGATCT ATGAAGTCCTGGAGTTCACAGAGACC
*HEK3-4*_OT7_Adaptor_F	ACACTCTTTCCCTACACGACGCTCTTCCGATCT GCAAACATCTTACATATCCCTAACCAGG
*HEK3-4*_OT7_Adaptor_R	GTGACTGGAGTTCAGACGTGTGCTCTTCCGATCT ACAGATGCTGTGTCCTGTGCAAGAC
*HEK3-4*_OT8_Adaptor_F	ACACTCTTTCCCTACACGACGCTCTTCCGATCT GAGTCACCCACACGCTTCTTTGAT
*HEK3-4*_OT8_Adaptor_R	GTGACTGGAGTTCAGACGTGTGCTCTTCCGATCT GGGAGCAGATGTCCTGTCCAC
*HEK3-4*_OT9_Adaptor_F	ACACTCTTTCCCTACACGACGCTCTTCCGATCT TTAGCTAACGTCACCTGCATTTTCCC
*HEK3-4*_OT9_Adaptor_R	GTGACTGGAGTTCAGACGTGTGCTCTTCCGATCT CCATTCCTATGGAAACCAATACCAAGG

*HEK3-4*_OT10_Adaptor_F	ACACTCTTTCCCTACACGACGCTCTTCCGATCT AGTTCCTCTGTGTGGTGTCCAAGG
*HEK3-4*_OT10_Adaptor_R	GTGACTGGAGTTCAGACGTGTGCTCTTCCGATCT CAAGAAGTCAGCAAGGCCACCAGG
*HEK3-4*_OT11_Adaptor_F	ACACTCTTTCCCTACACGACGCTCTTCCGATCT AAGAGTGCCCACAACAAGCCAATTTC
*HEK3-4*_OT11_Adaptor_R	GTGACTGGAGTTCAGACGTGTGCTCTTCCGATCT CTGTCCTCTTGTAGTGCAGGGATG
*HEK3-4*_OT12_Adaptor_F	ACACTCTTTCCCTACACGACGCTCTTCCGATCT AGATGAAAAGAGTGCCCACAACAAGC
*HEK3-4*_OT12_Adaptor_R	GTGACTGGAGTTCAGACGTGTGCTCTTCCGATCT TCTTGTAGTGCAGGGATGGGAGGG
*HEK3-4*_OT13_Adaptor_F	ACACTCTTTCCCTACACGACGCTCTTCCGATCT CTGAGGATTCTTGAGCAGGTGATTTATTG
*HEK3-4*_OT13_Adaptor_R	GTGACTGGAGTTCAGACGTGTGCTCTTCCGATCT ATCTGTCCTGCTTACCCCAACGG
*HEK3-4*_OT14_Adaptor_F	ACACTCTTTCCCTACACGACGCTCTTCCGATCT ACACACGGCTGTTTCCGGGAAG
*HEK3-4*_OT14_Adaptor_R	GTGACTGGAGTTCAGACGTGTGCTCTTCCGATCT AAAGGTATCTCCCGGACCAGCAC
*HEK3-4*_OT15_Adaptor_F	ACACTCTTTCCCTACACGACGCTCTTCCGATCT ATCCTAATAATGGTCATCAGATCACTCAG
*HEK3-4*_OT15_Adaptor_R	GTGACTGGAGTTCAGACGTGTGCTCTTCCGATCT CAGAGGCTTAGAAGATGAGGCCATTC
*HEK3-4*_OT16_Adaptor_F	ACACTCTTTCCCTACACGACGCTCTTCCGATCT GCCGTACCTTGAGGACTGGCC
*HEK3-4*_OT16_Adaptor_R	GTGACTGGAGTTCAGACGTGTGCTCTTCCGATCT GCCATTCCCTGTGGCTGGTTAAG
*FANCF*_OT1_Adaptor_F	ACACTCTTTCCCTACACGACGCTCTTCCGATCT CAAGCGCATCACAGCGAGGAAG
*FANCF*_OT1_Adaptor_R	GTGACTGGAGTTCAGACGTGTGCTCTTCCGATCT GGTTGGATCAGCTCTGTCGTCC
*FANCF*_OT2_Adaptor_F	ACACTCTTTCCCTACACGACGCTCTTCCGATCT GGCACGGACGCAGTATTCTATC
*FANCF*_OT2_Adaptor_R	GTGACTGGAGTTCAGACGTGTGCTCTTCCGATCT CCAGCTTTCATCTGCCCTTTCC
*FANCF*_OT3_Adaptor_F	ACACTCTTTCCCTACACGACGCTCTTCCGATCT GGTCATCCCTGGCCATAGAGC
*FANCF*_OT3_Adaptor_R	GTGACTGGAGTTCAGACGTGTGCTCTTCCGATCT

	CCTGGGTGACAGGGAGAAACC
*FANCF*_OT4_Adaptor_F	ACACTCTTTCCCTACACGACGCTCTTCCGATCT ATGGCAGCTATGGCCTCATGAAATG
*FANCF*_OT4_Adaptor_R	GTGACTGGAGTTCAGACGTGTGCTCTTCCGATCT AAGCCCATTGTTGAGATCTACTGCTTA
*FANCF*_OT5_Adaptor_F	ACACTCTTTCCCTACACGACGCTCTTCCGATCT CCCTTTGTTCTGATTTGGGTATTCTATG
*FANCF*_OT5_Adaptor_R	GTGACTGGAGTTCAGACGTGTGCTCTTCCGATCT TTAAACATGATTGACTCCCACCATCG
*FANCF*_OT6_Adaptor_F	ACACTCTTTCCCTACACGACGCTCTTCCGATCT TGCAATATGTGTCTTTCTGTGCTTAGC
*FANCF*_OT6_Adaptor_R	GTGACTGGAGTTCAGACGTGTGCTCTTCCGATCT TACATGCTGTGATTGATCTACTGTCTC
*FANCF*_OT7_Adaptor_F	ACACTCTTTCCCTACACGACGCTCTTCCGATCT GAGAAGTGGTGATAGAGGTCAACC
*FANCF*_OT7_Adaptor_R	GTGACTGGAGTTCAGACGTGTGCTCTTCCGATCT AGATAACCAAGGAGGTGTACAGATAAC
*FANCF*_OT8_Adaptor_F	ACACTCTTTCCCTACACGACGCTCTTCCGATCT CAGAAACTGGCCCACATCTACCG
*FANCF*_OT8_Adaptor_R	GTGACTGGAGTTCAGACGTGTGCTCTTCCGATCT GTGAGTCTTGTACTGAAATTCGCACTG
*FANCF*_OT9_Adaptor_F	ACACTCTTTCCCTACACGACGCTCTTCCGATCT TGAGCCCATTTCTACCAGATCACATG
*FANCF*_OT9_Adaptor_R	GTGACTGGAGTTCAGACGTGTGCTCTTCCGATCT AGGCTGAGTTGCCCAGGTAGC
*FANCF*_OT10_Adaptor_F	ACACTCTTTCCCTACACGACGCTCTTCCGATCT TCACTGCTTCAATCACCGGAGAAG
*FANCF*_OT10_Adaptor_R	GTGACTGGAGTTCAGACGTGTGCTCTTCCGATCT GGTGAAGCAAATACAATCAGTCCCTG
*FANCF*_OT11_Adaptor_F	ACACTCTTTCCCTACACGACGCTCTTCCGATCT GTTGACCTGTACCATGACTACCAC
*FANCF*_OT11_Adaptor_R	GTGACTGGAGTTCAGACGTGTGCTCTTCCGATCT GGCGACTGTCACCCAAGCCC
*FANCF*_OT12_Adaptor_F	ACACTCTTTCCCTACACGACGCTCTTCCGATCT ACACCTTGTGCTGAAGATGGATGC
*FANCF*_OT12_Adaptor_R	GTGACTGGAGTTCAGACGTGTGCTCTTCCGATCT TGCTTCCCATTGTTCTTGCTGTACG
*FANCF*_OT13_Adaptor_F	ACACTCTTTCCCTACACGACGCTCTTCCGATCT CTGTAGTGCCCTGTTGGCCTTTG

*FANCF*_OT13_Adaptor_R	GTGACTGGAGTTCAGACGTGTGCTCTTCCGATCT CGAAGAGGCAGACATTTATGAGTGG
*FANCF*_OT14_Adaptor_F	ACACTCTTTCCCTACACGACGCTCTTCCGATCT TGGCAGTTTGGGAGGCTGGAC
*FANCF*_OT14_Adaptor_R	GTGACTGGAGTTCAGACGTGTGCTCTTCCGATCT GCTTTACAACAAGACACTCTGCCAAG
*FANCF*_OT15_Adaptor_F	ACACTCTTTCCCTACACGACGCTCTTCCGATCT GAAGGCGTTGCTCTAGAGGACTAAGG
*FANCF*_OT15_Adaptor_R	GTGACTGGAGTTCAGACGTGTGCTCTTCCGATCT CCATATTCAAGAGACATGAGCCTCC
*FANCF*_OT16_Adaptor_F	ACACTCTTTCCCTACACGACGCTCTTCCGATCT TCCACCTACTTCGAGTACCTGATG
*FANCF*_OT16_Adaptor_R	GTGACTGGAGTTCAGACGTGTGCTCTTCCGATCT GTCTTTCTCCAAATGCCTCCCTTG
*HBB*_OT1_Adaptor_F	ACACTCTTTCCCTACACGACGCTCTTCCGATCT TGATACCAACCTGCCCAGGGC
*HBB*_OT1_Adaptor_R	GTGACTGGAGTTCAGACGTGTGCTCTTCCGATCT GCAGGGAGGACAGGACCAGC
*HBB*_OT2_Adaptor_F	ACACTCTTTCCCTACACGACGCTCTTCCGATCT TCTTGGACCAGAGGGAGGCAG
*HBB*_OT2_Adaptor_R	GTGACTGGAGTTCAGACGTGTGCTCTTCCGATCT GCTTGAGGTTCTGTAGGATGTCG
*HBB*_OT3_Adaptor_F	ACACTCTTTCCCTACACGACGCTCTTCCGATCT CGATGTGGCAGACGCATGGAC
*HBB*_OT3_Adaptor_R	GTGACTGGAGTTCAGACGTGTGCTCTTCCGATCT TTAGCCAGGATGGTCTCGATCTC
*HBB*_OT4_Adaptor_F	ACACTCTTTCCCTACACGACGCTCTTCCGATCT TATCTCCTCTGGGACCTGCCATG
*HBB*_OT4_Adaptor_R	GTGACTGGAGTTCAGACGTGTGCTCTTCCGATCT CTGTTCAGCCGTACCATGAGATGT
*HBB*_OT5_Adaptor_F	ACACTCTTTCCCTACACGACGCTCTTCCGATCT CAGGTTGCCCCTAGCTCCACC
*HBB*_OT5_Adaptor_R	GTGACTGGAGTTCAGACGTGTGCTCTTCCGATCT GAGCAAAACCCCAGTTCTACCAAAAG
*HBB*_OT6_Adaptor_F	ACACTCTTTCCCTACACGACGCTCTTCCGATCT ATGCTGGGTAACAAACAACTACAGAAC
*HBB*_OT6_Adaptor_R	GTGACTGGAGTTCAGACGTGTGCTCTTCCGATCT TGCTCTGGTGCCTCTGCCATC
*HBB*_OT7_Adaptor_F	ACACTCTTTCCCTACACGACGCTCTTCCGATCT

	GTCTGTCCCTGCCTCAGCTG
*HBB*_OT7_Adaptor_R	GTGACTGGAGTTCAGACGTGTGCTCTTCCGATCT CCTACAGGGGTCAGGGCTTG
*HBB*_OT8_Adaptor_F	ACACTCTTTCCCTACACGACGCTCTTCCGATCT CACCCACCACCAGAGACTCAG
*HBB*_OT8_Adaptor_R	GTGACTGGAGTTCAGACGTGTGCTCTTCCGATCT TGATCAGGTGGGTTTGAGGAGTC
*HBB*_OT9_Adaptor_F	ACACTCTTTCCCTACACGACGCTCTTCCGATCT ATCAAGCCGCTGCACTCCAGC
*HBB*_OT9_Adaptor_R	GTGACTGGAGTTCAGACGTGTGCTCTTCCGATCT AGTCAAGGGAGCACTCCTCATAATG
*HBB*_OT10_Adaptor_F	ACACTCTTTCCCTACACGACGCTCTTCCGATCT AATTGTGCTCCAAGGAACCCCTG
*HBB*_OT10_Adaptor_R	GTGACTGGAGTTCAGACGTGTGCTCTTCCGATCT GAGCATGTCCTATGTGCCAGGC
*HBB*_OT11_Adaptor_F	ACACTCTTTCCCTACACGACGCTCTTCCGATCT GTCCCTGCAGCGTCTGAGACC
*HBB*_OT11_Adaptor_R	GTGACTGGAGTTCAGACGTGTGCTCTTCCGATCT CTCCTTTGCTCTCTCTGCCTGG
*HBB*_OT12_Adaptor_F	ACACTCTTTCCCTACACGACGCTCTTCCGATCT CAGGCATGAGCCACTGCGCC
*HBB*_OT12_Adaptor_R	GTGACTGGAGTTCAGACGTGTGCTCTTCCGATCT GATGTTTGTAAAACAATTAGAGAGTCCTGTG
*HBB*_OT13_Adaptor_F	ACACTCTTTCCCTACACGACGCTCTTCCGATCT CTATGTAACTCCCACTCCCCAGG
*HBB*_OT13_Adaptor_R	GTGACTGGAGTTCAGACGTGTGCTCTTCCGATCT CCCTCGTAGTCTTCCAGGTCCC
*HBB*_OT14_Adaptor_F	ACACTCTTTCCCTACACGACGCTCTTCCGATCT CAAGGCGAGGACAGAATGGATGC
*HBB*_OT14_Adaptor_R	GTGACTGGAGTTCAGACGTGTGCTCTTCCGATCT CAGCTTAATGGCTGGAGAAGAAGAC
*HBB*_OT15_Adaptor_F	ACACTCTTTCCCTACACGACGCTCTTCCGATCT GGTTGCTAACTAGGGGCCATCC
*HBB*_OT15_Adaptor_R	GTGACTGGAGTTCAGACGTGTGCTCTTCCGATCT GACAACCCCATTTTAAGCTCTGGTG
*HBB*_OT16_Adaptor_F	ACACTCTTTCCCTACACGACGCTCTTCCGATCT CACCAGCCTCATTTCCAGCCAG
*HBB*_OT16_Adaptor_R	GTGACTGGAGTTCAGACGTGTGCTCTTCCGATCT GGATACAGTGATGAATTTGGCTCTG

Table S3. Sequence information of *in-silico* base predicted off-target sites for each target sites measured in this study. Target and off-target sequences and position on the chromosome are indicated, respectively. Mismatch sequences are shown in red. PAM sequences (NGG) in the target DNA are shown in orange.

NRAS

*NRAS*_ On-target	GGTAAGGGGGCAGGGAGGGA**GGG**	Chr1	114713992
*NRAS*_OT1	GGGAAGGGGGCAGGGAGGGA**GGG**	Chr22	48443553
*NRAS*_OT2	GGGAAGGGGGCAGGGAGGGA**GGG**	Chr2	220446617
*NRAS*_OT3	GGGAAGGGGGCAAGGAGGGA**AGG**	Chr8	140165600
*NRAS*_OT4	GGTAAGGGAGAAGGGAGGGA**AGG**	Chr5	79154620
*NRAS*_OT5	GGTAAGGGGGCAGAGAGAGA**AGG**	Chr1	36327044
*NRAS*_OT6	GGGAAGGGTGCAGGGAGGGA**GGG**	Chr2	29024457
*NRAS*_OT7	GGgAAGGaGGCAGGGAGGGAAGG	Chr8	36356746
*NRAS*_OT8	GGgAAGGGGGCAGGGAGGGgCGG	Chr15	90867066
*NRAS*_OT9	GGcAgGGGGGCAGGGAGGGATGG	Chr15	70449060
*NRAS*_OT10	aGaAAGGGGGCAGGGAGGGACGG	Chr1	10828266
*NRAS*_OT11	GGaAAGGGGGCAGtGAGGGAAGG	Ch22	36782545
*NRAS*_OT12	GGTtgGGGGGCAGGGAGGGAAGG	Chr7	105197089
*NRAS*_OT13	GGaAAGGGGGCAGGGAaGGAAGG	Chr12	4135155
*NRAS*_OT14	aGTAAGGGGGgAGGGAGGGAGGG	Chr12	

		102632319
*NRAS*_OT15	GGTgAGGGGGaAGGGAGGGAGGG	Chr19<br>2504989
*NRAS*_OT16	GGgAAGGaGGCAGGGAGGGAAGG	Chr4<br>11367071

C-Myc

*c-Myc*_ On-target	AGGCAGAGGGAGCGAGCGGGCGG	Chr8<br>127736255
*c-Myc*_OT1	AGGCAGAGGGAGCcAGaGGGAGG	Chr2<br>236847262
*c-Myc*_OT2	AGGCAGgGGGAGCGAaCGGGCGG	Chr17<br>7214441
*c-Myc*_OT3	AGGCAGAGGGAGCcAGaGGGAGG	Chr16<br>9415767
*c-Myc*_OT4	AGGCgGAGGGAGCGAGCGGcGGG	Chr10<br>95560813
*c-Myc*_OT5	AGGCAGAGGGAGgGAGCGcGGGG	Chr3<br>61251329
*c-Myc*_OT6	AGGCAGAGGGAGtGtGaGGGAGG	Chr8<br>20816244
*c-Myc*_OT7	AGGggGAGGaAGCGAGCGGGAGG	Chr2<br>48314633
*c-Myc*_OT8	gGGCgGAGGGAGCGAGgGGGAGG	Chr12<br>116533842
*c-Myc*_OT9	AaGgAGAGGGAGCGAGCaGGGGG	Chr16<br>6478722
*c-Myc*_OT10	gGGgAGAGGGAGCGgGCGGGCGG	Chr16<br>12995428
*c-Myc*_OT11	AGaaAGAGGGAGCGgGCGGGTGG	Chr5<br>57634154

*c-Myc*_OT12	AaGCcGAGGGAGCGtGCGGGCGG	Chr1<br>202348677
*c-Myc*_OT13	gGGaAGAGGGAGgGAGCGGGCGG	Chr2<br>39665877
*c-Myc*_OT14	AGGCAGAGGGAGCGgGCacGGGG	Chr21<br>42514269
*c-Myc*_OT15	AGGgAGgGGGAGaGAGCGGGAGG	Chr17<br>45579997
*c-Myc*_OT16	AGGgAGAGGGAGgGAGaGGGAGG	Chr19<br>4702844

HEK3-4

*HEK3-4*_ On-target	GGATTGACCCAGGCCAGGGCTGG	Chr9<br>107422311
*HEK3-4*_OT1	GGAggGACCCAGGCCtGGGCAGG	Chr15<br>20877563
*HEK3-4*_OT2	GcATTGACaCAGGCCAGGGaGGG	Chr15<br>65373203
H*HEK3-4*_OT3	GGATTGgCCCAGGggAGGGCAGG	Chr15<br>75661078
*HEK3-4*_OT4	GGcTTGtCCCAGGCCAGaGCTGG	Chr20<br>33287626
*HEK3-4*_OT5	aGgTTGgCCCAGGCCAGGGCGGG	Chr1<br>24077815
*HEK3-4*_OT6	aGATgGACaCAGGCCAGGGCAGG	Chr1<br>29650705
*HEK3-4*_OT7	GGtgTGgCCCAGGCCAGGGCTGG	Chr1<br>38075444
*HEK3-4*_OT8	GGAagGtCCCAGGCCAGGGCAGG	Chr1<br>227942100
*HEK3-4*_OT9	GGAaTGACCCAGcCCAGGGtGGG	Chr1

		232780686
*HEK3-4*_OT10	aGAcTGcCCCAGGCCAGGGCCGG	Chr16<br>2702490
*HEK3-4*_OT11	GGAggGACCCAGGCCtGGGCAGG	Chr15<br>21451256
*HEK3-4*_OT12	GGAggGACCCAGGCCtGGGCAGG	Chr15<br>21888537
*HEK3-4*_OT13	GGAaTGAagCAGGCCAGGGCAGG	Chr20<br>4071839
*HEK3-4*_OT14	GGAgTGtCCCAGGCCAGaGCAGG	Chr1<br>1085776
*HEK3-4*_OT15	GGATTGtCCaAGGCtAGGGCAGG	Chr16<br>49847555
*HEK3-4*_OT16	GGtgTGACCCAGGCCAGGcCAGG	Chr19<br>31330491

FANCF

*FANCF*_ On-target	GGAATCCCTTCTGCAGCACCTGG	Chr11<br>22625785
*FANCF*_OT1	GGAATatCTTCTGCAGCcCCAGG	Chr2<br>54626173
*FANCF*_OT2	GGAATCaCTTtTaCAGCACCAGG	Chr16<br>85902632
*FANCF*_OT3	ctcATCCCTTCTGCAGCcCCAGG	Chr10<br>13394771
*FANCF*_OT4	aGtATCCaTTCTGCAGCACaTGG	ChrX<br>111476496
*FANCF*_OT5	aaAATCCaTTCTGCAGCACaAGG	Chr12<br>121725196
*FANCF*_OT6	aGggTCCCTTCTGCAGCcCCTGG	Chr12<br>115029996

*FANCF*_OT7	aGAATCtCTTCTcCAaCACCTGG	Chr4<br>140590966
*FANCF*_OT8	GGAAcCCCcTCTGCAGCttCTGG	Chr17<br>4077075
*FANCF*_OT9	GGAATtCCTTCTGCAGCtatTGG	Chr10<br>128974393
*FANCF*_OT10	GGAggCCCcTCTGCAGCACtCTG	Chr17<br>73973234
*FANCF*_OT11	GGAgTCCCccCTGCAGCACtCTG	Chr20<br>62128029
*FANCF*_OT12	GGAgTCaCTTCTGCAGacaCCGG	Chr7<br>158655424
*FANCF*_OT13	ccAtcCCCTaCTGCAGCACCTGG	Chr20<br>821996
*FANCF*_OT14	ccccTCCCTcCTGCAGCACCGGG	Chr22<br>45476093
*FANCF*_OT15	GGAATCCtTTaTGCAGCACaCAG	Chr11<br>45280287
*FANCF*_OT16	acAcTCCCTTCTGCAGCACCATG	Chr12<br>2610722

HBB

*HBB*_ On-target	CATGGTGCACCTGACTCCTGAGG	Chr11<br>5248236
*HBB*_OT1	CATGGTGCAtCTGACTCCTGAGG	Chr11<br>5234412
*HBB*_OT2	CtTGaTtCACCTGACTCCTGCGG	Chr10<br>73801329
*HBB*_OT3	gATaGTGCACaTGACTCCTGCGG	Chr20<br>61770556

*HBB*_OT4	CATGGTaCAgCTGgCTCCTGCGG	Chr16<br>8625664
*HBB*_OT5	CcgGcTGCAaCTGACTCCTGGGG	Chr19<br>38049597
*HBB*_OT6	CcTGGctCAgCTGACTCCTGGGG	Chr4<br>184002509
*HBB*_OT7	tATGGctCcCCTGACTCCTGCGG	Chr1<br>16705096
*HBB*_OT8	CAgGcTGtgCCTGACTCCTGGGG	Chr5<br>137614148
*HBB*_OT9	CAccGTGCAtCTGACTCCTaGGG	Chr9<br>131095711
*HBB*_OT10	CATGcTGCcaCTGACTCCaGGGG	Chr11<br>44691624
*HBB*_OT11	CAgccTGCACCTcACTCCTGCGG	Chr18<br>79524587
*HBB*_OT12	aAgGaTGCACCTGACcCCTGGGG	Chr5<br>80237160
*HBB*_OT13	CAcGGaGgACCTGACaCCTGGGG	Chr7<br>99468514
*HBB*_OT14	CAgcGTGCgCaTGACTCCTGGGG	Chr2<br>4022773
*HBB*_OT15	CAaGGaGCAaCTaACTCCTGGGG	Chr17<br>8091685
*HBB*_OT16	CcTGGTtCAtCTGACTCCaGAGG	Chr1<br>11453146

Table S4. Sequence information of sgRNA used for in-vitro cleavage assay.

Target gene	CRISPR-Cas9 (FnCas9) target sequence (5′ to 3′)	sgRNA sequence for FnCas9 (5′ to 3′)
c-Myc	AGGCAGAGGGAGCGAGCGGGCGG	AGGCAGAGGGAGCGAGCGGGGUUUCAGUU GCUGAAAAUGCUCUGUAAUCAUUUAAAAGU AUUUUGAACGGACCUCUGUUUGACACGUCU GAAUAACUAAAAA

AAVS1	TCTAACCCCCACCTCCTGTTAGG	UCUAACCCCCACCUCCUGUUGUUUCAGUUGCUGAAAAUGCUCUGUAAUCAUUUAAAAGUAUUUUGAACGGACCUCUGUUUGACACGUCUGAAUAACUAAAAA
EMX1	TGGTTGCCCACCCTAGTCATTGG	UGGUUGCCCACCCUAGUCAUGUUUCAGUUGCUGAAAAUGCUCUGUAAUCAUUUAAAAGUAUUUGAACGGACCUCUGUUUGACACGUCUGAAUAACUAAAAA
HEK3	GGCCCAGACTGAGCACGTGATGG	GGCCCAGACUGAGCACGUGAGUUUCAGUUGCUGAAAAUGCUCUGUAAUCAUUUAAAAGUAUUUGAACGGACCUCUGUUUGACACGUCUGAAUAACUAAAAA
NRAS	GGTAAGGGGGCAGGGAGGGAGGG	GGUAAGGGGGCAGGGAGGGAGUUUCAGUUGCUGAAAAUGCUCUGUAAUCAUUUAAAAGUAUUUGAACGGACCUCUGUUUGACACGUCUGAAUAACUAAAAA

Target gene	CRISPR-Cas9 (SpCas9) target sequence (5′-3′)	sgRNA sequence for SpCas9 (5′-3′)
c-Myc	AGGCAGAGGGAGCGAGCGGGCGG	AGGCAGAGGGAGCGAGCGGGGUUUUAGAGCUAGAAAUAGCAAGUUAAAAUAAGGCUAGUCCGUUAUCAACUUGAAAAAGUGGCACCGAGUCGGUGCUUUUUU
AAVS1	TCTAACCCCCACCTCCTGTTAGG	UCUAACCCCCACCUCCUGUUGUUUUAGAGCUAGAAAUAGCAAGUUAAAAUAAGGCUAGUCCGUUAUCAACUUGAAAAAGUGGCACCGAGUCGGUGCUUUUUU
EMX1	TGGTTGCCCACCCTAGTCATTGG	UGGUUGCCCACCCUAGUCAUGUUUUAGAGCUAGAAAUAGCAAGUUAAAAUAAGGCUAGUCCGUUAUCAACUUGAAAAAGUGGCACCGAGUCGGUGCUUUUUU
HEK3	GGCCCAGACTGAGCACGTGATGG	GGCCCAGACUGAGCACGUGAGUUUUAGAGCUAGAAAUAGCAAGUUAAAAUAAGGCUAGUCCGUUAUCAACUUGAAAAAGUGGCACCGAGUCGGUGCUUUUUU
NRAS	GGTAAGGGGGCAGGGAGGGAGGG	GGUAAGGGGGCAGGGAGGGAGUUUUAGAGCUAGAAAUAGCAAGUUAAAAUAAGGCUAGUCCGUUAUCAACUUGAAAAAGUGGCACCGAGUCGGUGCUUUUUU

†PAM sequences (NGG) in the target DNA are shown in orange

Supplementary Figures

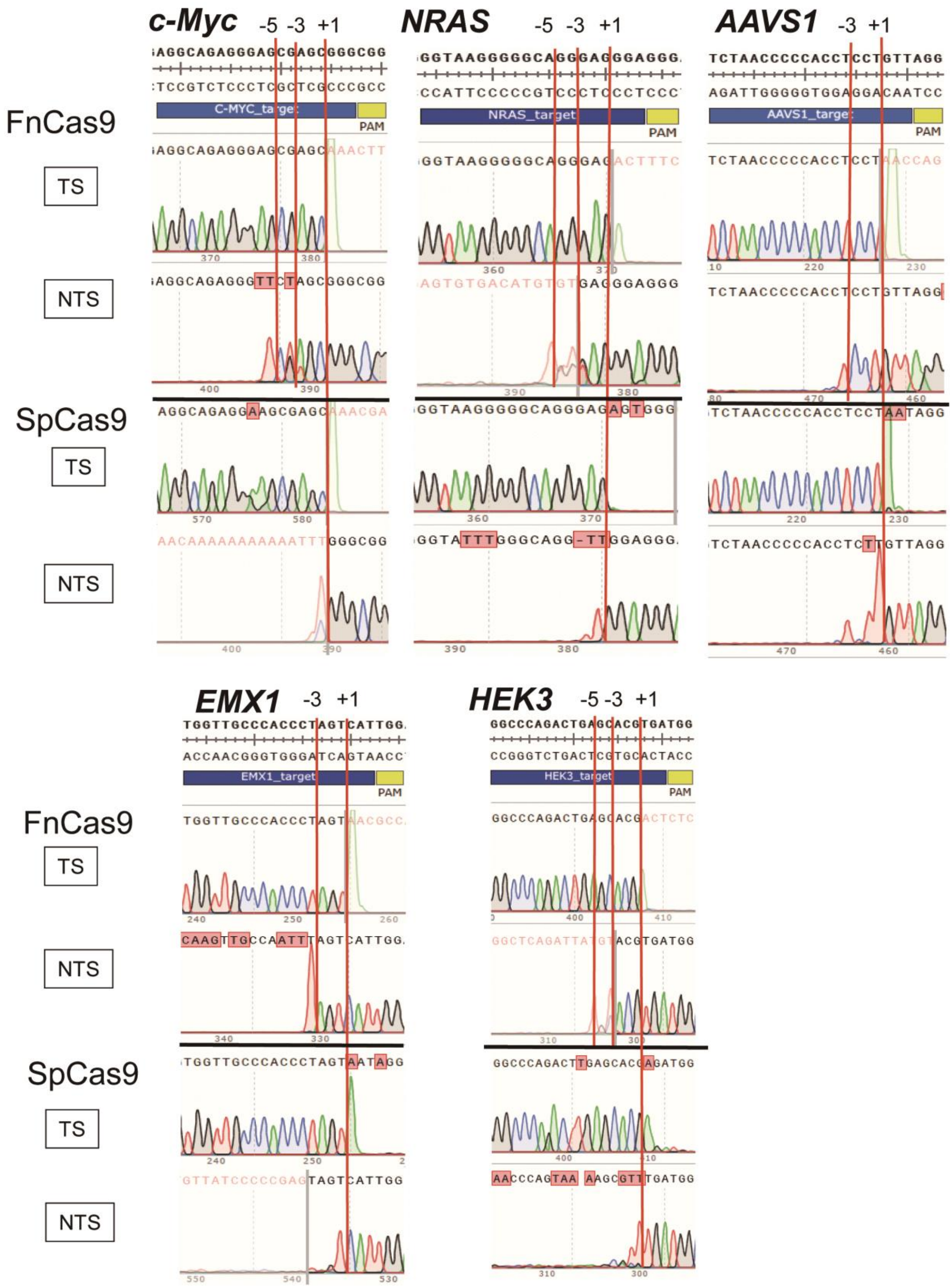

Figure S1. Analysis of the cleavage point of FnCas9 and SpCas9. Plasmid containing the target nucleotide sequence (*c-Myc, AAVS1, EMX1, HEK3, NRAS*) was digested with FnCas9 and SpCas9, respectively, and then analyzed for

comparison using run-off Sanger sequencing (top: double-stranded DNA cleavage point by FnCas9, bottom: double-stranded DNA cleavage point by SpCas9). In each nucleotide sequence (*c-Myc, AAVS1, EMX1, HEK3, NRAS*), PAM(NGG) is indicated in yellow and protospacer is indicated in blue. The positions cut by FnCas9 and SpCas9 in the non-target strand (NTS) and target strands (TS) are indicated by red lines, respectively.

a

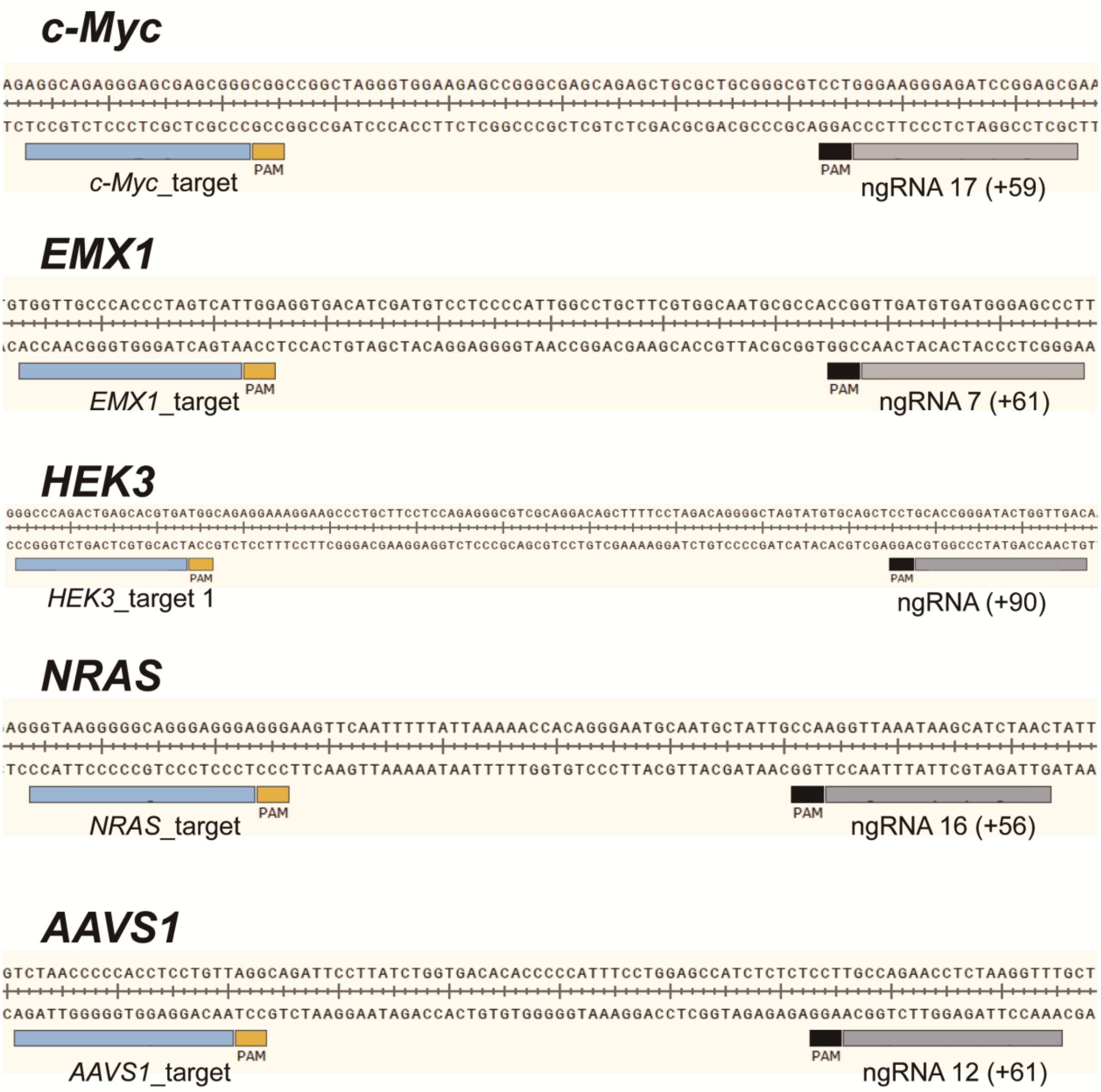
c-Myc
c-Myc_target
PAM
PAM
ngRNA 17 (+59)
EMX1
EMX1_target
PAM
PAM
ngRNA 7 (+61)
HEK3
HEK3_target 1
PAM
PAM
ngRNA (+90)
NRAS
NRAS_target
PAM
PAM
ngRNA 16 (+56)
AAVS1
AAVS1_target
PAM
PAM
ngRNA 12 (+61)

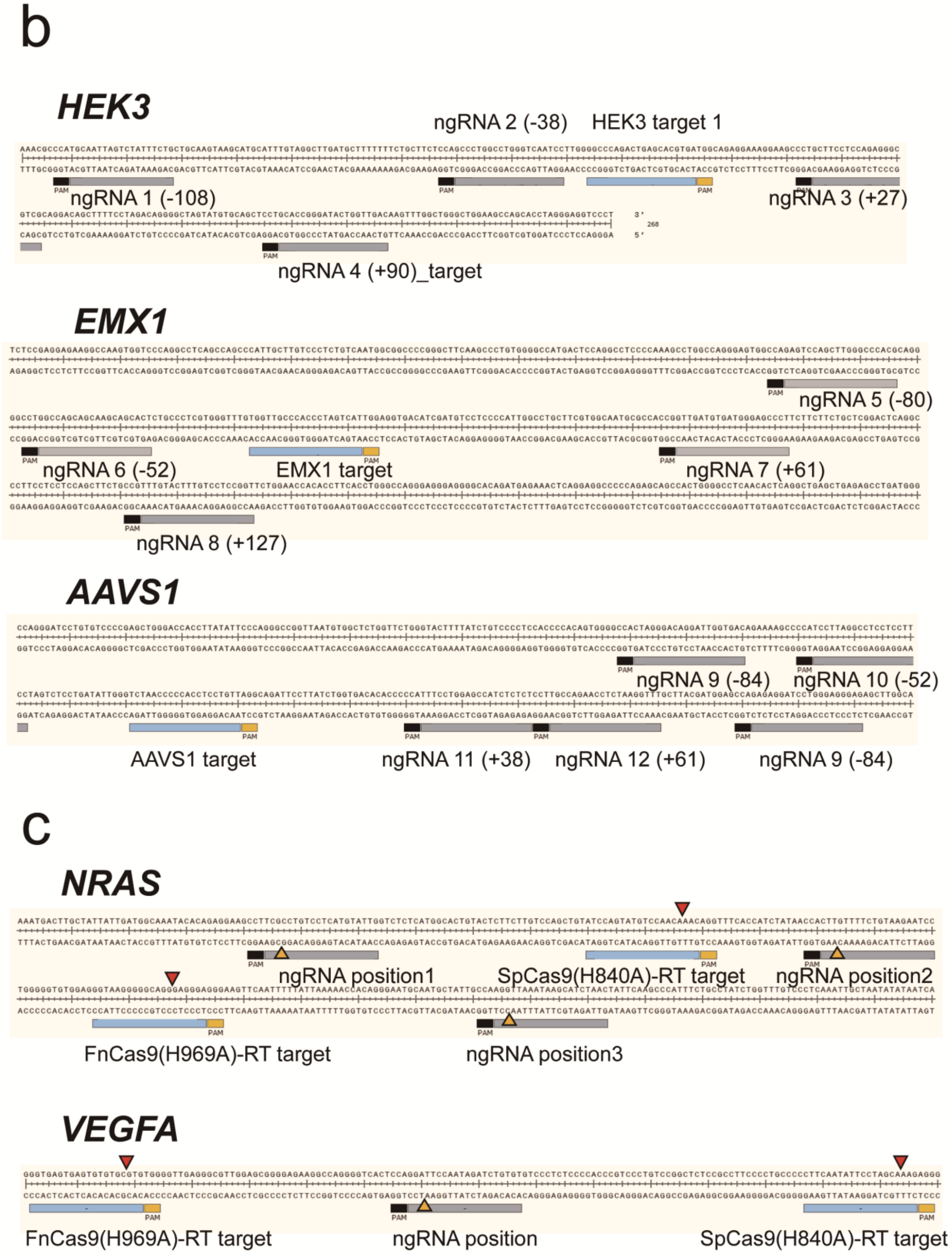
b
HEK3
ngRNA 2 (-38)
HEK3 target 1
ngRNA 1 (-108)
ngRNA 3 (+27)
ngRNA 4 (+90)_target
EMX1
ngRNA 5 (-80)
ngRNA 6 (-52)
EMX1 target
ngRNA 7 (+61)
ngRNA 8 (+127)
AAVS1
ngRNA 9 (-84)
ngRNA 10 (-52)
AAVS1 target
ngRNA 11 (+38)
ngRNA 12 (+61)
ngRNA 9 (-84)
c
NRAS
ngRNA position1
SpCas9(H840A)-RT target
ngRNA position2
FnCas9(H969A)-RT target
ngRNA position3
VEGFA
FnCas9(H969A)-RT target
ngRNA position
SpCas9(H840A)-RT target

d

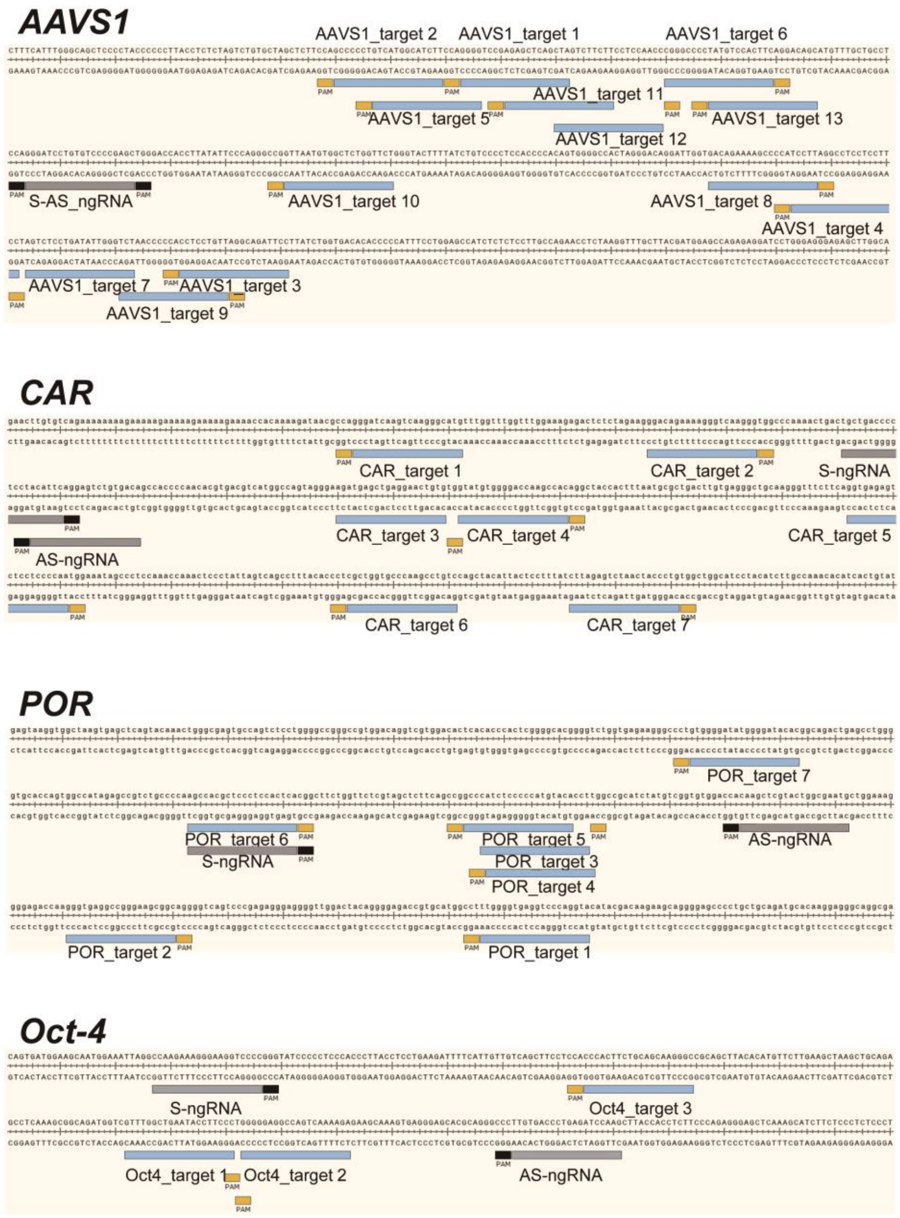
AAVS1
AAVS1_target 2
AAVS1_target 1
AAVS1_target 6
AAVS1_target 11
AAVS1_target 5
AAVS1_target 13
AAVS1_target 12
S-AS_ngRNA
AAVS1_target 10
AAVS1_target 8
AAVS1_target 4
AAVS1_target 7
AAVS1_target 3
AAVS1_target 9
PAM
CAR
CAR_target 1
CAR_target 2
S-ngRNA
CAR_target 3
CAR_target 4
CAR_target 5
AS-ngRNA
CAR_target 6
CAR_target 7
POR
POR_target 7
POR_target 6
POR_target 5
AS-ngRNA
S-ngRNA
POR_target 3
POR_target 4
POR_target 2
POR_target 1
Oct-4
S-ngRNA
Oct4_target 3
Oct4_target 1
Oct4_target 2
AS-ngRNA

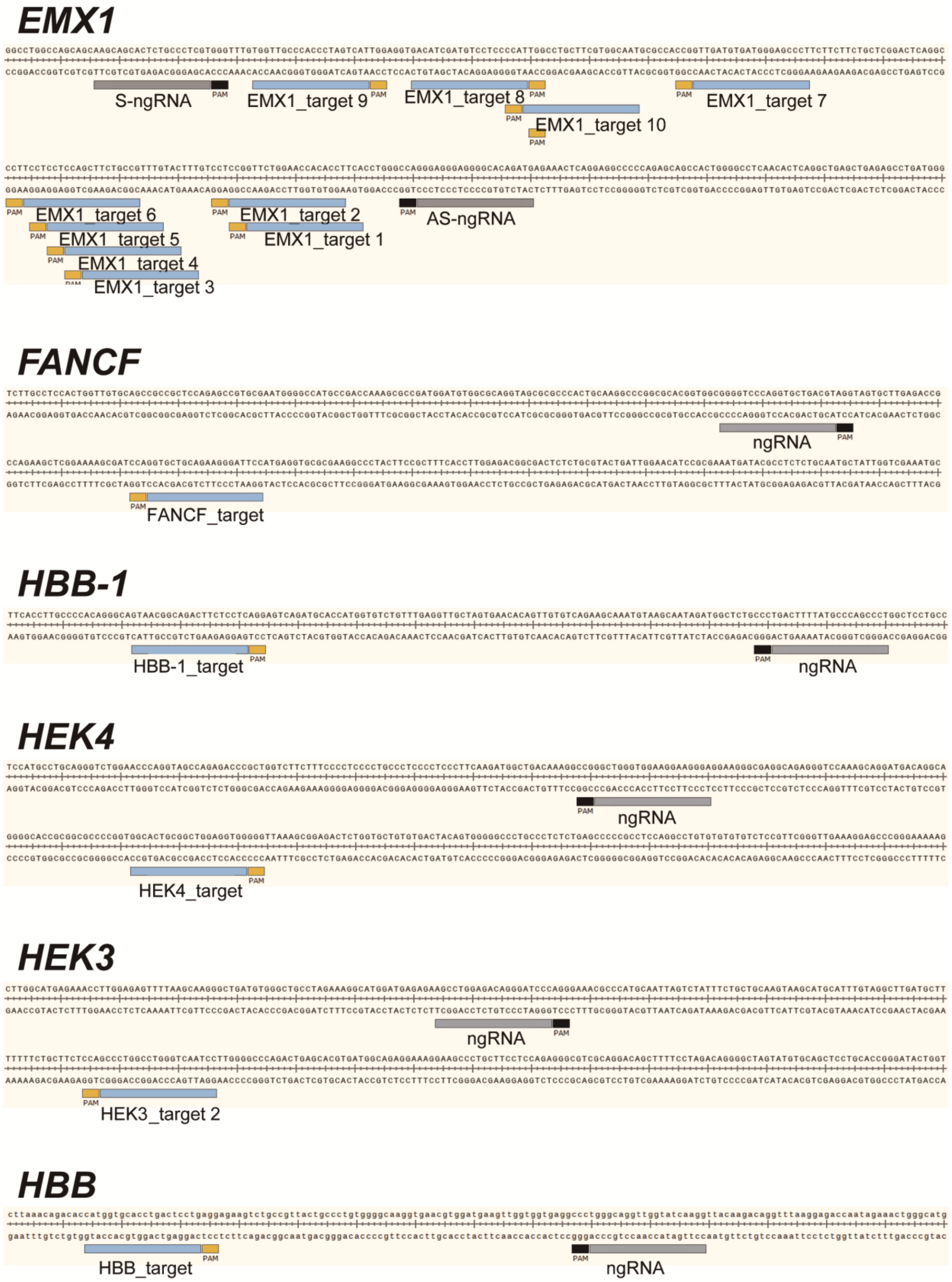

Figure S2. Shared target sequence and editing method by using SpCas9(H840A)-RT and FnCas9(H969A)-RT. a, Target sequences within a variety of commonly targeted genes (*c-Myc, EMX1, HEK3, NRAS, AAVS1*) which edited by

using SpCas9(H840A)-RT and FnCas9(H969A)-RT. **b,** FnCas9(H969A)-RT targeting target sequence and ngRNA target nucleotide sequence for prime editing by PE3 method. **c,** Targeted sequences for multiplexed prime editing by SpCas9(H840A)-RT and FnCas9(H969A)-RT and ngRNA target sequence for prime editing by PE3 method. The nick on the non-target strand side generated by FnCas9(H969A)-RT and SpCas9(H840A)-RT is indicated by red triangle, and the nick on target-strand side by ngRNA is indicated by yellow triangle, respectively. **d,** Target sequences for comparative analysis between SpCas9(H840A)-RT and FnCas9(H969A)-RT. The protospacer and PAM (NGG) sequences in the pegRNA target and ngRNA target are shown in light blue, yellow, gray and black, respectively. pegRNA: prime editing guideRNA, ngRNA: nicking guideRNA. The number in parentheses indicates the number of (bp) between the two nicks formed by the targeting of pegRNA and

ngRNA, respectively.

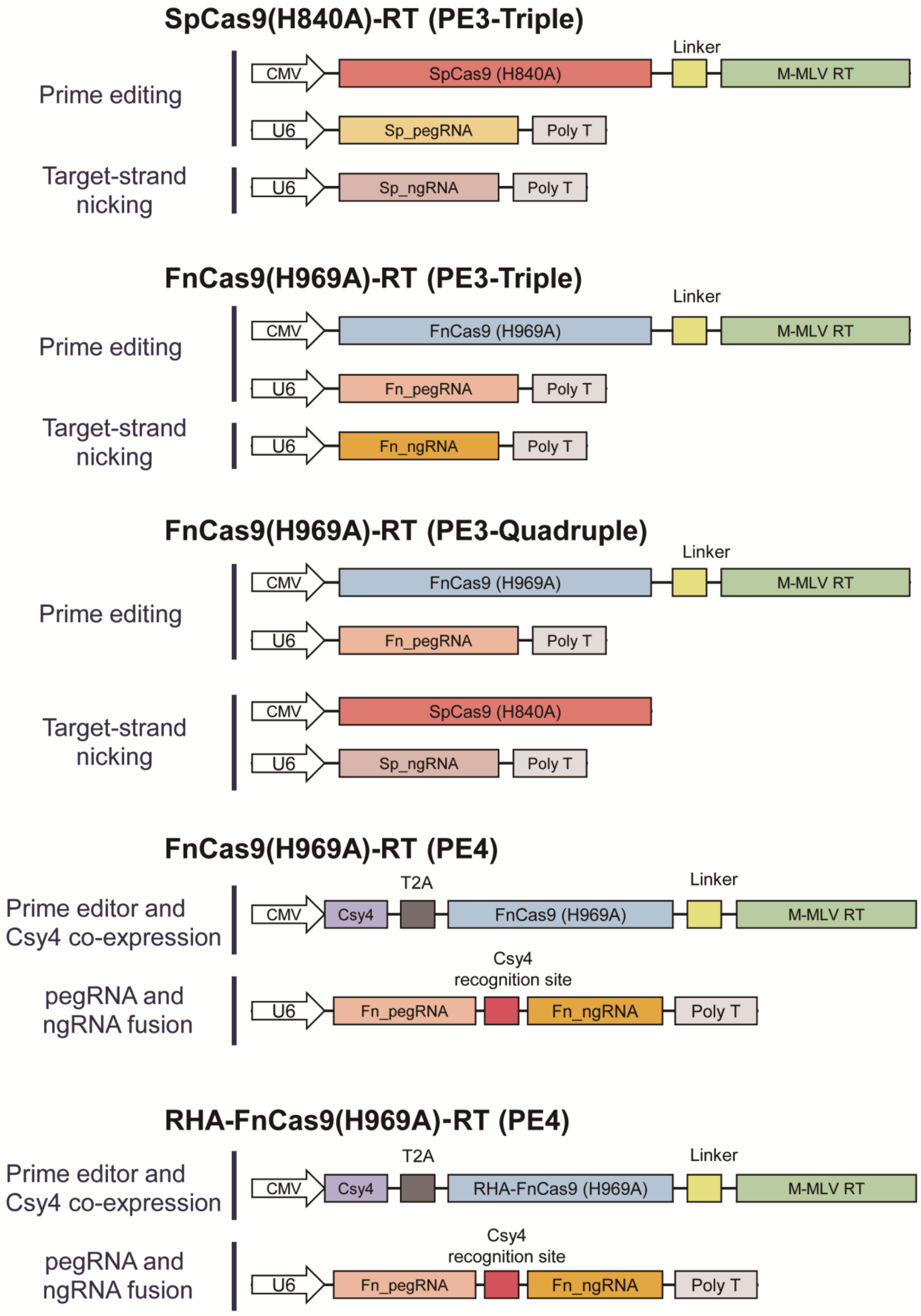

Figure S3. Various methods of prime editor delivery by using SpCas9(H840A)-RT or FnCas9(H969A)-RT expression plasmids. Prime editing strategy using SpCas9(H840A)-RT, FnCas9(H969A)-RT and ngRNAs. In the case of FnCas9(H969A)-RT, triple and quadruple versions of delivery are used for PE3 method. PE3_Triple: Both pegRNA(prime editing guideRNA) and ngRNA(nicking guideRNA) are used as FnCas9 modules. PE3_Quadruple: pegRNA is used as FnCas9 module, ngRNA is used as SpCas9 module, respectively. PE4: Csy4 and FnCas9(H969A)-RT genes are expressed through T2A, respectively, and pegRNA and ngRNA for FnCas9(H969A)-RT are continuously expressed based on the U6 promoter and cleaved by Csy4. Both pegRNA(prime editing guideRNA) and ngRNA(nicking guideRNA) are used as FnCas9 modules. Csy4: CRISPR RNA endonuclease, T2A: 2A self-cleaving peptide from thosea asigna virus. MMLV-RT: Reverse transcriptase domain from Moloney Murine Leukemia Virus, pegRNA: prime editing guideRNA, ngRNA: nicking guideRNA, SpCas9(H840A)-RT: Prime editor based on SpCas9(H840A) nickase, FnCas9(H969A)-RT: Prime editor based on FnCas9(H969A) nickase.

a

HEK3

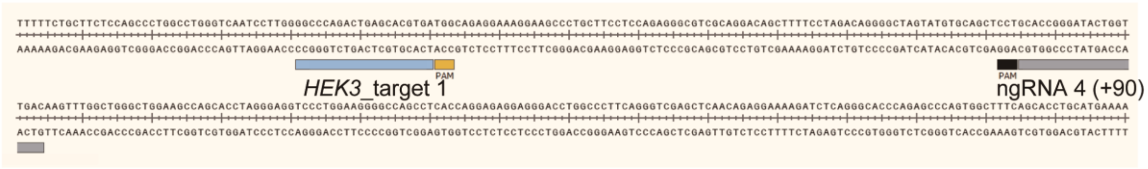

b

SpCas9(H840A)-RT, Sp_PE3 (Triple)

GGGGCCCAGACTGAGCACGTGATGGCAGAGGAAAGGAAGCCCT **Wild-type**

GGGGCCCAGACTGAGCACGCTTTGATGGCAGAGGAAAGGAAGCCCT **Precise 'CTT' insertion**

3bp

GGGGCCCAGACTGAGCACGCTTTGATGG--------------------------CAGAGGG **'CTT' insertion with indel**

3bp

GGGGCCCAGACTGAGCACGTGATGG-------------------------CAGAGGG **Indel**

FnCas9(H969A)-RT, Fn_PE3 (Triple)

GGGGCCCAGACTGAGCACGTGATGGCAGAGGAAAGGAAGCCCT **Wild-type**

GGGGCCCAGACTGAGCTTACGTGATGGCAGAGGAAAGGAAGCCCT **Precise 'TT' insertion**

6bp

GGGGC---CCAGAGGGC **Indel**

FnCas9(H969A)-RT, Fn_PE3 (Quadruple)

GGGGCCCAGACTGAGCACGTGATGGCAGAGGAAAGGAAGCCCT **Wild-type**

GGGGCCCAGACTGAGCTTACGTGATGGCAGAGGAAAGGAAGCCCT **Precise 'TT' insertion**

6bp

GGGGCCCAGACTGAGCTTACGTGATG ------------------TTTTTAGCTTC **'TT' insertion with indel**

6bp

GGGGCCCAGACTGAGC---------------------------CTTCCTCCAG **Indel**

c

c-Myc

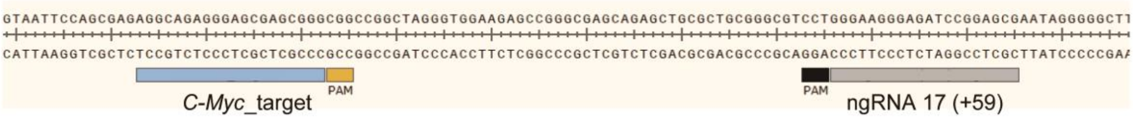

d

SpCas9(H840A)-RT, Sp_PE3 (Triple)

AGGCAGAGGGAGCGAGCGGGCGGCCGGCTAGGGTGG — **Wild-type**

AGGCAGAGGGAGCGAGCAAGGGCGGCCGGCTAGGGTGG — **Precise 'AA' insertion**

3bp

AGGCAGAGGGAGCGAGCAAGGGCGGCCGGCTAGGGTGGAAGAGCCGGGCGAGCAGAGCTGCGCTG

3bp — **'AA' insertion with indel**

AGGCAGAGGGA--------------------------------GGGCGAGCAGAGCTG — **Indel**

FnCas9(H969A)-RT, Fn_PE3 (Triple)

AGGCAGAGGGAGCGAGCGGGCGGCCGGCTAGGGTGG — **Wild-type**

AGGCAGAGGGAGCGAAAGCGGGCGGCCGGCTAGGGTGG — **Precise 'AA' insertion**

6bp

AGGCAGAGGGAGCGAAAGCGGGC------------------------TGCGAGCAGAGCTG — **'AA' insertion with indel**

6bp

AG--GCAGAG — **Indel**

FnCas9(H969A)-RT, Fn_PE3 (Quadruple)

AGGCAGAGGGAGCGAGCGGGCGGCCGGCTAGGGTGG — **Wild-type**

AGGCAGAGGGAGCGAAAGCGGGCGGCCGGCTAGGGTGG — **Precise 'AA' insertion**

6bp

AGGCAGAGGGAGCGAAAGCGGGCGGCC------GGTGGAAGAGCCGGG — **'AA' insertion with indel**

6bp

AGGCAGAGG--------------------------------------GAGCAGAGCTGCGC — **Indel**

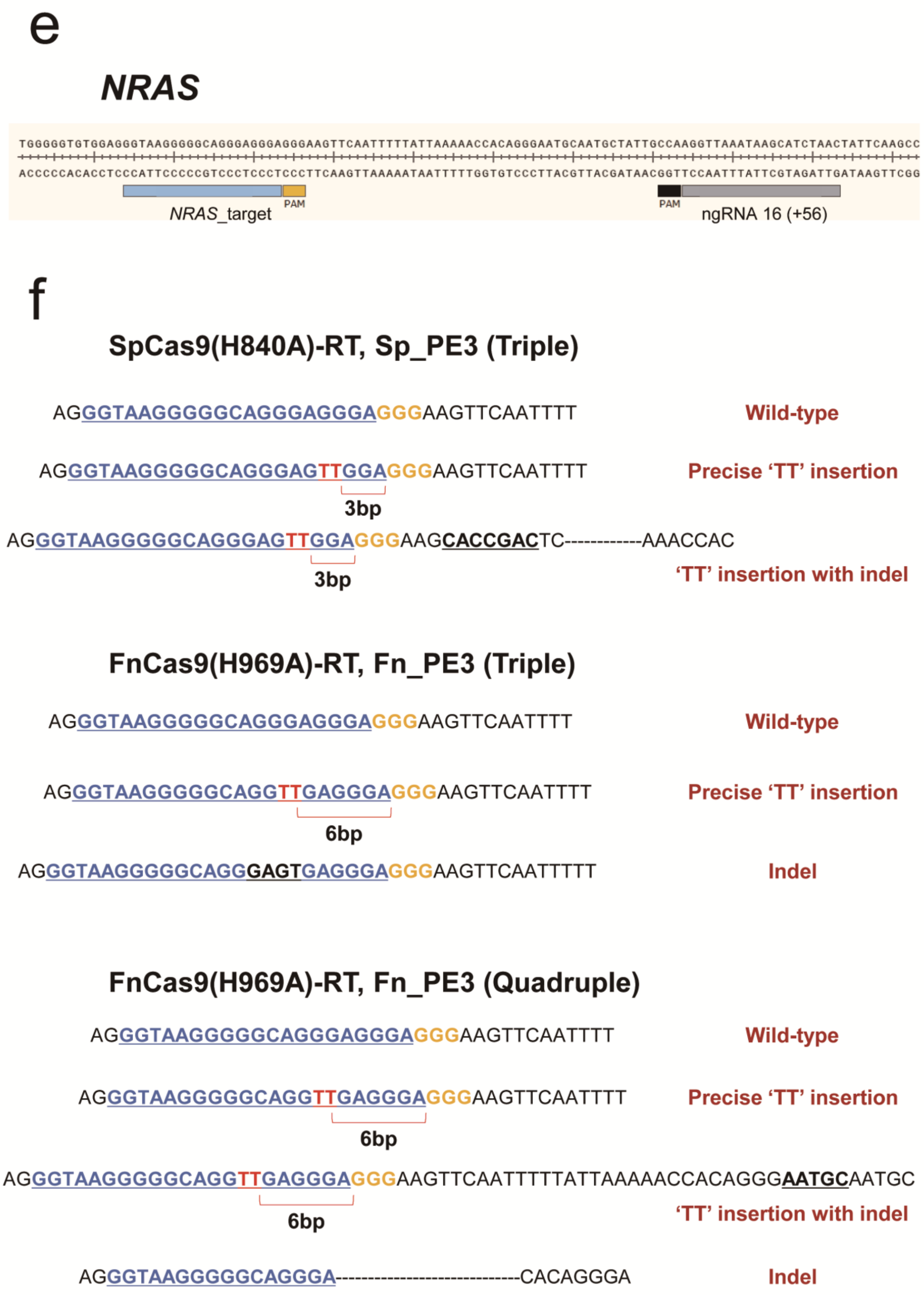

Figure S4. The target sequence and NGS analysis results commonly edited by SpCas9(H840A)-RT or FnCas9(H969A)-RT. a, c, e, Indication of pegRNA target

sequences and ngRNA sequences of SpCas9(H840A)-RT and FnCas9(H969A)-RT for *HEK3, c-Myc,* and *NRAS* sites. The protospacer and PAM (NGG) sequences in the pegRNA target and ngRNA target are shown in light blue, yellow, gray and black, respectively. **b, d, f,** NGS analysis results of target-specific base (TT, AA) insertion by SpCas9(H840A)-RT and FnCas9(H969A)-RT. The results of base insertion using SpCas9(H840A)-RT, FnCas9(H969A)-RT, pegRNA, and ngRNA in the triple or quadruple delivery manner are shown **(Additional file: Figure S3)**. Protospacers are shown in blue, PAM(NGG) sequences are shown in yellow, and inserted sequence TT or AA are shown in red, respectively. Dashed line indicates a deletion of bases.

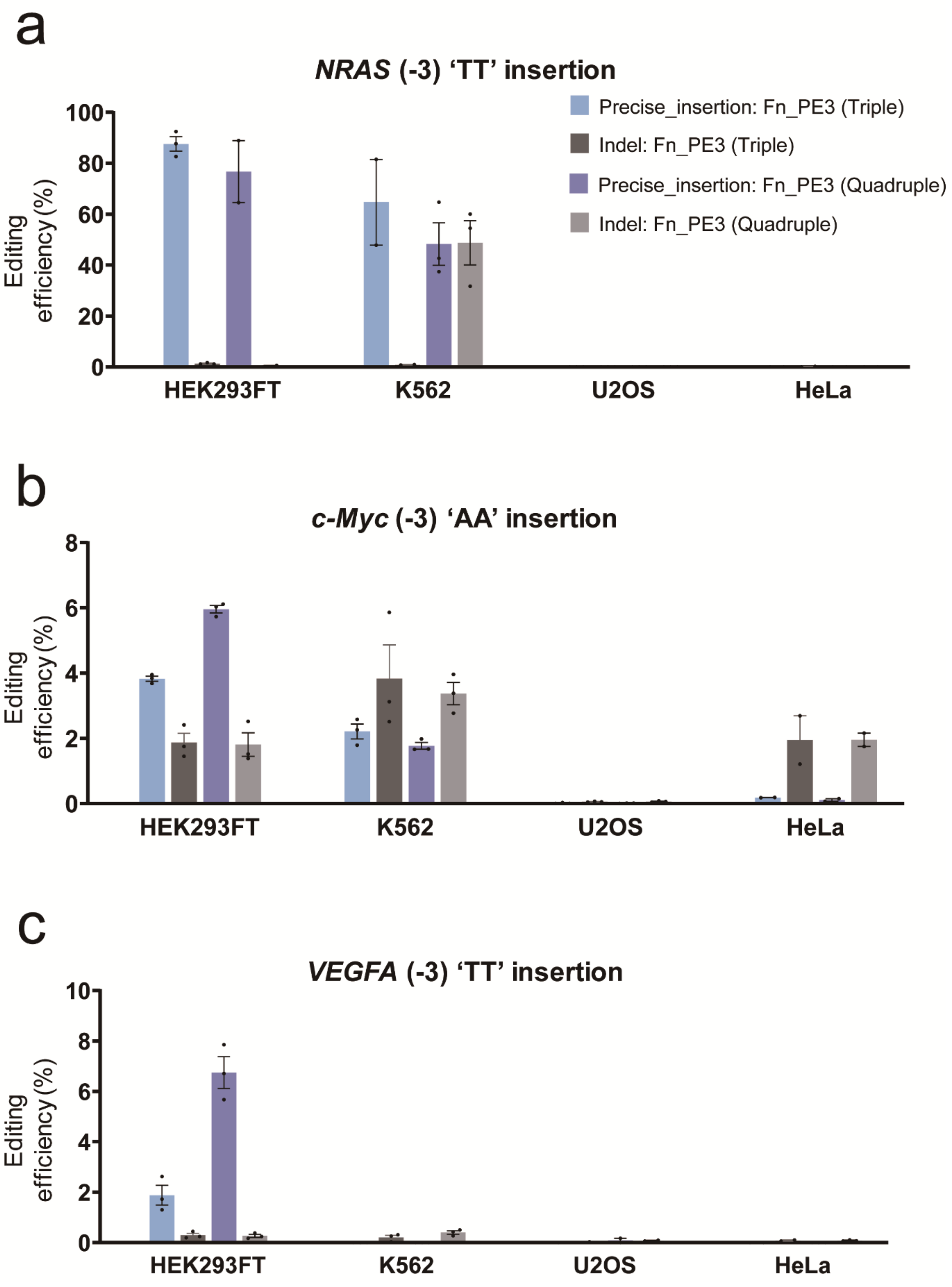

Figure S5. Cell diversity experiment of the prime editing using FnCas9(H969A)-RT. a-c, Comparison of the efficiency (%) of external nucleotide insertion on each gene (*NRAS* (a), *c-Myc* (b), *VEGFA* (c)) in various cell lines (HEK293FT, K562,

U2OS, Hela) using FnCas9(H969A)-RT. Triple: both pegRNA and ngRNA are used as FnCas9 module, Quadruple: pegRNA is used as FnCas9 and ngRNA is used as SpCas9 module, respectively. Each histogram was plotted by applying standard error of the mean values to repeated experimental values (n = 3).

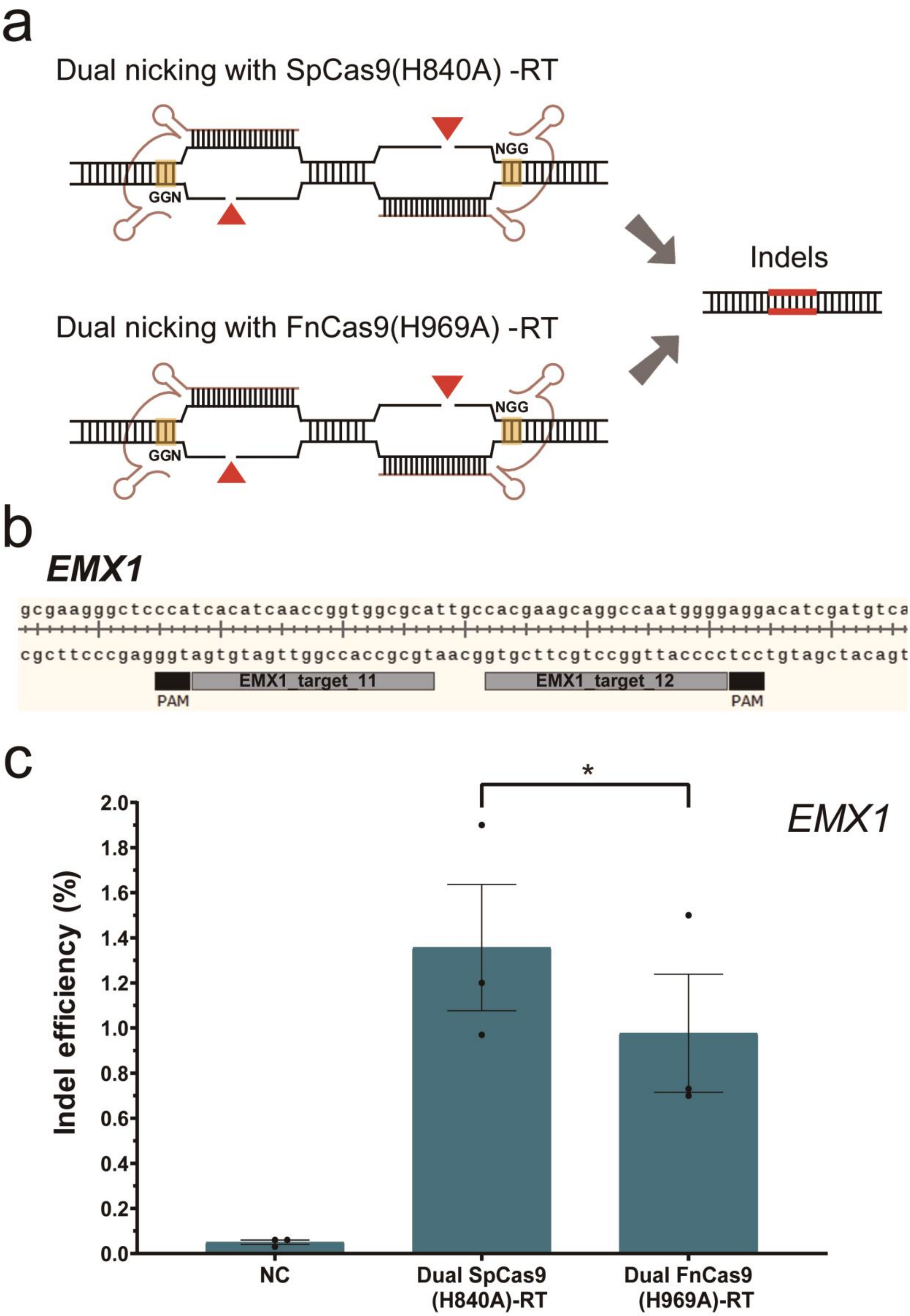

a
Dual nicking with SpCas9(H840A) -RT
GGN
NGG
Dual nicking with FnCas9(H969A) -RT
GGN
NGG
Indels
b
EMX1
gcgaagggctcccatcacatcaaccggtggcgcattgccacgaagcaggccaatggggaggacatcgatgtca
cgcttcccgagggtagtgtagttggccaccgcgtaacggtgcttcgtccggttacccctcctgtagctacagt
PAM
EMX1_target_11
EMX1_target_12
PAM
c
*
EMX1
Indel efficiency (%)
2.0
1.8
1.6
1.4
1.2
1.0
0.8
0.6
0.4
0.2
0.0
NC
Dual SpCas9 (H840A)-RT
Dual FnCas9 (H969A)-RT

Figure S6. Comparative analysis of the nickase activity of SpCas9(H840A)-RT and FnCas9(H969A)-RT. a, A schematics of a strategy to form indels in dual nickase fashion using SpCas9(H840A)-RT and FnCas9(H969A)-RT and each sgRNA. PAM(NGG) sequences are shown in yellow, and nicking points are shown in red arrowhead, respectively. **b,** Target sequences in the *EMX1* gene commonly targeted using SpCas9(H840A)-RT and FnCas9(H969A)-RT. Protospacers are shown in gray, PAM(NGG) sequences are shown in black, respectively. **c,** Comparison of NGS analysis results using SpCas9(H840A)-RT and FnCas9(H969A)-RT, forming indels(%) in dual nickase fashion. sgRNA: single-guideRNA. Each histogram was plotted by applying standard error of the mean values to repeated experimental values (n = 3). P-values are calculated using a RM-one-way ANOVA, Dunnett test (ns: not significant, *P = 0.0332, **P = 0.0021, ***P = 0.0002, ****P < 0.0001).

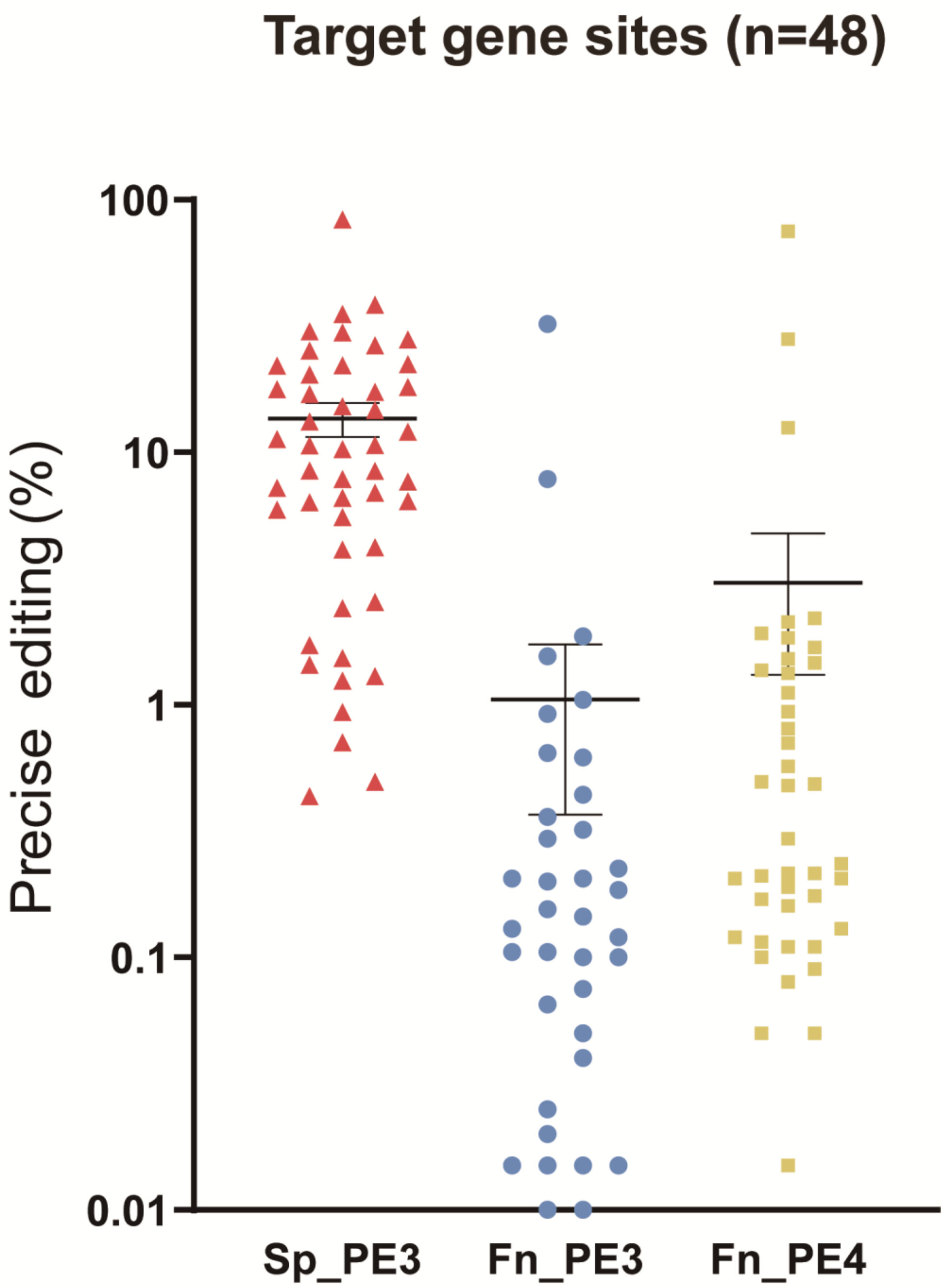

Figure S7. Targeting of the multiple loci for direct comparison between SpCas9(H840A)-RT and FnCas9(H969A)-RT based prime editing. Comparison of site-specific nucleotide insertion efficiency (%) induced using PE3 or PE4 delivery method by SpCas9(H840A)-RT and FnCas9(H969A)-RT for various target sequences (n=48, **Additional file 1: Table S1**). The histogram was plotted by applying standard error of the mean values for prime editing of 48 target sites. Sp_PE3 (Triple): Both pegRNA(prime editing guideRNA) and ngRNA(nicking

guideRNA) are used as SpCas9 modules, Fn_PE3 (Triple): Both pegRNA(prime editing guideRNA) and ngRNA(nicking guideRNA) are used as FnCas9 modules, Fn_PE4: Connected pegRNA(prime editing guideRNA) and ngRNA(nicking guideRNA) are used as FnCas9 modules.

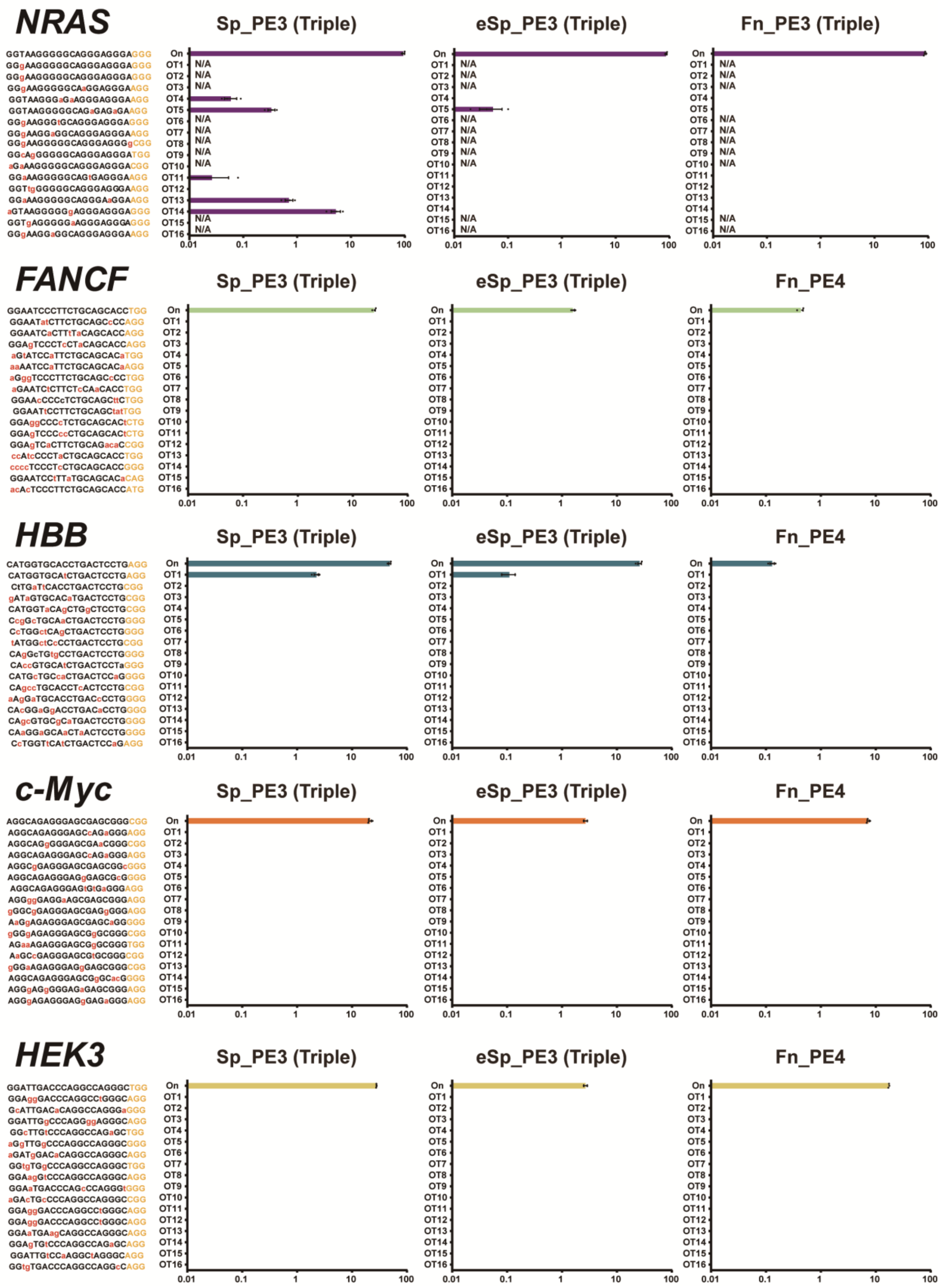

Figure S8. Comparative analysis of the off-target editing of SpCas9(H840A)-RT and FnCas9(H969A)-RT based on *in-silico* predicted sites. PAM(NGG) sequences are shown in yellow and mismatched sequences in off-target sequence

are shown in red, respectively. All histograms were analyzed with data obtained from NGS. Each histogram was plotted by applying standard error of the mean values to repeated experimental values (n = 3). On: on-target, OT: off-target, N/A: not applicable. Sp_PE3 (Triple): Both pegRNA(prime editing guideRNA) and ngRNA(nicking guideRNA) are used as SpCas9 modules, eSp_PE3 (Triple): The version of e-SpCas9 with enhanced target specificity [25] was used for prime editing, Both pegRNA (prime editing guideRNA) and ngRNA (nicking guideRNA) are used as SpCas9 modules, Fn_PE4: Connected pegRNA(prime editing guideRNA) and ngRNA(nicking guideRNA) are used as FnCas9 modules.

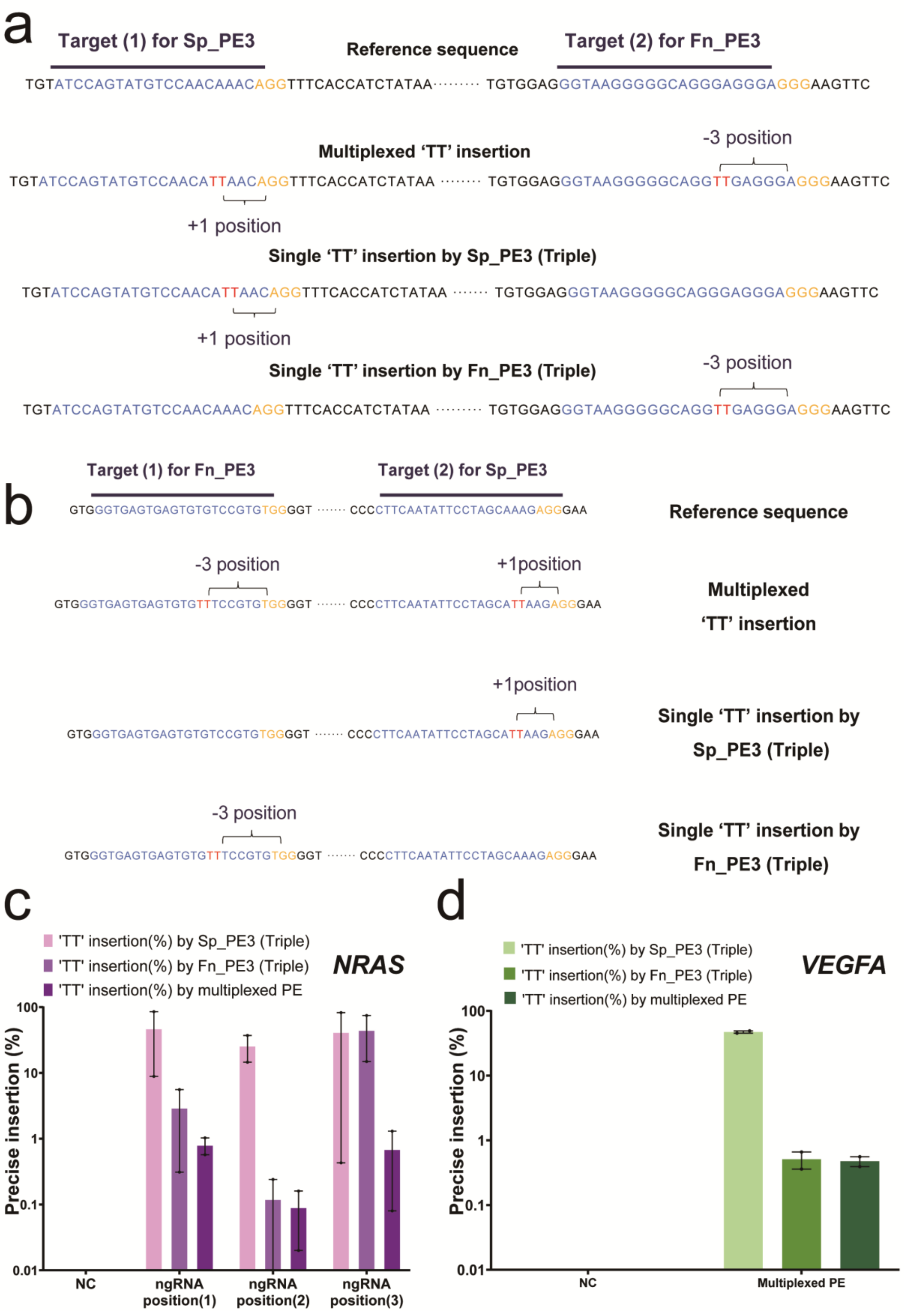

Figure S9. Multiplexed prime editing with SpCas9(H840A)-RT and FnCas9(H969A)-RT system. a, b, NGS results of multiplexed TT insertion using

SpCas9(H840A)-RT and FnCas9(H969A)-RT. Each target sequence [protospacer shown in blue and PAM (NGG) shown in yellow] for SpCas9(H840A)-RT and FnCas9(H969A)-RT is indicated. The targeted TT insertion is shown in red and each position is shown above the base. The dotted line indicates the omission of the base. PE3: prime editing with target-strand nicking; ngRNA: nicking guide RNA. **c, d,** Base insertion efficiency (%) of multiplexed prime editing at *NRAS* (c) and *VEGFA* (d) locus by co-treatment of the SpCas9(H840A)-RT and FnCas9(H969A)-RT according to ngRNA targeting at various positions (1-3, **Additional file 1: Figure S2c**). Each histogram was plotted by applying standard error of the mean values to repeated experimental values (n = 3).

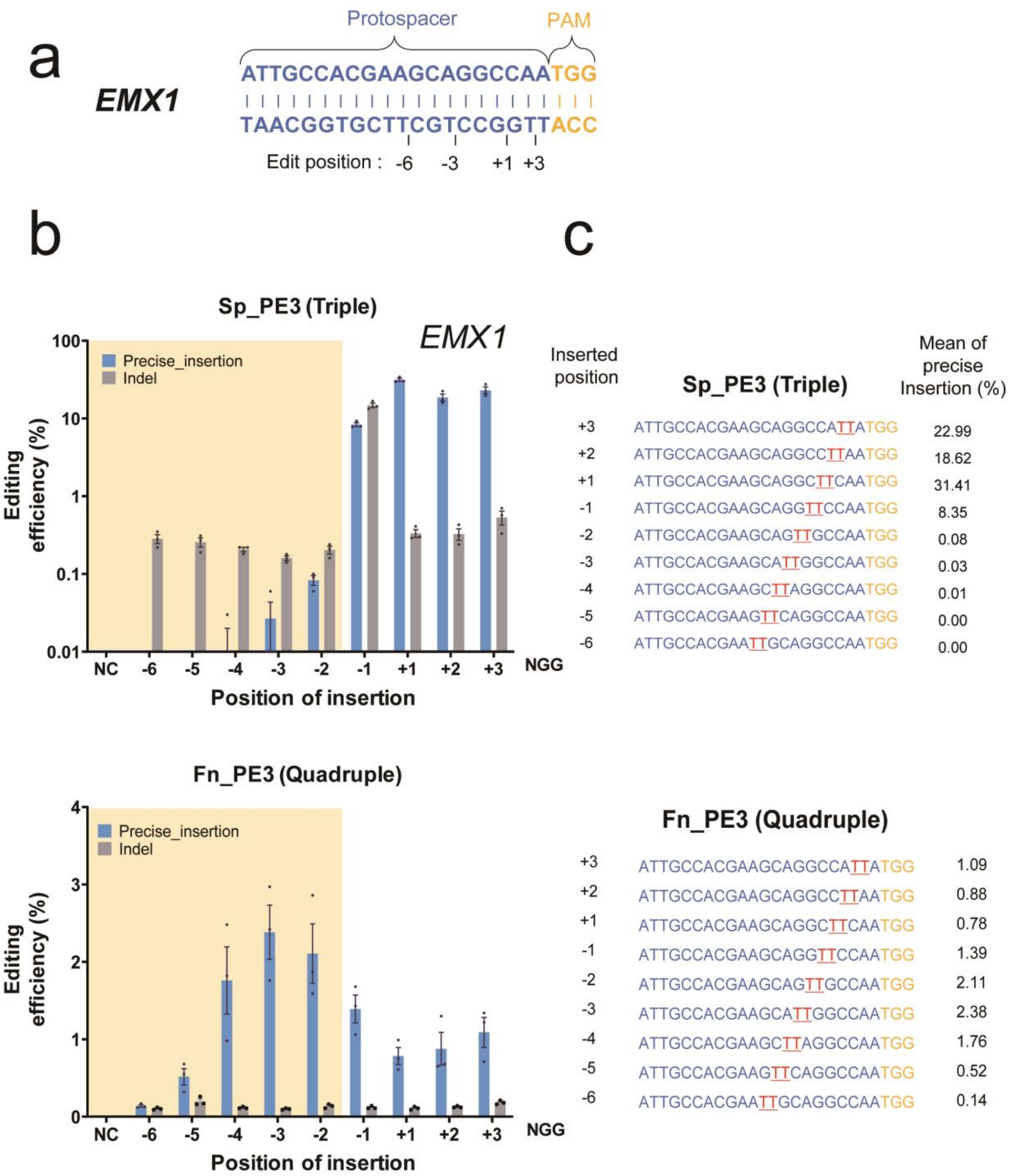

Figure S10. Expansion of the range of prime editing in *EMX1* locus by FnCas9(H969A)-RT. a, The result of site-specific base insertion induced by SpCas9(H840A)-RT and FnCas9(H969A)-RT. Protospacers are shown in blue, PAM(NGG) sequences are shown in yellow, and inserted sequence TT are shown in red, respectively. Each position and efficiency(%) in which the TT base is inserted are indicated to the left and right of the target sequence, respectively. **b,** Direct comparison of the efficiency (%) of base insertion by SpCas9(H840A)-RT and

FnCas9(H969A)-RT. Top: Comparison of efficiency (%) according to the base insertion site caused by SpCas9(H840A)-RT. Bottom: Comparison of efficiency (%) according to the base insertion site caused by FnCas9(H969A)-RT. Each histogram was plotted by applying standard error of the mean values to repeated experimental values (n = 3). **c.** NGS result of site-specific bi-nucleotide insertion induced by SpCas9(H840A)-RT and FnCas9(H969A)-RT. Each position and efficiency (%) in which the inserted TT bases are indicated to the left and right of the target sequence, respectively.

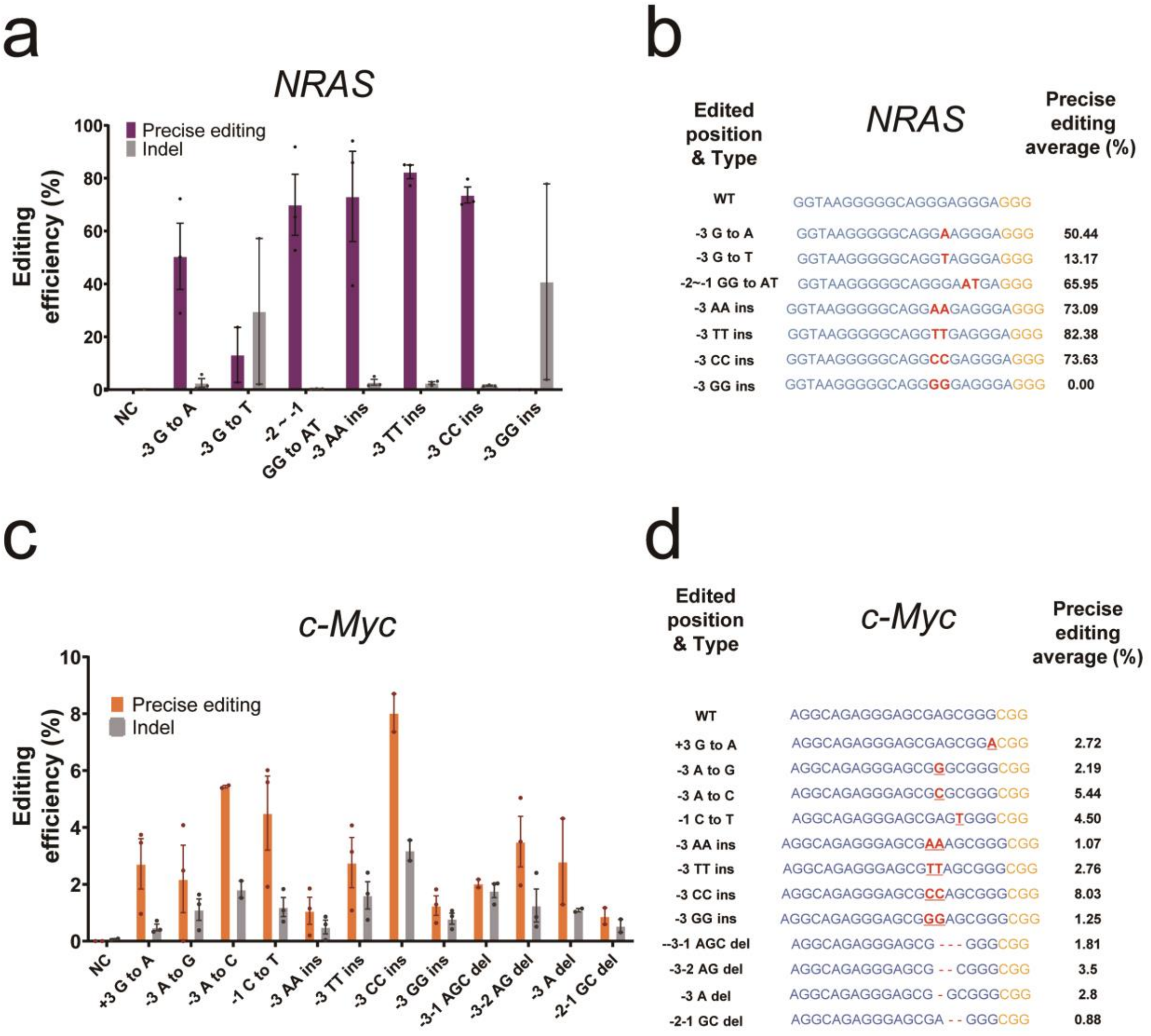

Figure S11. Result of the prime editing in target sequence induced by FnCas9(H969A)-RT. a, c, NGS result of site-specific (*NRAS* (a)*, c-Myc* (c)) nucleotide insertion induced by FnCas9(H969A)-RT. The editing efficiency (%) according to each position and type of prime editing was plotted as histogram. Each histogram was plotted by applying standard error of the mean values to repeated experimental values (n = 3). **b, d,** Sequence profiles from analyzed NGS data of targeted prime editing (a, c).

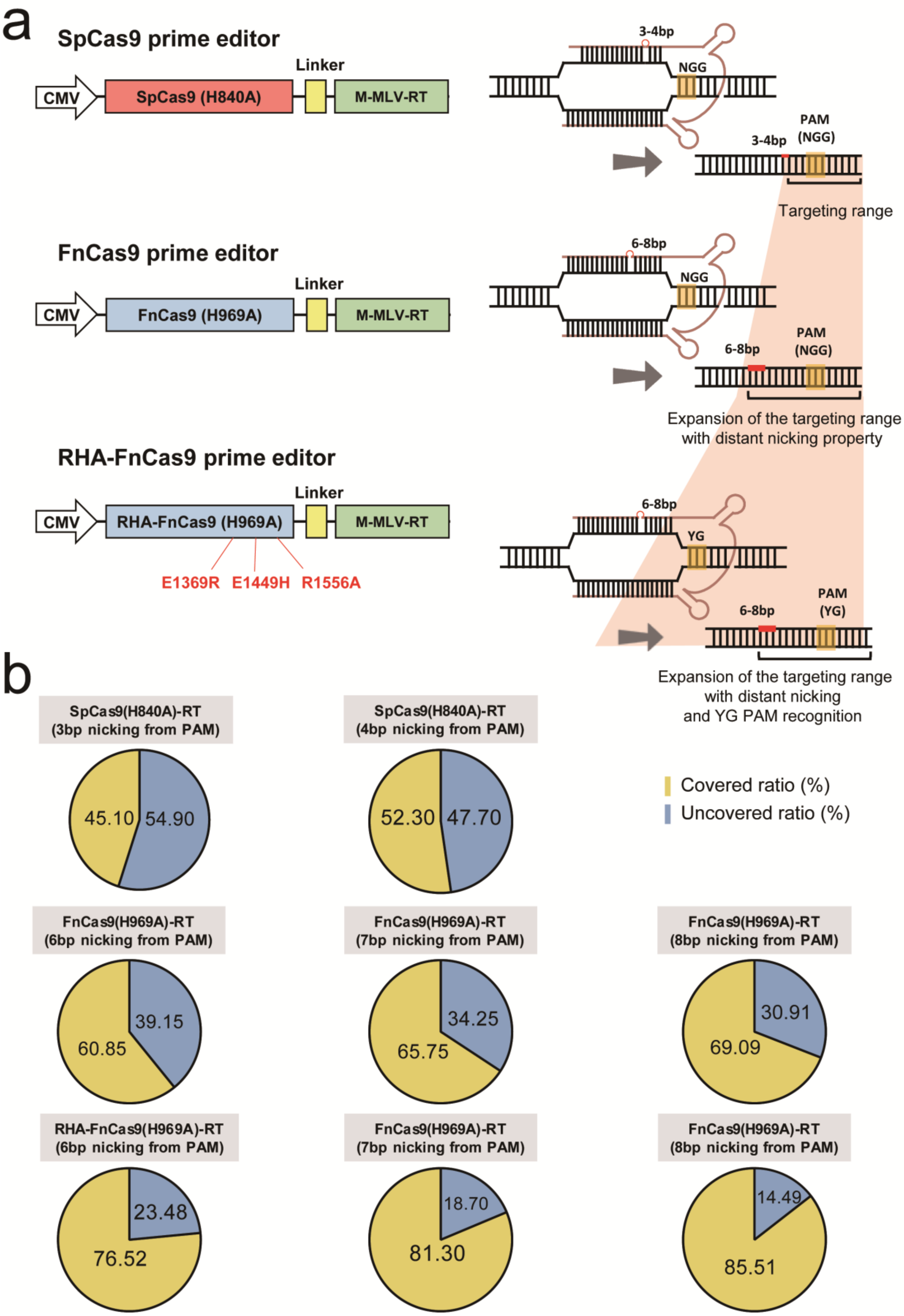
a
SpCas9 prime editor
Linker
CMV
SpCas9 (H840A)
M-MLV-RT
3-4bp
NGG
PAM
(NGG)
Targeting range
FnCas9 prime editor
Linker
CMV
FnCas9 (H969A)
M-MLV-RT
6-8bp
NGG
PAM
(NGG)
Expansion of the targeting range
with distant nicking property
RHA-FnCas9 prime editor
Linker
CMV
RHA-FnCas9 (H969A)
M-MLV-RT
E1369R
E1449H
R1556A
6-8bp
YG
PAM
(YG)
Expansion of the targeting range
with distant nicking
and YG PAM recognition
b
SpCas9(H840A)-RT
(3bp nicking from PAM)
45.10
54.90
SpCas9(H840A)-RT
(4bp nicking from PAM)
52.30
47.70
Covered ratio (%)
Uncovered ratio (%)
FnCas9(H969A)-RT
(6bp nicking from PAM)
60.85
39.15
FnCas9(H969A)-RT
(7bp nicking from PAM)
65.75
34.25
FnCas9(H969A)-RT
(8bp nicking from PAM)
69.09
30.91
RHA-FnCas9(H969A)-RT
(6bp nicking from PAM)
76.52
23.48
FnCas9(H969A)-RT
(7bp nicking from PAM)
81.30
18.70
FnCas9(H969A)-RT
(8bp nicking from PAM)
85.51
14.49

Figure S12. Expansion of the targetable range by prime editing with FnCas9(H969A)-RT or RHA-FnCas9(H969A)-RT. a, Schematics of the comparison among SpCas9(H840A)-RT, FnCas9(H969A)-RT and RHA-FnCas9(H969A)-RT based prime editing. **b,** Phi-chart demonstration for pathogenic SNP coverage of SpCas9(H840A)-RT, FnCas9(H969A)-RT and RHA-FnCas9(H969A)-RT based prime editing. The cleavage point counted as (N) bp upstream from the PAM(NGG or YG) is indicated at the top of the phi chart. Covered ratio (%) = targetable SNP number / total SNP number x100, Uncovered ratio (%) = 100 - covered ratio (%).
