# Supplementary material for: Expansion of the prime editing modality with Cas9 from Francisella novicida
Source: Genome Biol. 2022 Apr 11;23:92. doi: 10.1186/s13059-022-02644-8 (PMC8996390; doi:10.1186/s13059-022-02644-8)
Supplement: Supplementary file 2 — Additional file 2. Review history. [file 13059_2022_2644_MOESM2_ESM.docx]

Review History

**First round of review**

**Reviewer 1**

**Are you able to assess all statistics in the manuscript, including the appropriateness of statistical tests used?** No, I do not feel adequately qualified to assess the statistics.

**Comments to author:**

Prime editing is a very promising precision genome editing technology under rapid development. Authors demonstrate that FnCas9-based prime editors worked in human cells and compensated PAM-related defects. This is the first report on prime editors based on a different CRISPR/Cas system from SpCas9 and demonstrate the universality of CRISPR/Cas systems for development of prime editors. From these aspects, the report is interesting.

Minor revisions:
Since a few SpCas9 variants recognizing simple PAMs have been developed, the PAM-related targeting limitation is not a problem. Therefore, authors may need to emphasize the significance of their study from the universality of CRISPR/Cas systems for development of prime editors.

**Reviewer 2**

**Are you able to assess all statistics in the manuscript, including the appropriateness of statistical tests used?** Yes, and I have assessed the statistics in my report.

**Comments to author:**

In this study, Oh, et al. describe a simple modification to the prime editing CRISPR system to enable broader range targeting and editing by employing an orthologous Cas9, from Francisella
novicida. As compared to the standardly-used SpCas9, FnCas9 produces overhangs pattern of DSBs, and thus has a unique nicking property on the nontarget strand of protospacer. This potentially enables expansion of the edited region.

Overall, the paper is concise, well written, and to-the-point, and the methods used (plasmid construction, transfection, NGS) are straightforwards and adequate. The data works toward verifying the hypothesis that FnCas9 within the prime editing module accurately expands the targeting reach of prime editing. However, while the authors do demonstrate expansion of the targeting region, the efficiencies of editing are quite low in this extended region of various sequences tested, and the off-targeting question isn't adequately addressed. I thus request the authors address my following concerns:

Major Comments

1. To demonstrate the editing rates across unique genomic loci, I request the authors test 48 endogenous loci (the standard for most CRISPR tools) via NGS. This way, the reviewers can get a better look at how FnCas9 compares overall to SpCas9. At this point, only one chosen loci (NRAS) shows substantial editing.

2. The authors mentioned that no off-target editing was induced. I would ask the authors to conduct an unbiased screen of off-target candidates via nDigenome-Seq, followed by targeted amplicon sequencing of at least the top 16 off-target candidates (as mentioned here: https://academic.oup.com/nar/article/48/18/10576/5907964). Furthermore, if needed, I would compare SpCas9, FnCas9, and high-fidelity versions of SpCas9 in prime editor format to verify differences in off-target profile.

Minor Comments

1. I request the authors to make the figures at high resolution. It proved very difficult to read the figures due to blurriness.

**Reviewer 3**

**Are you able to assess all statistics in the manuscript, including the appropriateness of statistical tests used?** Yes, and I have assessed the statistics in my report.

**Comments to author:**

Comments to the authors
In this study, Yeounsun Oh et al expanded the editing range of prime editing by conjugating the reverse transcriptase (RT) to a nickase version of FnCas9, FnCas9 (H969A), instead of spCas9 (H840A). The authors first revealed that FnCas9 (H969A) cleaves the non-target strand at positions farther away from the "NGG" PAM sequence (6 to 8 bp upstream of PAM) compared with spCas9 (H840A) (3 to 4 bp upstream of PAM). Furthermore, the authors demonstrated that the prime editor based on FnCas9 (H969A) was capable of editing sequences at positions (from -6 to -2) that cannot be handled by the prime editor based on spCas9 (H840A). It's potentially a remarkable improvement of the prime editing technology. However, the major concern is that FnCas9 (H969A)-based prime editing manifested limited editing efficiencies at most genomic loci shown in the manuscript, which makes the prospects of its "possibility of application as a therapeutic agent" look gloomy. So, it'll be more convictive if the authors provide evidence to show that FnCas9 (H969A)-based prime editing performs outstandingly at some clinically relevant loci that are not suitable for spCas9 (H840A)-based prime editing.

P3L9 For phrase "and directly compared the cleavage results using a protospacer of the same length" the subject is missing.

P3L36 Fig. 1b doesn't support the authors' claim that "the bi-nucleotide TT was accurately inserted in the genomic DNA (c-Myc, NRAS) at a site of 6 nucleotides upstream from the PAM in the target sequence when using FnCas9(H969A)-RT".

P3L38-L39 The authors screened for optimized constitutions of the pegRNA and the FnCas9-based prime editor. I suggest that the authors make it clear which pegRNA/prime editor isoforms are used in their later experiments.

P3L51 Fig. 1e doesn't contain comparisons with spCas9 (H840A)-based prime editing.

P4L48 Fig. 3 does not exit. I recommend that the authors have a check of their citations carefully.

**Authors Response**

**Point-by-point responses to the reviewers’ comments:**

**Reviewer #1:** Prime editing is a very promising precision genome editing technology under rapid development. Authors demonstrate that FnCas9-based prime editors worked in human cells and compensated PAM-related defects. This is the first report on prime editors based on a different CRISPR/Cas system from SpCas9 and demonstrate the universality of CRISPR/Cas systems for development of prime editors. From these aspects, the report is interesting.

Minor revisions:

Since a few SpCas9 variants recognizing simple PAMs have been developed, the PAM-related targeting limitation is not a problem. Therefore, authors may need to emphasize the significance of their study from the universality of CRISPR/Cas systems for development of prime editors.

Thank you for the good comments. It helped us to improve the quality of the paper. If prime editing is applied based on an engineered module such as Sp-RY in previous research[1], the area corrected by the prime editor can be expanded. In this study, we presented as the first concept that expanding the targetable region of the prime editor is possible by the distant nicking position from the PAM in addition to the engineering method of domains unique to the Cas protein **(Main text, Figure 2)**.

In addition, we showed that *F. novicida* Cas9 ortholog has an off-target improvement effect compared to *S. pyogene* Cas9 by applying the sensitive advantage for mismatch recognition to prime editing technology **(Additional file1: Figure S8)**. As suggested by the reviewers, we noted in the discussion section of the main text that various CRISPR-Cas orthologs could be used to compensate for shortcomings in future application of prime editing in vivo.

**Reviewer #2:** In this study, Oh, et al. describe a simple modification to the prime editing CRISPR system to enable broader range targeting and editing by employing an orthologous Cas9, from Francisella

novicida. As compared to the standardly-used SpCas9, FnCas9 produces overhangs pattern of DSBs, and thus has a unique nicking property on the nontarget strand of protospacer. This potentially enables expansion of the edited region.

Overall, the paper is concise, well written, and to-the-point, and the methods used (plasmid construction, transfection, NGS) are straightforwards and adequate. The data works toward verifying the hypothesis that FnCas9 within the prime editing module accurately expands the targeting reach of prime editing. However, while the authors do demonstrate expansion of the targeting region, the efficiencies of editing are quite low in this extended region of various sequences tested, and the off-targeting question isn't adequately addressed. I thus request the authors address my following concerns:

Major Comments

1. To demonstrate the editing rates across unique genomic loci, I request the authors test 48 endogenous loci (the standard for most CRISPR tools) via NGS. This way, the reviewers can get a better look at how FnCas9 compares overall to SpCas9. At this point, only one chosen loci (NRAS) shows substantial editing.

Thank you very much for the constructive comments. As suggested by the reviewer, we analyzed the prime editing efficiency of 48 common target sequences in various genes to directly compare the effects of SpCas9 and FnCas9-based prime editing, respectively **(Additional file1: Figure S7)**. As a result, compared to SpCas9-based prime editing, the efficiency of FnCas9 showed a large standard deviation according to genomic locus.These results are thought to be due to traits that are highly influenced by chromosome topology, such as the previously reported FnCas9-related paper[2]. As shown in the results of **(Figure 1e)**, FnCas9-based prime editing appears to be significantly affected by the nicking position and efficiency induced on the target-strand side. Accordingly, in order to expect high efficiency, it is thought that the nicking position induced on the target-strand side should be optimized according to the target nucleotide sequence.

We presented the possibility of improving FnCas9-based prime editing by using the previously reported method[3] that improved prime editing efficiency **(Main text, Figure 1h-k).** And we mentioned in the discussion section about ways to improve prime editing efficiency by improving the nicking function of the non-target strand of the FnCas9 protein or using an effective delivery method such as a virus in the near future **(Main text page6, lane160-163)**.

2. The authors mentioned that no off-target editing was induced. I would ask the authors to conduct an unbiased screen of off-target candidates via nDigenome-Seq, followed by targeted amplicon sequencing of at least the top 16 off-target candidates (as mentioned here: https://academic.oup.com/nar/article/48/18/10576/5907964). Furthermore, if needed, I would compare SpCas9, FnCas9, and high-fidelity versions of SpCas9 in prime editor format to verify differences in off-target profile.

Thank you very much for the constructive experimental suggestions and comments. We read the proposed paper in detail, but unfortunately, there were considerable difficulties in conducting and analyzing experiments in-vitro because the nDigenome-Seq method[4] was optimized for specific laboratories and conditions. In particular, since nDigenome-Seq is a method to detect the off-target characteristics of CRISPR nickase outside the cell unlike Guide-seq, we are doubt that nDigenome-Seq reflects the exact off-target according to prime editing in cellular system. In addition, in the nDigenome-seq paper presented, only the indirect effect of prime editing was confirmed using nickase (SpCas9 (H840A)) that are not connected to RT.

Therefore, we induced SpCas9, e-SpCas9 and FnCas9-based prime editing for 5 target sequences including the target selected in the nDigenome-Seq paper in parallel, and analyzed the top 16 off-target sequences for each target sites **(Additional file1: Figure S8)**. We compared and analyzed the off-targeting properties between prime editors using targeted amplicon sequencing. As a result, it was confirmed that prime editing induced based on FnCas9 works accurately in the targeted sequences.

Minor Comments

1. I request the authors to make the figures at high resolution. It proved very difficult to read the figures due to blurriness.

All figures were converted to high resolution and uploaded to the system.

**Reviewer #3:** Comments to the authors

In this study, Yeounsun Oh et al expanded the editing range of prime editing by conjugating the reverse transcriptase (RT) to a nickase version of FnCas9, FnCas9 (H969A), instead of spCas9 (H840A). The authors first revealed that FnCas9 (H969A) cleaves the non-target strand at positions farther away from the "NGG" PAM sequence (6 to 8 bp upstream of PAM) compared with spCas9 (H840A) (3 to 4 bp upstream of PAM). Furthermore, the authors demonstrated that the prime editor based on FnCas9 (H969A) was capable of editing sequences at positions (from -6 to -2) that cannot be handled by the prime editor based on spCas9 (H840A). It's potentially a remarkable improvement of the prime editing technology. However, the major concern is that FnCas9 (H969A)-based prime editing manifested limited editing efficiencies at most genomic loci shown in the manuscript, which makes the prospects of its "possibility of application as a therapeutic agent" look gloomy. **So, it'll be more convictive if the authors provide evidence to show that FnCas9 (H969A)-based prime editing performs outstandingly at some clinically relevant loci that are not suitable for spCas9 (H840A)-based prime editing.**

Thank you very much for the constructive comments. We recognized that FnCas9-based prime editing efficiency was lower than that of SpCas9 on average, and to overcome this problem, the intracellular co-expression system of pegRNA and ngRNA was optimized for FnCas9 based prime editing. For a specific gene (*c-Myc*), the prime editing efficiency was significantly restored **(Main text, Figure 1h-k)**. In particular, by using the point where FnCas9 induced nicking is farther from PAM than nick by SpCas9, it was possible to induce editing in areas where prime editing was not possible based on SpCas9 **(Main text, Figure 2a-c, yellow boxed region)**. In addition, by applying RHA-FnCas9-based prime editing through previously reported Cas9 domain engineering[5], a simple PAM sequence (YG) was recognized to expand the targetable region. For example, it was shown that the *c-Myc* gene, which is continuously expressed in cancer cells, can be more effectively regulated by using RHA-FnCas9-based prime editing than SpCas9 based one **(Main text, Figure 2d-g)**.

We mentioned in the discussion section about the possibility of applying FnCas9-based prime editing to human diseases caused by various genetic mutations, in the form of pathogenic SNPs or indels, when using effective carriers such as viruses in the near future.

P3L9 For phrase "and directly compared the cleavage results using a protospacer of the same length" the subject is missing.

We have corrected the main text.

P3L36 Fig. 1b doesn't support the authors' claim that "the bi-nucleotide TT was accurately inserted in the genomic DNA (c-Myc, NRAS) at a site of 6 nucleotides upstream from the PAM in the target sequence when using FnCas9(H969A)-RT".

It has been deleted from the main figure as unnecessary content.

P3L38-L39 The authors screened for optimized constitutions of the pegRNA and the FnCas9-based prime editor. I suggest that the authors make it clear which pegRNA/prime editor isoforms are used in their later experiments.

For each figure, the expression and delivery method of pegRNA and SpCas9 or FnCas9-based prime editor was described in detail, and it was additionally described in an Supplementary Information **(Additional file1: Figure S3).**

P3L51 Fig. 1e doesn't contain comparisons with spCas9 (H840A)-based prime editing.

At the request of reviewer 2, we directly compared SpCas9 and FnCas9 for 48 target sequences, including the target sequence in Figure 1.

P4L48 Fig. 3 does not exit. I recommend that the authors have a check of their citations carefully.

We have corrected the main text.

**References**

1.	Walton RT, Christie KA, Whittaker MN, Kleinstiver BP: **Unconstrained genome targeting with near-PAMless engineered CRISPR-Cas9 variants.** *Science* 2020, **368:**290-296.

2.	Chen F, Ding X, Feng Y, Seebeck T, Jiang Y, Davis GD: **Targeted activation of diverse CRISPR-Cas systems for mammalian genome editing via proximal CRISPR targeting.** *Nat Commun* 2017, **8:**14958.

3.	Liu Y, Yang G, Huang S, Li X, Wang X, Li G, Chi T, Chen Y, Huang X, Wang X: **Enhancing prime editing by Csy4-mediated processing of pegRNA.** *Cell Res* 2021, **31:**1134-1136.

4.	Kim DY, Moon SB, Ko JH, Kim YS, Kim D: **Unbiased investigation of specificities of prime editing systems in human cells.** *Nucleic Acids Res* 2020, **48:**10576-10589.

5.	Hirano H, Gootenberg JS, Horii T, Abudayyeh OO, Kimura M, Hsu PD, Nakane T, Ishitani R, Hatada I, Zhang F, et al: **Structure and Engineering of Francisella novicida Cas9.** *Cell* 2016, **164:**950-961.

**Second round of review**

**Reviewer 2**

The authors have addressed all of my concerns, and I am happy to recommend publication of the manuscript.

**Reviewer 3**

The authors have addressed my comments.
